# 1,2-Metallate Rearrangement as a Toolbox for the Synthesis of Allylic Alcohols

**DOI:** 10.1021/acs.joc.3c01309

**Published:** 2023-08-18

**Authors:** Yannick Linne, Daniel Lohrberg, Henry Struwe, Elvira Linne, Anastasia Stohwasser, Markus Kalesse

**Affiliations:** † Institute of Organic Chemistry, Gottfried Wilhelm Leibniz Universität Hannover, 30167 Hannover, Germany; ‡ Centre of Biomolecular Drug Research (BMWZ), Gottfried Wilhelm Leibniz Universität Hannover, 30167 Hannover, Germany

## Abstract

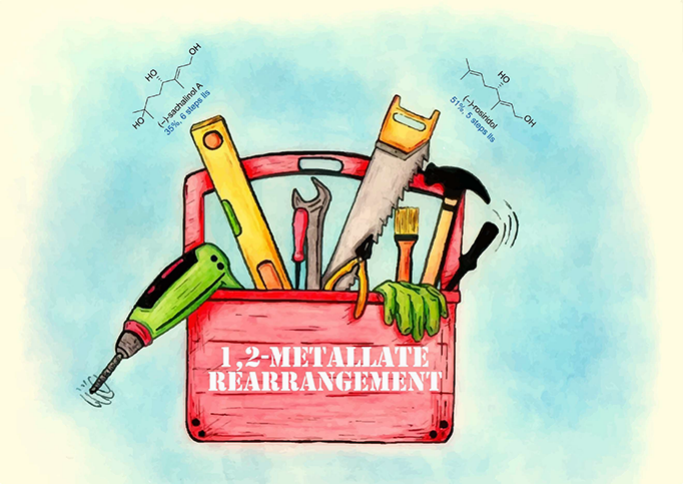

The development of new methods and protocols for the synthesis of biologically active substances remains one of the most important pillars in organic chemistry, and one of these privileged structural motifs are allylic alcohols. The method of choice to date for the synthesis of these is the Nozaki–Hiyama–Takai–Kishi reaction. We describe here a valuable alternative to the synthesis of allylic alcohols via 1,2-metallate rearrangement. In this work, various vinyl boronic esters with different functional groups have been applied in the Hoppe–Matteson–Aggarwal reaction. In addition, two monoterpenoids were constructed via this convergent synthetic strategy.

## Introduction

**Scheme 1:**
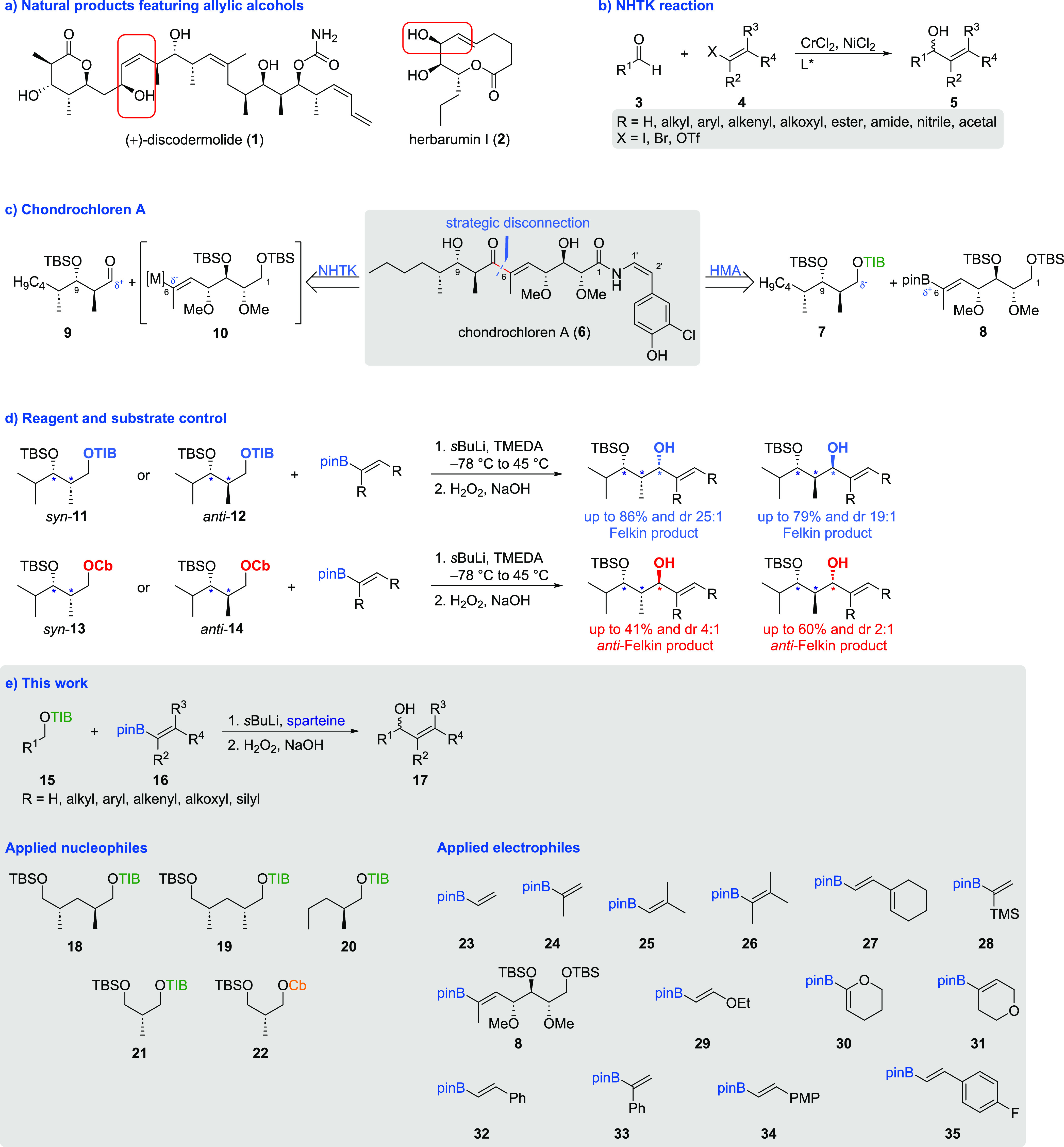
(a) Natural Products Featuring Allylic Alcohols, (b) General Scheme of the NHTK reaction, (c) Strategic Disconnection in the Total Synthesis of Chondrochloren A, (d) Substrate- and Reagent-Controlled Synthesis of Stereotriads, and (e) Applicability of the HMA Protocol to Different Systems

Allylic alcohols are important structural motifs found in numerous bioactive natural products (Scheme 1a).^1^ The current state-of-the-art approach for the stereoselective construction of allylic alcohols relies on organometal additions like the Nozaki–Hiyama–Takai–Kishi reaction (NHTK reaction, Scheme 1b).^2^ Despite the high substrate tolerance, the NHTK reaction often provides poor to moderate yields and selectivities, especially for larger and more complex fragments.^2e,2f,3^ In 2021, during our synthesis of chondrochloren A (**6**),^4^ we found a remarkable alternative to the NHTK protocol via the Hoppe–Matteson–Aggarwal (HMA) protocol^5^ using 1,2-metallate rearrangements. The reaction of the Hoppe anion derived from 2,4,6-triisopropylbenzoyl (TIB) ester **7** with vinyl boronic ester **8** followed by oxidative workup afforded the corresponding allylic alcohol in a very good yield (85%) and excellent selectivity (one diastereoisomer). This setup mimics the NHTK reaction^2^ albeit with reversed polarities, where the alcohol is masked as a Hoppe anion acting as the nucleophile, while the vinyl compound functions as the electrophile (Scheme 1c).^4^ Recently, we got mechanistic insights for reasoning of the reagent and substrate control observed for the first time in the chondrochloren A (**6**) synthesis, making this disconnection approach even more useful (Scheme 1d).^6^ In order to gain more insights, the tolerance to different functional groups and thus the broad applicability of this new protocol were investigated. Therefore, we have transformed different vinyl boronic esters to the corresponding allylic alcohols (Scheme 1e).

To minimize the influence of the TIB esters and carbamates on the substrate tolerance, simpler nucleophiles were used compared to TIB esters **7**, **11**, and **12**. Nevertheless, the TIB esters and *N*,*N*-diisopropyl carbamoyl (Cb) analogues were designed to reflect the situation in polyketide frameworks. For example, TIB esters **18**(7) and **19** show the commonly occurring 1,3-deoxypropionate motif,^8^ whereas TIB ester **21**,^9^ the corresponding carbamate **22,**(10) and TIB ester **20**(11) show the simplest representatives with an α-stereocenter (Scheme 1e). To ensure the highest possible diversity of functional groups, vinyl boronic esters bearing different substituents, such as alkyl groups (**23-27**, different substitution patterns), ethers/enol ethers (**29-31**), and aromatic residues (**32-35**) were used (Scheme 1e).

## Results and Discussion

**Scheme 2:**
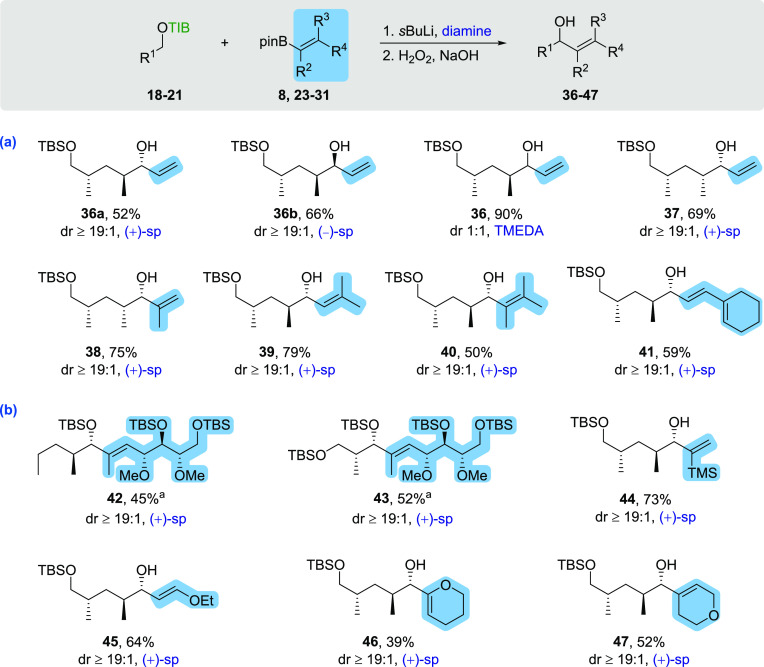
(a) Scope of the Aliphatically Substituted Vinyl Boronic Esters and (b) Scope of Complex Vinyl Boronic Esters General conditions: (1) TIB ester (1.5 equiv), diamine (1.5 equiv), *s*BuLi (1.4 equiv), Et_2_O, −78 °C, 5 h then vinyl boronic ester (1.0 equiv), Et_2_O, −78 °C, 3 h then 45 °C, o/n. (2) H_2_O_2_, NaOH, THF, −20 °C to rt. Yield given over three steps after subsequent TBS protection.

At the beginning of our studies, vinyl boronic esters **23-27**(12) with different aliphatic substitution patterns were employed (Scheme 2a). The examined aliphatic substituents showed excellent selectivities and good to very good yields over two steps (50–79%). In general, (+)- and (−)-sparteine as well as the 1,3-*syn*- and 1,3-*anti*-methyl groups did not affect the excellent stereoselectivity (≥19:1) in any matched or mismatched cases. A tendency of increasing yield with the degree of substitution was identified (52–79%). The decisive factor for this observation is probably the enhanced migration ability in the 1,2-metallate rearrangement due to the inductive effect of the aliphatic substituents. Only fully substituted vinyl boronic ester **26** stands out by showing a comparable yield to unsubstituted **23** (50% vs 52%), possibly due to the steric hindrance in the ate-complex overruling the increased migration ability.

Subsequently, more complex vinyl boronic esters, for example, with larger substituents, or enol ether derivatives were used in the 1,2-metallate rearrangement (Scheme 2b). The complex vinyl boronic ester **8**(4) used in the total synthesis of chondrochloren A was also successfully reacted with TIB esters **20**(11) and **21**.^9^ The yields in these cases are given over three steps, including subsequent TBS protection in order to achieve a more convenient purification. The use of the TMS-branched vinyl boronic ester **28**(13) gave allylic alcohol **44** in a very good yield of 73% over two steps (o2s). After protection of the generated alcohol, the vinylsilane unit in **44** could be used in numerous reactions such as electrophilic substitutions, Heck-like reactions, or transmetalations and could thus represent an important building block in total synthesis.^14^ In addition to the vinylsilane unit, enol ether functions were also introduced. Here, the observed yield of the cyclic enol ether **46** (39%) was significantly lower than that of the open-chain enol ether **45** (64%). The reaction of TIB ester **18**(7) with vinyl boronic ester **31**(12) allowed the introduction of a dihydropyran ring in moderate to good yield over two steps (52%).

**Scheme 3:**
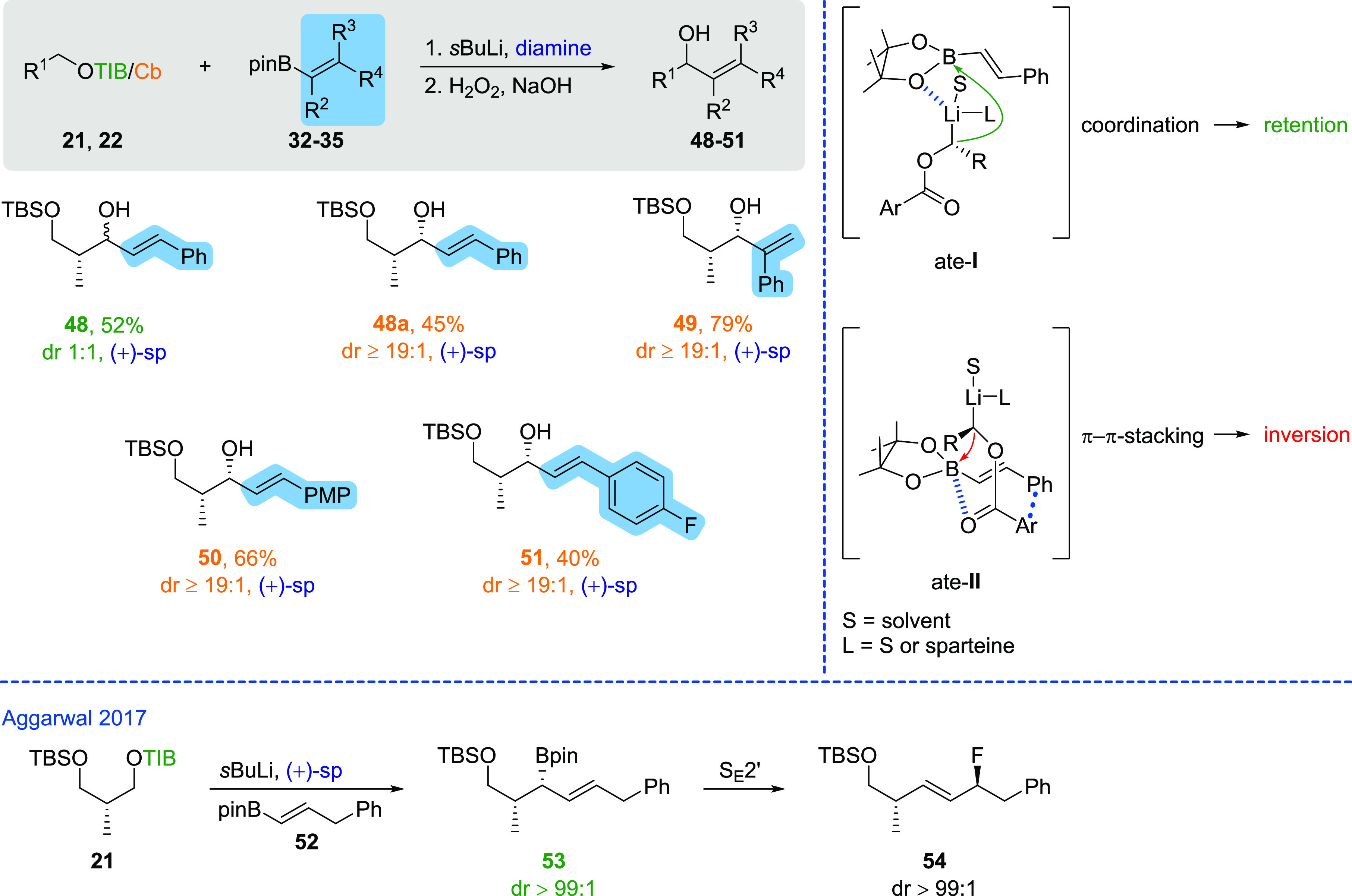
Scope of Aromatic Vinyl Boronic Esters and Explanation for Inversion versus Retention General conditions: (1) TIB ester (1.5 equiv), diamine (1.5 equiv), *s*BuLi (1.4 equiv), Et_2_O, −78 °C, 5 h then vinyl boronic ester (1.0 equiv), Et_2_O, −78 °C, 3 h then 45 °C, o/n or carbamate (1.5 equiv), diamine (1.5 equiv), *s*BuLi (1.4 equiv), Et_2_O, −78 °C, 5 h then vinyl boronic ester (1.0 equiv), Et_2_O, −78 °C, 3 h then MgBr_2_•OEt_2_ (2.0 equiv), −78 °C, 30 min then 45 °C, o/n. (2) H_2_O_2_, NaOH, THF, −20 °C to rt.

A remarkable observation was made when vinyl boronic esters with aromatic substituents were used in the 1,2-metallate rearrangement (Scheme 3) in combination with TIB esters. The reaction of TIB ester **21**(9) with vinyl boronic ester **32**(12) in the presence of (+)-sparteine afforded the corresponding allylic alcohol **48** in a diastereomeric mixture of 1:1, whereas the use of the corresponding Cb-analogue **22**(10) afforded **48a** in an excellent selectivity (≥19:1) and moderate yield (45%). The low selectivity in the case of the TIB ester could be attributed to π–π interactions between the TIB group and the aromatic substituent of the vinyl boronic ester. As a result of this attractive interaction, the seven-membered transition state ate-**II** could occur during ate-complex formation, which would favor the ate-complex formation under inversion. In 2017, the group of Aggarwal investigated the S_E_2′ reaction of allyl-boronates by treating the Hoppe anion derived from TIB ester **21** in the presence of (+)-sparteine with elongated vinyl boronic ester **52**.^8^ In contrast to our observations with the directly substituted vinyl boronic esters, they did not observe an inversion process. Therefore, it can be assumed that both the direct conjugation to the double bond and the enormous rigidity are crucial for the inversion. This inversion process probably runs in strong competition with the usually observed retention and could explain the low selectivity for the TIB ester. This attractive interaction is not possible in the case of the Cb group; therefore, no inversion process can occur here, and accordingly, the excellent selectivity (≥19:1) induced by (+)-sparteine is maintained. Based on these observations, the other vinyl boronic esters with aromatic substituents were converted with carbamate **22**(10) only. Thereby, in the case of vinyl boronic ester **33**,^12^ allylic alcohol **49** was prepared in a very good yield (79% o2s) and in an excellent selectivity (≥19:1). In addition to the simple phenyl substituent, electron-rich and -poor aromatic substituents were introduced in moderate to good yields (**51** 40%, **50** 66%).

**Scheme 4:**
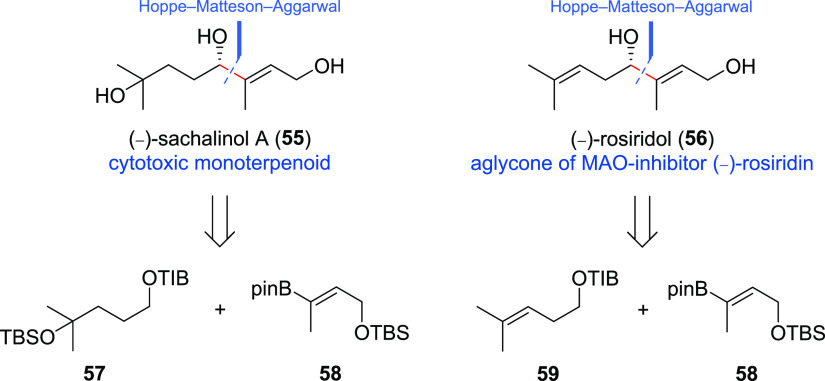
Structures and Retrosynthetic Analyses of (−)-Sachalinol A (**55**) and (−)-Rosiridol (**56**)

In order to show that this chemistry can be applied to natural product classes other than polyketides, the two monoterpenoids (−)-sachalinol A (**55**)^15^ and (−)-rosiridol (**56**)^15a,16^ were constructed via our Hoppe–Matteson–Aggarwal protocol (Scheme 4). The group of sachalinols are secondary metabolites of the perennial herbaceous plant **Rhodiola rosea** (golden root) and **Rhodiola sachalinensis**.^15a,17^ Sachalinol A (**55**) was first described by the group of Kadota in 2001.^15a^ The strong cytotoxic property and good anticancer activity^18^ make Sachalinol A (**55**) an attractive synthetic target. Rosiridol (**56**) is the aglycone of the MAO inhibitor rosiridin and thus also a synthetically valuable target.^19^ The synthesis of these two natural products will be carried out via a convergent approach of the previously described Hoppe–Matteson–Aggarwal protocol, requiring in both cases the literature-known vinyl boronic ester **58**.^20^

**Scheme 5:**
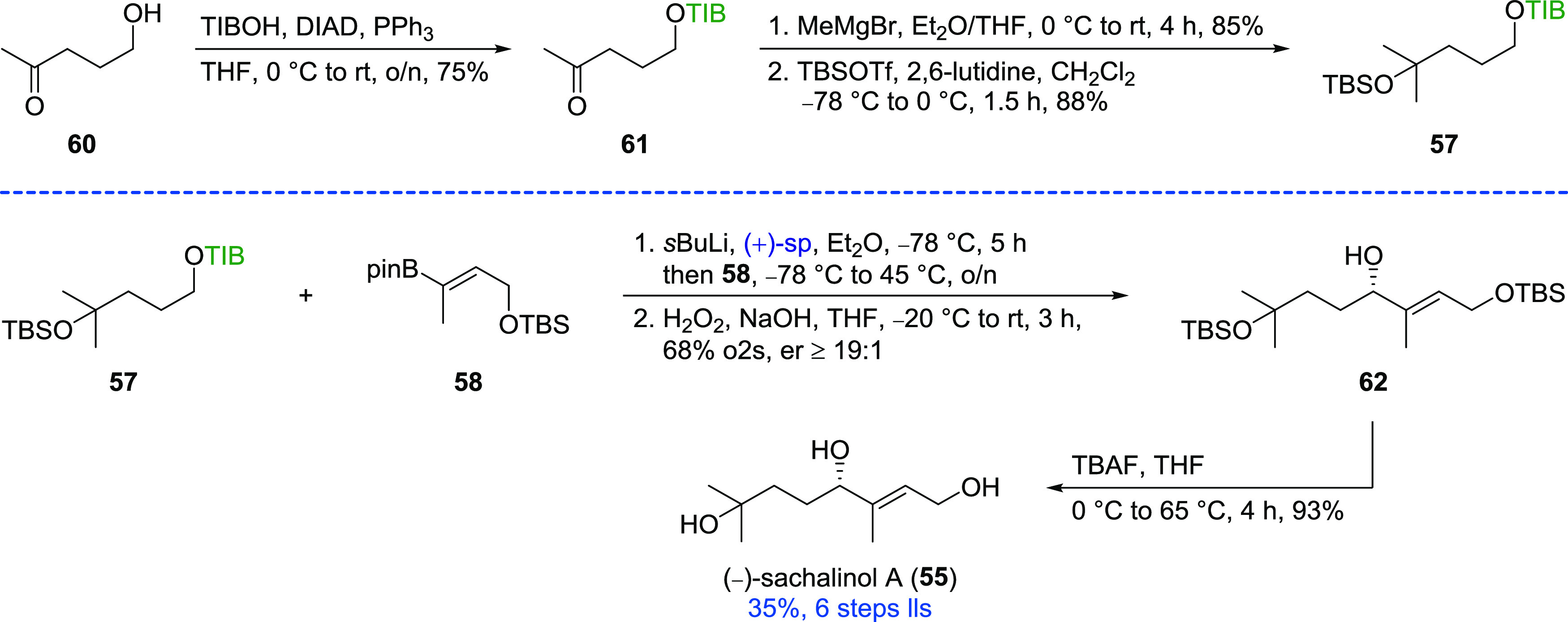
Synthesis of (−)-Sachalinol A (**55**)

The first step in the synthesis of TIB ester **57** was the introduction of the TIB group via a Mitsunobu reaction^21^ starting from commercially available γ-hydroxy ketone **60** (Scheme 5). Addition of methyl magnesium bromide provided the corresponding tertiary alcohol, which was subsequently TBS protected. Deprotonation of TIB ester **57** with *s*BuLi in the presence of (+)-sparteine and subsequent treatment with vinyl boronic ester **58** (78% o2s)^20^ afforded allylic alcohol **62** in a good yield of 68% over two steps and in excellent selectivity (≥19:1). The synthesis of sachalinol A (**55**) was completed by global deprotection using TBAF in an overall yield of 35% (6 steps lls).

**Scheme 6:**
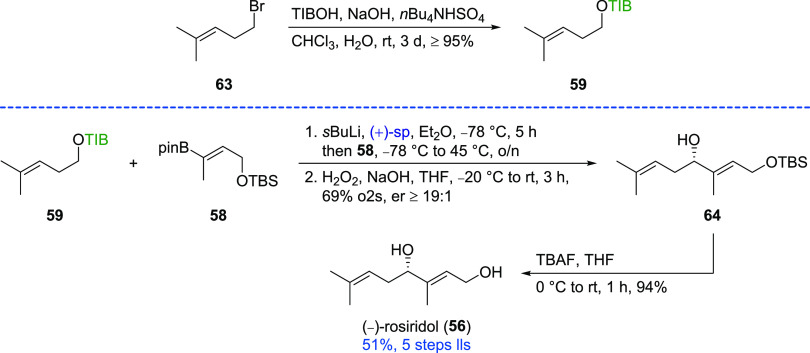
Synthesis of (−)-Rosiridol (**56**)

The synthesis of TIB ester **59** required for the synthesis of (−)-rosiridol (**56**) was carried out under phase transfer conditions^22^ with commercially available homoallylic bromide **63** (Scheme 6). The subsequent reaction of the Hoppe anion generated in the presence of *s*BuLi and (+)-sparteine with vinyl boronic ester **58** (78% o2s)^20^ afforded allylic alcohol **64** in a very good yield of 69% after oxidation. Deprotection of the primary TBS ether with TBAF afforded (−)-rosiridol (**56**) in an overall yield of 51% (5 steps lls).

## Conclusions

In conclusion, our protocol of the Hoppe–Matteson–Aggarwal chemistry allowed us to convert a broad range of vinyl boronic esters into allylic alcohols in consistently good yields and excellent selectivities. Only the vinyl boronic esters featuring aromatic substituents in combination with the TIB group were an exception. Here, in addition to the usual retention, inversion was also observed during ate-complex formation. This observation clearly contradicts the established thesis that boronic esters react exclusively under retention.^23^ However, the use of the corresponding carbamates again led to the usual excellent selectivities. Moreover, the applicability of our protocol in convergent synthesis planning was impressively demonstrated by the total synthesis of two monoterpenoids. Thus, to the best of our knowledge, our synthesis of sachalinol A (**55**) represents the shortest synthesis of this cytotoxic compound to date.^18,24^ Accordingly, this protocol of the Hoppe–Matteson–Aggarwal chemistry represents a synthetically very valuable alternative to the NHTK reaction.

## Data Availability

The data underlying this study are available in the published article and its Supporting Information.

## Supporting Information Available

Experimental procedures, NMR spectra, and compound characterizations PDF

## Abbreviations

Cb: *N,N*-diisopropylcarbamoyl
MAO: monoamine oxidase
pin: pinacolato
PMP: *para*-methoxyphenyl
sp: sparteine
TBS: *tert*-butyldimethylsilyl
TIB: 2,4,6-triisopropylbenzoyl
TMEDA: *N,N,N′,N*′**-tetramethylethylenediamine
TMS: trimethylsilyl
