# Supplementary material for: 1,2-Metallate Rearrangement as a Toolbox for the Synthesis of Allylic Alcohols
Source: J Org Chem. 2023 Aug 18;88(17):12623–9. doi: 10.1021/acs.joc.3c01309 (PMC10476192; doi:10.1021/acs.joc.3c01309)
Supplement: Supplementary file 1 — jo3c01309_si_001.pdf [file jo3c01309_si_001.pdf]

Supporting Information

1,2-Metallate Rearrangement as a Toolbox for the Synthesis of Allylic Alcohols

Yannick Linne, Daniel Lohrberg, Henry Struwe, Elvira Linne, Anastasia Stohwasser, and Markus Kalesse*

Institute of Organic Chemistry, Gottfried Wilhelm Leibniz Universität Hannover, Schneiderberg 1B, 30167 Hannover (Germany)

Corresponding Author

*markus.kalesse@oci.uni-hannover.de

Table of Contents

1. General Considerations..........S2

2. Experimental Procedures and Characterization Data..........S4

2-1. Synthesis of TIB esters..........S4

2-2. Synthesis of carbamates..........S6

2-3. 1,2-metallate rearrangement of vinyl boronates..........S6

2-4. Synthesis of (–)-sachalinol A..........S19

2-5. Synthesis of (–)-rosiridol..........S21

2-6. Analysis of enantioselectivity..........S23

3. References..........S24

4. Spectra..........S26

1. General Considerations

Unless otherwise noted all reactions were carried out under an argon atmosphere using a Drierite™ gas-drying unit. The used glassware was flame dried under high vacuum. Air- and moisture-sensitive liquids and solutions were transferred via syringe flushed with argon prior to use. All reagents were purchased from commercial suppliers and used without further purification unless otherwise noted. Vinylboronic acid pinacol ester (**23**) was bought from Sigma Aldrich and Alfa Aesar and was distilled prior to use. **(+)-Sparteine** was purchased from Chem-Impex and **(−)-sparteine** was bought from TCI. Both were distilled under high vacuum and stored under argon at −25 °C. Stated temperatures, except room temperature, refer to bath temperatures (heating was conducted using oil baths).

Dry solvents Dichloromethane and all amine bases were distilled under an inert atmosphere over calcium hydride. Tetrahydrofuran, diethyl ether and methanol were purchased from Acros Organics over molecular sieves and under inert atmosphere.

Thin layer chromatography All reactions were stirred magnetically and monitored using pre-coated TLC sheets ALUGRAM® Xtra SIL G/UV_{254} (0.2 mm, silica gel, F_{254}, aluminum-backed, MACHEREY-NAGEL) with detection by UV light (λ = 254 nm) and/or by staining with either basic potassium permanganate, acidic ceric ammonium molybdate, acidic anisaldehyde or acidic vanillin stain.

Flash column chromatography was performed using silica gel (0.04-0.063 mm, 240-400 mesh) obtained from MACHEREY-NAGEL. The applied petroleum ether fraction had a bp of 40-60 °C. The eluent is given in volume ratios (v/v).

^{1}H-NMR experiments were recorded in $CDCl_3$ or C_6D_6 using either a DPX 400 (Bruker), an AMX 400 (Bruker) or an Ascend 400 Avance III HD (Bruker). The spectra were calibrated using the residual solvent peak: $\delta(CDCl_3)$ = 7.26 ppm, $\delta(C_6D_6)$ = 7.16 ppm. Chemical shift δ is given in parts per million (ppm), coupling constant J in hertz (Hz) and multiplicity as follows: s, singlet; d, doublet; t, triplet; q, quadruplet; p, pentet; sex, sextet; sep, septet; m, multiplet; m_c, centered multiplet; brs, broad signal; or combination of these acronyms. NMR spectra were processed using TopSpin (Bruker).

^{13}C-NMR experiments were recorded in $CDCl_3$ or C_6D_6 using either a DPX 400 (Bruker), an AMX 400 (Bruker) or an Ascend 400 Avance III HD (Bruker). The spectra were calibrated using the residual solvent peak: $\delta(CDCl_3)$ = 77.16 ppm, $\delta(C_6D_6)$ = 128.06 ppm. Chemical shift δ is given in parts per million (ppm). NMR spectra were processed using TopSpin (Bruker).

High Resolution Mass Spectra (HRMS) were obtained either using a Q-Tof Premier (Waters), a LCT Premier (Waters) or a GC-system Agilent 6890 coupled with an Agilent 5973. Both the masses found and the masses calculated are given.

Optical rotation $[\alpha]_D^{20}$ were measured either on a P3000 polarimeter (A. Krüss Optronic, λ = 589 nm) or a Perkin-Elmer 341 (λ = 589 nm). The sample concentration (in g/100 mL) is given with every single experiment.

2. Experimental Procedures and Characterization Data

2-1. Synthesis of TIB esters

General Procedure 1 (GP1): Mitsunobu Conditions

The required primary alcohol (1.1 equiv) was dissolved in anhydrous THF (0.3 M), PPh_3 (1.0 equiv) and TIBOH (1.0 equiv) were added successively. After cooling to 0 °C, DIAD (1.1 equiv, 0.12 mL/min) was added, the reaction mixture was slowly warmed to rt and stirred overnight at that temperature. MTBE and sat. aq. $NaHCO_3$ were added and the phases separated. The aqueous phase was extracted with MTBE (3x), the organic layers combined and dried over Na_2SO_4. The crude material was loaded on silica and purified by flash column chromatography.

General Procedure 2 (GP2): Phase transfer system

Following Beak's procedure,[1] a solution of TIBOH (1.0 equiv) in $CHCl_3$ (0.2 M) was treated with a solution of NaOH (3.1 equiv) and *n*Bu_4NHSO_4 (8 mol%) in H_2O (0.8 M). After addition of the required alkyl halide (5.0 equiv) the biphasic reaction mixture was stirred at rt. The phases were separated and the aqueous phase was extracted with CH_2Cl_2 (3x). The combined organic phases were dried over Na_2SO_4 and concentrated *in vacuo*. The resulting oil was filtered through a short plug of silica using PE:EtOAc (9:1) as eluent. The solvent was removed under reduced pressure to afford the corresponding TIB ester.

TIB ester 19

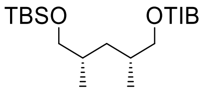

19
$C_{29}H_{52}O_3Si$
476.82

Using GP1, literature known primary alcohol (750 mg, 3.04 mmol, 1.1 equiv),[2] DIAD (0.60 mL, 3.04 mmol, 1.1 equiv), PPh_3 (725 mg, 2.77 mmol, 1.0 equiv) and TIBOH (687 mg, 2.77 mmol, 1.0 equiv) gave TIB ester **19** (1.06 g, 2.22 mmol, 80%) after purification by flash column chromatography (PE:EtOAc 99:1) as a colorless oil.

^{1}H-NMR (400 MHz, C_6D_6): δ = 7.10 (s, 2H), 4.30 (dd, *J* = 10.8, 4.9 Hz, 1H), 4.07 (dd, *J* = 10.8, 6.8 Hz, 1H), 3.40 (dd, *J* = 9.7, 5.0 Hz, 1H), 3.28 (dd, *J* = 9.7, 6.3 Hz, 1H), 3.17 (sep, *J* = 6.8 Hz, 2H), 2.77 (sep, *J* = 6.8 Hz, 1H), 1.95 (m_c, 1H), 1.70 (m_c, 1H), 1.47 (m_c, 1H), 1.31 (d, *J* = 6.8 Hz, 12H), 1.21 (d, *J* = 6.8 Hz, 6H), 0.97 (s, 9H), 0.95-0.91 (m, 7H), 0.05 (s, 6H) ppm;

^{13}C{^{1}H}-NMR (101 MHz, C_6D_6): δ = 170.8, 150.3, 145.4, 132.0, 121.2, 70.0, 68.2, 37.7, 34.9, 33.3, 32.1, 30.5, 26.1, 24.4, 24.2, 18.5, 18.0, 17.8, −5.2, −5.3 ppm;

HRMS (ESI) m/z: calcd for $C_{29}H_{52}O_3SiNa$ $[M+Na]^+$ 499.3583, found: 499.3582;

R*f* = 0.6 (PE:EtOAc 95:5, uv, vanillin);

$[\alpha]_D^{20}$ = −7.63 (*c* 0.9, MeOH).

TIB ester 61

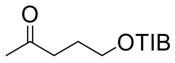

61
$C_{21}H_{32}O_3$
332.48

Using GP1, 5-hydroxy-2-pentanone (**60**) (1.21 g, 11.8 mmol, 1.1 equiv), DIAD (2.3 mL, 11.8 mmol, 1.1 equiv), PPh_3 (2.81 g, 10.7 mmol, 1.0 equiv) and TIBOH (2.66 g, 10.7 mmol, 1.0 equiv) gave TIB ester **61** (2.68 g, 8.06 mmol, 75%) after purification by flash column chromatography (PE:MTBE 9:1) as a colorless oil.

^{1}H-NMR (400 MHz, $CDCl_3$): δ = 7.01 (s, 2H), 4.31 (t, *J* = 6.4 Hz, 2H), 2.94-2.77 (m, 3H), 2.58 (t, *J* = 7.2 Hz, 2H), 2.14 (s, 3H), 2.04-1.97 (m, 2H), 1.24 (d, *J* = 6.8 Hz, 18H) ppm;

^{13}C{^{1}H}-NMR (101 MHz, $CDCl_3$): δ = 207.6, 171.0, 150.3, 144.8, 130.5, 121.0, 64.1, 40.0, 34.6, 31.7, 30.1, 24.3, 24.1, 22.9 ppm;

HRMS (ESI) m/z: calcd for $C_{21}H_{32}O_3Na$ $[M+Na]^+$ 355.2249, found: 355.2243;

R_f = 0.3 (PE:MTBE 9:1, uv, vanillin).

TIB ester 59

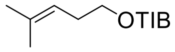

59
$C_{22}H_{34}O_2$
330.51

Using GP2, TIBOH (1.50 g, 6.04 mmol, 1.0 equiv), 1-bromo-4-methylpent-3-ene (**63**) (4.0 mL, 30.2 mmol, 5.0 equiv), NaOH (0.75 g, 18.7 mmol, 3.1 equiv) and *n*Bu_4NHSO_4 (0.16 g, 0.48 mmol, 8 mol%) gave TIB ester **59** (1.95 g, 5.90 mmol, ≥ 95%) after purification by short flash column chromatography (PE:EtOAc 9:1) as a colorless oil.

^{1}H-NMR (400 MHz, $CDCl_3$): δ = 7.00 (s, 2H), 5.16 (m_c, 1H), 4.29 (t, *J* = 6.9 Hz, 2H), 2.94-2.81 (m, 3H), 2.43 (m_c, 2H), 1.71 (d, *J* = 1.1 Hz, 3H), 1.64 (s, 3H), 1.24 (d, *J* = 6.9 Hz, 18H) ppm;

^{13}C{^{1}H}-NMR (101 MHz, $CDCl_3$): δ = 171.1, 150.2, 144.9, 134.7, 130.8, 121.0, 119.7, 64.9, 34.6, 31.6, 27.8, 25.9, 24.3, 24.1, 18.0 ppm;

HRMS (ESI) m/z: calcd for $C_{22}H_{34}O_2Na$ $[M+Na]^+$ 353.2457, found: 353.2463;

R_f = 0.7 (PE:EtOAc 95:5, uv, $KMnO_4$).

2-2. Synthesis of carbamates

General Procedure 3 (GP3): CbCl

The required primary alcohol (1.0 equiv) was dissolved in $C_2H_4Cl_2$ (0.3 M) and Et_3N (1.5 equiv) and *N*,*N*-di*iso*propylcarbamoyl chloride (1.5 equiv) were added successively. After heating to 70 °C overnight H_2O was added. The phases were separated, and the aqueous phase was extracted with CH_2Cl_2 (3x), the organic layers were combined and dried over Na_2SO_4. The solvent was removed *in vacuo* and the crude material was purified by flash column chromatography to afford the corresponding carbamate.

Carbamate 22

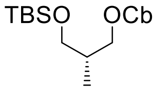

22

$C_{17}H_{37}NO_3Si$

331.57

Using GP3, literature known primary alcohol (1.20 g, 5.87 mmol, 1.0 equiv),[3] CbCl (1.44 g, 8.81 mmol, 1.5 equiv) and Et_3N (1.2 mL, 8.81 mmol, 1.5 equiv) gave carbamate **22** (1.43 g, 4.31 mmol, 73%) after purification by flash column chromatography (PE:MTBE 95:5) as a colorless oil.

^{1}H-NMR (400 MHz, $CDCl_3$): δ = 4.22-3.69 (m, 4H), 3.58 (dd, *J* = 9.9, 5.9 Hz, 1H), 3.52 (dd, *J* = 9.9, 5.9 Hz, 1H), 1.99 (m_c, 1H), 1.20 (d, *J* = 6.8 Hz, 12H), 0.96 (d, *J* = 6.8 Hz, 3H), 0.89 (s, 9H), 0.03 (s, 6H) ppm;

^{13}C{^{1}H}-NMR (101 MHz, $CDCl_3$): δ = 156.0, 66.7, 65.3, 45.9 (brs), 35.9, 26.1, 21.2 (brs), 18.5, 14.2, −5.31, −5.32 ppm;

HRMS (ESI) m/z: calcd for $C_{17}H_{37}NO_3SiNa$ $[M+Na]^+$ 354.2440, found: 354.2437;

R*f* = 0.2 (PE:MTBE 95:5, $KMnO_4$);

$[\alpha]_D^{20}$ = +1.10 (*c* 0.7, $CHCl_3$).

2-3. 1,2-Metallate rearrangement of vinyl boronates

General Procedure 4 (GP4): TIB esters

To a stirred solution of TIB ester (1.5 equiv) and diamine (1.5 equiv) in Et_2O (0.2 M) at −78 °C was added *s*BuLi (1.3 M in hexanes, 1.4 equiv). The reaction mixture was stirred for 5 h at that temperature before a solution of vinyl boronic ester (1.0 equiv) in Et_2O (0.5 M) was added. After stirring for further 3 h at −78 °C, the reaction mixture was warmed to 45 °C and stirred overnight. The reaction mixture was cooled to rt, sat. aq. NH_4Cl was added, and the biphasic mixture was stirred for 15 min. The phases were separated, the organic layer was washed with sat. aq. NH_4Cl (3x) and the combined aqueous phases were extracted with MTBE (3x). The combined organic phases were dried over Na_2SO_4, concentrated *in vacuo* and the crude material was purified by a short flash column chromatography (to remove TIBOH).

The residue was dissolved in THF (0.2 M) and cooled to −20 °C. A premixed, ice-cooled solution of NaOH (2.0 M)/H_2O_2 (35%, 2/1 v/v, 0.12 M) was added dropwise. The reaction mixture was stirred for at rt before being quenched by the slow addition of sat. aq. $Na_2S_2O_3$ at 0 °C after TLC showed full conversion. The solution was diluted with MTBE, the phases were separated, and the aqueous phase was extracted with MTBE (3x). The combined organic layers were dried over Na_2SO_4 and concentrated *in vacuo*. The crude product was purified by flash column chromatography to afford allylic alcohol.

The stereochemistry is assigned by the usual induction of (+)-sparteine and (–)-sparteine[4,5] due to no exerted substrate induction[6,7] of the used TIB esters.

General Procedure 5 (GP5): Carbamates

To a stirred solution of carbamate (1.5 equiv) and diamine (1.5 equiv) in Et_2O (0.2 M) at −78 °C was added *s*BuLi (1.3 M in hexanes, 1.4 equiv). The reaction mixture was stirred for 5 h at that temperature before a solution of vinyl boronic ester (1.0 equiv) in Et_2O (0.5 M) was added. The reaction mixture was stirred for 3 h at −78 °C.
In parallel, magnesium turnings were activated (2x 1 M HCl, 2x H_2O, 2x acetone, drying under high vacuum). The required amount (2.0 equiv) was dissolved in Et_2O (0.8 M) and 1,2-dibromoethane (2.0 equiv) was added under water bath cooling. The reaction mixture was stirred for 2 h at this temperature.
The biphasic $MgBr_2·OEt_2$ solution was added dropwise to the main reaction mixture, which was then stirred for another 30 min at −78 °C before being warmed to 45 °C and stirred overnight. The reaction mixture was cooled to rt, sat. aq. NH_4Cl was added, and the biphasic mixture was stirred for 15 min. The phases were separated, the organic layer was washed with sat. aq. NH_4Cl (3x) and the combined aqueous phases were extracted with MTBE (3x). The combined organic phases were dried over Na_2SO_4 and concentrated *in vacuo* and the crude material was purified by a short flash column chromatography (to remove excess of the carbamate).
The residue was dissolved in THF (0.2 M) and cooled to −20 °C. A premixed, ice-cooled solution of NaOH (2.0 M)/H_2O_2 (35%, 2/1 v/v, 0.12 M) was added dropwise. The reaction mixture was stirred at rt before being quenched by the slow addition of sat. aq. $Na_2S_2O_3$ at 0 °C after TLC showed full conversion. The solution was diluted with MTBE, the phases were separated, and the aqueous phase was extracted with MTBE (3x). The combined organic layers were dried over Na_2SO_4 and concentrated *in vacuo*. The crude product was purified by flash column chromatography to afford allylic alcohol.
The stereochemistry is assigned by the usual induction of (+)-sparteine and (–)-sparteine[4,5] due to no exerted substrate induction[6,7] of the used carbamates.

General Procedure 6 (GP6): TIB esters and TBS-protection

To a stirred solution of TIB ester (1.5 equiv) and diamine (1.5 equiv) in Et_2O (0.2 M) at −78 °C was added *s*BuLi (1.3 M in hexanes, 1.4 equiv). The reaction mixture was stirred for 5 h at that temperature before a solution of vinyl boronic ester (1.0 equiv) in Et_2O (0.5 M) was added. After stirring for further 3 h at −78 °C, the reaction mixture was warmed to 45 °C and stirred overnight. The reaction mixture was cooled to rt, sat. aq. NH_4Cl was added, and the biphasic

mixture was stirred for 15 min. The phases were separated, the organic layer was washed with sat. aq. NH_4Cl (3x) and the combined aqueous phases were extracted with MTBE (3x). The combined organic phases were dried over Na_2SO_4, concentrated *in vacuo* and the crude material was purified by a short flash column chromatography (to remove TIBOH).
The residue was dissolved in THF (0.2 M) and cooled to −20 °C. A premixed, ice-cooled solution of NaOH (2.0 M)/H_2O_2 (35%, 2/1 v/v, 0.12 M) was added dropwise. The reaction mixture was stirred for at rt before being quenched by the slow addition of sat. aq. $Na_2S_2O_3$ at 0 °C after TLC showed full conversion. The solution was diluted with MTBE, the phases were separated, and the aqueous phase was extracted with MTBE (3x). The combined organic layers were dried over Na_2SO_4 and concentrated *in vacuo*. The crude product was purified by flash column chromatography to afford allylic alcohol.
To a solution of the obtained allylic alcohol in CH_2Cl_2 (1.0 M) at −78 °C were added 2,6-lutidine (4.0 equiv) and TBSOTf (2.0 equiv) successively. The reaction mixture was stirred for 20 min at −78 °C and at 0 °C until TLC showed full consumption. After the addition of sat. aq. $NaHCO_3$ the organic layer was separated, and the aqueous layer was extracted with CH_2Cl_2 (3x). The combined organic layers were washed with aq. $KHSO_4$ (1.0 M) and sat. aq. NaCl, dried over Na_2SO_4 and concentrated *in vacuo*. The crude material was purified by flash column chromatography to afford the TBS-protected allylic alcohol.
The stereochemistry is assigned by the usual induction of (+)-sparteine and (–)-sparteine[4,5] due to no exerted substrate induction[6,7] of the used TIB esters.

(*R*)-Allylic alcohol 36a

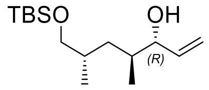

36a
$C_{15}H_{32}O_2Si$
272.50

According to GP4, TIB ester **18** (211 mg, 0.44 mmol, 1.5 equiv),[8] vinyl boronic ester **23** (45.4 mg, 0.29 mmol, 1.0 equiv), *s*BuLi (0.31 mL, 0.41 mmol, 1.4 equiv) and (+)-sparteine (0.10 mL, 0.44 mmol, 1.5 equiv) gave (*R*)-allylic alcohol **36a** (40.0 mg, 0.15 mmol, 52% o2s, dr ≥ 19:1) after purification by flash column chromatography (PE:MTBE 8:1) as a colorless oil.

^{1}H-NMR (400 MHz, C_6D_6): δ = 5.73 (m_c, 1H), 5.06 (m_c, 2H), 3.72 (m_c, 1H), 3.37 (m_c, 2H), 1.77-1.58 (m, 2H), 1.26 (m_c, 2H), 1.04 (brs, 1H), 1.00 (s, 9H), 0.88-0.85 (m, 6H), 0.07 (s, 6H) ppm;

^{1}H-NMR (400 MHz, $CDCl_3$): δ = 5.86 (m_c, 1H), 5.19 (m_c, 2H), 3.93 (m_c, 1H), 3.40 (m_c, 2H), 1.77-1.64 (m, 3H), 1.26-1.14 (m, 2H), 0.89 (s, 9H), 0.87 (d, J = 6.8 Hz, 3H), 0.85 (d, J = 6.6 Hz, 3H), 0.04 (s, 6H) ppm;

^{13}C{^{1}H}-NMR (101 MHz, $CDCl_3$): δ = 139.4, 115.9, 77.9, 69.3, 36.0, 35.8, 33.2, 26.1, 18.5, 16.5, 15.1, −5.2 ppm;

HRMS (ESI) m/z: calcd for $C_{15}H_{33}O_2Si$ $[M+H]^+$ 273.2250, found: 273.2260;

R_f = 0.5 (PE:MTBE 4:1, vanillin);

$[\alpha]_D^{20}$ = −10.0 (c 0.7, $CHCl_3$).

Analytical data are in accordance with the literature.[6]

(*S*)-Allylic alcohol 36b

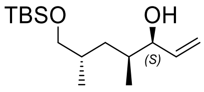

36b

$C_{15}H_{32}O_2Si$

272.50

According to GP4, TIB ester **18** (211 mg, 0.44 mmol, 1.5 equiv),[8] vinyl boronic ester **23** (45.4 mg, 0.29 mmol, 1.0 equiv), *s*BuLi (0.31 mL, 0.41 mmol, 1.4 equiv) and (−)-sparteine (0.10 mL, 0.44 mmol, 1.5 equiv) gave (*S*)-allylic alcohol **36b** (52.0 mg, 0.19 mmol, 66% o2s, dr ≥ 19:1) after purification by flash column chromatography (PE:MTBE 8:1) as a colorless oil.

^{1}H-NMR (400 MHz, C_6D_6): δ = 5.74 (m_c, 1H), 5.07 (m_c, 2H), 3.77 (brs, 1H), 3.37 (m_c, 2H), 1.78-1.57 (m, 2H), 1.25 (m_c, 2H), 0.99 (brs, 10H), 0.88 (d, J = 6.7 Hz, 6H), 0.06 (s, 6H) ppm;

^{13}C{^{1}H}-NMR (101 MHz, C_6D_6): δ = 140.5, 114.7, 77.1, 69.4, 36.5, 36.1, 33.6, 26.2, 18.6, 16.6, 14.6, −5.18, −5.19 ppm;

HRMS (ESI) m/z: calcd for $C_{15}H_{33}O_2Si$ $[M+H]^+$ 273.2250, found: 273.2256;

R_f = 0.5 (PE:MTBE 4:1, vanillin);

$[\alpha]_D^{20}$ = −32.7 (c 1.0, $CHCl_3$).

Analytical data are in accordance with the literature.[6]

Allylic alcohol 36

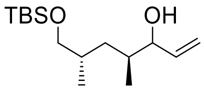

36

$C_{15}H_{32}O_2Si$

272.50

According to GP4, TIB ester **18** (211 mg, 0.44 mmol, 1.5 equiv),[8] vinyl boronic ester **23** (45.4 mg, 0.29 mmol, 1.0 equiv), *s*BuLi (0.31 mL, 0.41 mmol, 1.4 equiv) and TMEDA (0.07 mL, 0.44 mmol, 1.5 equiv) gave allylic alcohol **36** (70.0 mg, 0.26 mmol, 90% o2s, dr 1:1) after purification by flash column chromatography (PE:MTBE 8:1) as a colorless oil.

^{1}H-NMR (400 MHz, C_6D_6): δ = 5.78-5.69 (m, 1H), 5.15-4.99 (m, 2H), 3.79-3.70 (m, 1H), 3.42-3.32 (m, 2H), 1.78-1.57 (m, 2H), 1.31-1.19 (m, 2H), 1.05 (brs, 0.5H), 0.99 (brs, 9.5H), 0.89-0.85 (m, 6H), 0.07-0.06 (m, 6H) ppm;

Asterisk marks carbons of allylic alcohol **36b**:

^{13}C{^{1}H}-NMR (101 MHz, C_6D_6): δ = 140.5*, 140.1, 115.1, 114.7*, 77.6, 77.1*, 69.42, 69.36*, 36.5*, 36.3, 36.1*, 35.9, 33.6*, 33.5, 26.2, 18.6, 16.6*, 16.5, 15.3, 14.6*, −5.17, −5.19 ppm;

HRMS (ESI) m/z: calcd for $C_{15}H_{32}O_2SiNa$ $[M+Na]^+$ 295.2069, found: 295.2070;

R*f* = 0.5 (PE:MTBE 4:1, vanillin);

Analytical data are in accordance with the literature.[6]

(*R*)-Allylic alcohol 37

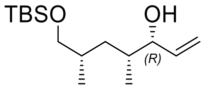

37
$C_{15}H_{32}O_2Si$
272.50

According to GP4, TIB ester **19** (211 mg, 0.44 mmol, 1.5 equiv), vinyl boronic ester **23** (45.4 mg, 0.29 mmol, 1.0 equiv), *s*BuLi (0.31 mL, 0.41 mmol, 1.4 equiv) and (+)-sparteine (0.10 mL, 0.44 mmol, 1.5 equiv) gave (*R*)-allylic alcohol **37** (54.0 mg, 0.20 mmol, 69% o2s, dr ≥ 19:1) after purification by flash column chromatography (PE:MTBE 8:1) as a colorless oil.

^{1}H-NMR (400 MHz, $CDCl_3$): δ = 5.87 (m_c, 1H), 5.19 (m_c, 2H), 4.01 (m_c, 1H), 3.47 (dd, *J* = 9.7, 5.3 Hz, 1H), 3.37 (dd, *J* = 9.7, 6.3 Hz, 1H), 1.75-1.65 (m, 2H), 1.59-1.49 (m, 2H), 0.96-0.89 (m, 16H), 0.04 (s, 6H) ppm;

^{13}C{^{1}H}-NMR (101 MHz, $CDCl_3$): δ = 140.0, 115.2, 76.3, 68.1, 36.8, 36.1, 33.3, 26.1, 18.5, 18.3, 15.1, −5.235, −5.239 ppm;

HRMS (ESI) m/z: calcd for $C_{15}H_{32}O_2SiNa$ $[M+Na]^+$ 295.2069, found: 295.2070;

R*f* = 0.5 (PE:MTBE 4:1, vanillin);

$[\alpha]_D^{20}$ = +30.0 (*c* 0.6, $CHCl_3$).

(*S*)-Allylic alcohol 38

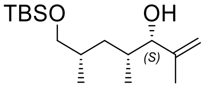

38
$C_{16}H_{34}O_2Si$
286.53

According to GP4, TIB ester **19** (228 mg, 0.48 mmol, 1.5 equiv), vinyl boronic ester **24** (53.6 mg, 0.32 mmol, 1.0 equiv), *s*BuLi (0.34 mL, 0.45 mmol, 1.4 equiv) and (+)-sparteine (0.11 mL, 0.48 mmol, 1.5 equiv) gave (*S*)-allylic alcohol **38** (70.1 mg, 0.24 mmol, 75% o2s, dr ≥ 19:1) after purification by flash column chromatography (PE:MTBE 95:5) as a colorless oil.

^{1}H-NMR (400 MHz, $CDCl_3$): δ = 4.92 (m_c, 2H), 3.87 (brs, 1H), 3.47 (dd, J = 9.7, 5.2 Hz, 1H), 3.38 (dd, J = 9.7, 6.2 Hz, 1H), 1.79-1.67 (m, 5H), 1.49-1.42 (m, 2H), 0.98-0.86 (m, 16H), 0.04 (s, 6H) ppm;

^{13}C{^{1}H}-NMR (101 MHz, $CDCl_3$): δ = 146.9, 111.2, 78.6, 68.0, 37.5, 33.1, 33.0, 26.1, 18.8, 18.5, 18.2, 14.3, −5.2 ppm;

HRMS (ESI) m/z: calcd for $C_{16}H_{34}O_2SiNa$ $[M+Na]^+$ 309.2226, found: 309.2233;

R$_f$ = 0.3 (PE:MTBE 95:5, vanillin);

$[\alpha]_D^{20}$ = +12.9 (*c* 0.8, $CHCl_3$).

(*S*)-Allylic alcohol 39

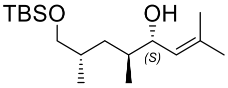

39
$C_{17}H_{36}O_2Si$
300.56

According to GP4, TIB ester **18** (244 mg, 0.51 mmol, 1.5 equiv),[8] vinyl boronic ester **25** (62.2 mg, 0.34 mmol, 1.0 equiv), *s*BuLi (0.37 mL, 0.48 mmol, 1.4 equiv) and (+)-sparteine (0.12 mL, 0.51 mmol, 1.5 equiv) gave (*S*)-allylic alcohol **39** (79.7 mg, 0.27 mmol, 79% o2s, dr ≥ 19:1) after purification by flash column chromatography (PE:MTBE 95:5) as a colorless oil.

^{1}H-NMR (400 MHz, $CDCl_3$): δ = 5.19 (m_c, 1H), 4.12 (m_c, 1H), 3.39 (dd, J = 6.4, 1.1 Hz, 2H), 1.74 (d, J = 1.3 Hz, 3H), 1.72-1.64 (m, 5H), 1.49 (brs, 1H), 1.20 (m_c, 2H), 0.89 (s, 9H), 0.83 (m_c, 6H), 0.04 (s, 6H) ppm;

^{13}C{^{1}H}-NMR (101 MHz, $CDCl_3$): δ = 135.9, 126.2, 73.1, 69.4, 36.8, 36.2, 33.1, 26.11, 26.10, 18.6, 18.5, 16.4, 14.7, −5.189, −5.194 ppm;

HRMS (ESI) m/z: calcd for $C_{17}H_{36}O_2SiNa$ $[M+Na]^+$ 323.2382, found: 323.2377;

R$_f$ = 0.3 (PE:MTBE 8:1, vanillin);

$[\alpha]_D^{20}$ = −10.0 (c 0.5, $CHCl_3$).

(*S*)-Allylic alcohol 40

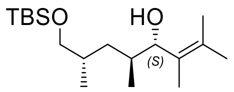

40
$C_{18}H_{38}O_2Si$
314.59

According to GP4, TIB ester **18** (227 mg, 0.48 mmol, 1.5 equiv),[8] vinyl boronic ester **26** (62.2 mg, 0.32 mmol, 1.0 equiv), *s*BuLi (0.34 mL, 0.44 mmol, 1.4 equiv) and (+)-sparteine (0.11 mL, 0.48 mmol, 1.5 equiv) gave (*S*)-allylic alcohol **40** (50.3 mg, 0.16 mmol, 50% o2s, dr ≥ 19:1) after purification by flash column chromatography (PE:MTBE 95:5) as a colorless oil.

^{1}H-NMR (400 MHz, $CDCl_3$): δ = 4.23 (d, J = 9.6 Hz, 1H), 3.42 (d, J = 6.5 Hz, 2H), 1.80-1.68 (m, 8H), 1.613-1.607 (m, 4H), 1.49 (m$_c$, 1H), 1.22 (m$_c$, 1H), 0.90 (s, 9H), 0.87 (d, J = 6.7 Hz, 3H), 0.66 (d, J = 6.8 Hz, 3H), 0.05 (s, 6H) ppm;

^{13}C{^{1}H}-NMR (101 MHz, $CDCl_3$): δ = 129.1, 128.3, 76.1, 69.6, 37.1, 34.4, 33.4, 26.2, 21.2, 20.2, 18.6, 16.5, 15.7, 12.1, −5.2 ppm;

HRMS (ESI) m/z: calcd for $C_{18}H_{38}O_2SiNa$ $[M+Na]^+$ 337.2539, found: 337.2544;

R$_f$ = 0.3 (PE:MTBE 95:5, vanillin);

$[\alpha]_D^{20}$ = −11.9 (c 0.7, $CHCl_3$).

(*S*)-Allylic alcohol 41

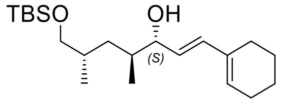

41
$C_{21}H_{40}O_2Si$
352.63

According to GP4, TIB ester **18** (211 mg, 0.44 mmol, 1.5 equiv),[8] vinyl boronic ester **27** (67.4 mg, 0.29 mmol, 1.0 equiv), *s*BuLi (0.31 mL, 0.41 mmol, 1.4 equiv) and (+)-sparteine (0.10 mL, 0.44 mmol, 1.5 equiv) gave (*S*)-allylic alcohol **41** (60.0 mg, 0.17 mmol, 59% o2s, dr ≥ 19:1) after purification by flash column chromatography (PE:MTBE 93:7) as a colorless oil.

^{1}H-NMR (400 MHz, C_6D_6): δ = 6.21 (d, J = 15.8 Hz, 1H), 5.68 (m$_c$, 1H), 5.58 (m$_c$, 1H), 3.86 (brs, 1H), 3.42 (dd, J = 9.6, 6.1 Hz, 1H), 3.36 (dd, J = 9.6, 6.4 Hz, 1H), 2.09-2.06 (m, 2H), 2.01-1.98 (m, 2H), 1.81-1.71 (m, 2H), 1.56-1.50 (m, 2H), 1.48-1.42 (m, 2H), 1.41-1.28 (m, 2H), 1.08 (brs, 1H), 1.00 (s, 9H), 0.93 (d, J = 6.8 Hz, 3H), 0.91 (d, J = 6.6 Hz, 3H), 0.07 (s, 6H) ppm;

^{13}C{^{1}H}-NMR (101 MHz, C_6D_6): δ = 135.6, 135.1, 129.3, 127.4, 77.8, 69.5, 36.9, 36.4, 33.6, 26.2, 26.1, 25.0, 22.9, 22.8, 18.6, 16.5, 15.4, −5.2 ppm;

HRMS (ESI) m/z: calcd for $C_{21}H_{40}O_2SiNa$ $[M+Na]^+$ 375.2695, found: 375.2685;

R$_f$ = 0.3 (PE:MTBE 93:7, vanillin);

$[\alpha]_D^{20}$ = −6.31 (*c* 0.8, $CHCl_3$).

TBS-ether 42

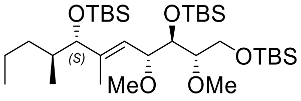

42

$C_{33}H_{72}O_5Si_3$

633.19

According to GP6, literature known TIB ester **20** (46.0 mg, 138 µmol, 1.5 equiv),[9] vinyl boronic ester **8** (50.0 mg, 91.8 µmol, 1.0 equiv),[6] *s*BuLi (99 µL, 129 µmol, 1.4 equiv), (+)-sparteine (32 µL, 138 µmol, 1.5 equiv), 2,6-lutidine (43 µL, 367 µmol, 4.0 equiv) and TBSOTf (42 µL, 184 µmol, 2.0 equiv) gave TBS-ether **42** (26.1 mg, 41.2 µmol, 45% o3s, dr ≥ 19:1) after purification by flash column chromatography (PE:MTBE 99:1) as a colorless oil.

^{1}H-NMR (400 MHz, C_6D_6): δ = 5.70 (d, *J* = 9.6 Hz, 1H), 4.37 (dd, *J* = 9.6, 1.8 Hz, 1H), 4.22 (dd, *J* = 8.5, 1.8 Hz, 1H), 4.01 (dd, *J* = 11.2, 2.0 Hz, 1H), 3.89-3.80 (m, 2H), 3.32 (s, 3H), 3.22 (s, 3H), 3.14 (m_c, 1H), 1.82-1.67 (m, 5H), 1.50-1.11 (m, 3H), 1.08 (s, 9H), 1.04 (s, 9H), 1.03 (s, 9H), 0.97 (t, *J* = 7.2 Hz, 3H), 0.86 (d, *J* = 6.8 Hz, 3H), 0.35 (s, 3H), 0.28 (s, 3H), 0.15-0.14 (m, 12H) ppm;

^{13}C{^{1}H}-NMR (101 MHz, C_6D_6): δ = 142.4, 124.1, 83.8, 82.9, 78.2, 74.3, 62.5, 57.8, 56.0, 37.4, 35.1, 26.5, 26.24, 26.21, 20.6, 18.9, 18.6, 18.5, 16.6, 14.8, 12.5, −3.5, −4.0, −4.7, −4.8, −5.0, −5.1 ppm;

HRMS (ESI) m/z: calcd for $C_{33}H_{72}O_5Si_3Na$ $[M+Na]^+$ 655.4585, found: 655.4588;

R$_f$ = 0.3 (PE:MTBE 99:1, vanillin);

$[\alpha]_D^{20}$ = −32.8 (*c* 1.2, $CHCl_3$).

TBS-ether 43

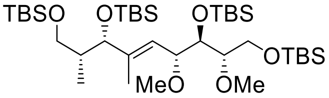

43

$C_{37}H_{82}O_6Si_4$

735.40

According to GP6, literature known TIB ester **21** (60.0 mg, 138 µmol, 1.5 equiv),[10] vinyl boronic ester **8** (50.0 mg, 91.8 µmol, 1.0 equiv),[6] *s*BuLi (99 µL, 129 µmol, 1.4 equiv), (+)-

sparteine (32 µL, 138 µmol, 1.5 equiv), 2,6-lutidine (43 µL, 367 µmol, 4.0 equiv) and TBSOTf (42 µL, 184 µmol, 2.0 equiv) gave TBS-ether **43** (34.8 mg, 47.3 µmol, 52% o3s, dr ≥ 19:1) after purification by flash column chromatography (PE:MTBE 99:1) as a colorless oil.

^{1}H-NMR (400 MHz, C_6D_6): δ = 5.82 (m$_c$, 1H), 4.38-4.31 (m, 2H), 4.25 (dd, *J* = 8.9, 2.0 Hz, 1H), 4.05 (dd, *J* = 11.2, 2.0 Hz, 1H), 3.90 (dd, *J* = 11.2, 4.7 Hz, 1H), 3.63 (dd, *J* = 9.8, 7.2 Hz, 1H), 3.43 (dd, *J* = 9.8, 6.2 Hz, 1H), 3.32 (s, 3H), 3.17 (s, 3H), 3.16-3.13 (m, 1H), 1.91-1.84 (m, 1H), 1.71 (d, *J* = 1.2 Hz, 3H), 1.09 (s, 9H), 1.06 (s, 9H), 1.04-1.00 (m, 21H), 0.35 (s, 3H), 0.30 (s, 3H), 0.18 (s, 3H), 0.16 (s, 6H), 0.14 (s, 3H), 0.11 (s, 6H) ppm;

^{13}C{^{1}H}-NMR (101 MHz, C_6D_6): δ = 142.9, 121.9, 83.0, 77.8, 76.9, 74.2, 65.6, 62.5, 57.9, 55.6, 40.8, 26.7, 26.4, 26.23, 26.20, 19.0, 18.62, 18.56, 18.49, 14.1, 11.3, −3.4, −3.8, −4.79, −4.85, −4.96, −5.01, −5.079, −5.085 ppm;

HRMS (ESI) m/z: calcd for $C_{37}H_{82}O_6Si_4Na$ $[M+Na]^+$ 757.5086, found: 757.5050;

R*f* = 0.3 (PE:MTBE 99:1, vanillin);

$[\alpha]_D^{20}$ = −30.7 (*c* 2.4, $CHCl_3$).

(*S*)-Allylic alcohol 44

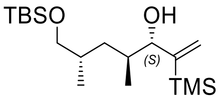

44

$C_{18}H_{40}O_2Si_2$

344.69

According to GP4, TIB ester **18** (237 mg, 0.50 mmol, 1.5 equiv),[8] vinyl boronic ester **28** (75.0 mg, 0.33 mmol, 1.0 equiv), *s*BuLi (0.36 mL, 0.46 mmol, 1.4 equiv) and (+)-sparteine (0.12 mL, 0.50 mmol, 1.5 equiv) gave (*S*)-allylic alcohol **44** (82.1 mg, 0.24 mmol, 73% o2s, dr ≥ 19:1) after purification by flash column chromatography (PE:MTBE 93:7) as a colorless oil.

^{1}H-NMR (400 MHz, C_6D_6): δ = 5.66 (dd, *J* = 2.8, 1.3 Hz, 1H), 5.42 (dd, *J* = 2.8, 1.0 Hz, 1H), 3.88 (brs, 1H), 3.44 (dd, *J* = 9.6, 6.2 Hz, 1H), 3.39 (dd, *J* = 9.6, 6.3 Hz, 1H), 1.83-1.71 (m, 2H), 1.49 (m$_c$, 1H), 1.32 (m$_c$, 1H), 1.15 (brs, 1H), 1.00 (s, 9H), 0.92 (d, *J* = 6.6 Hz, 3H), 0.88 (d, *J* = 6.7 Hz, 3H), 0.18 (s, 9H), 0.08 (s, 6H) ppm;

^{13}C{^{1}H}-NMR (101 MHz, C_6D_6): δ = 154.9, 125.2, 82.5, 69.7, 35.1, 34.5, 33.6, 26.2, 18.6, 17.3, 16.4, −0.02, −5.16, −5.17 ppm;

HRMS (ESI) m/z: calcd for $C_{18}H_{40}O_2Si_2Na$ $[M+Na]^+$ 367.2465, found: 367.2463;

R*f* = 0.3 (PE:MTBE 93:7, vanillin);

$[\alpha]_D^{20}$ = −16.4 (*c* 0.6, $CHCl_3$).

(*S*)-Allylic alcohol 45

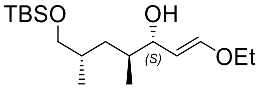

45
$C_{17}H_{36}O_3Si$
316.56

According to GP4, TIB ester **18** (236 mg, 0.50 mmol, 1.5 equiv),[8] vinyl boronic ester **29** (65.5 mg, 0.33 mmol, 1.0 equiv), *s*BuLi (0.36 mL, 0.46 mmol, 1.4 equiv) and (+)-sparteine (0.12 mL, 0.50 mmol, 1.5 equiv) gave (*S*)-allylic alcohol **45** (66.0 mg, 0.21 mmol, 64% o2s, dr ≥ 19:1) after purification by flash column chromatography (PE:MTBE 4:1 + 1% Et_3N) as a colorless oil.

^{1}H-NMR (400 MHz, C_6D_6): δ = 6.31 (d, J = 12.7 Hz, 1H), 4.77 (m_c, 1H), 3.67 (brs, 1H), 3.46-3.35 (m, 4H), 1.82-1.70 (m, 2H), 1.32 (m_c, 2H), 1.04-1.00 (m, 12H), 0.96 (brs, 1H), 0.93 (d, J = 6.6 Hz, 6H), 0.08 (s, 6H) ppm;

^{13}C{^{1}H}-NMR (101 MHz, C_6D_6): δ = 149.2, 105.2, 75.3, 69.5, 64.4, 36.9, 36.8, 33.6, 26.2, 18.6, 16.6, 15.2, 14.7, −5.16, −5.17 ppm;

HRMS (ESI) m/z: calcd for $C_{17}H_{36}O_3SiNa$ $[M+Na]^+$ 339.2331, found: 339.2333;

R*f* = 0.3 (PE:MTBE 4:1, vanillin);

$[\alpha]_D^{20}$ = −5.03 (*c* 0.4, $CHCl_3$).

(*S*)-Allylic alcohol 46

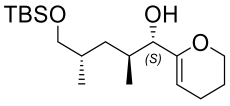

46
$C_{18}H_{36}O_3Si$
328.57

According to GP4, TIB ester **18** (221 mg, 0.46 mmol, 1.5 equiv),[8] vinyl boronic ester **30** (65.0 mg, 0.31 mmol, 1.0 equiv), *s*BuLi (0.33 mL, 0.43 mmol, 1.4 equiv) and (+)-sparteine (0.11 mL, 0.46 mmol, 1.5 equiv) gave (*S*)-allylic alcohol **46** (40.0 mg, 0.12 mmol, 39% o2s, dr ≥ 19:1) after purification by flash column chromatography (PE:MTBE 7:1 + 0.5% Et_3N) as a colorless oil.

^{1}H-NMR (400 MHz, C_6D_6): δ = 4.63 (t, J = 3.7 Hz, 1H), 3.75-3.60 (m, 3H), 3.46 (dd, J = 9.6, 6.1 Hz, 1H), 3.40 (dd, J = 9.6, 6.4 Hz, 1H), 2.05-1.95 (m, 1H), 1.85-1.75 (m, 3H), 1.60 (m_c, 1H), 1.53 (d, J = 6.7 Hz, 1H), 1.50-1.38 (m, 2H), 1.32 (m_c, 1H), 1.00 (s, 9H), 0.97 (d, J = 6.9 Hz, 3H), 0.95 (d, J = 6.7 Hz, 3H), 0.07 (s, 6H) ppm;

^{13}C{^{1}H}-NMR (101 MHz, C_6D_6): δ = 155.0, 96.9, 78.0, 69.7, 66.0, 36.0, 34.6, 33.9, 26.2, 22.8, 20.2, 18.6, 16.53, 16.52, −5.16, −5.17 ppm;

HRMS (ESI) m/z: calcd for $C_{18}H_{36}O_3SiNa$ $[M+Na]^+$ 351.2331, found: 351.2315;

R$_f$ = 0.3 (PE:MTBE 7:1, vanillin);

$[\alpha]_D^{20}$ = −26.0 (*c* 0.5, $CHCl_3$).

(*S*)-Allylic alcohol 47

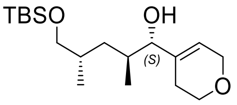

47
$C_{18}H_{36}O_3Si$
328.57

According to GP4, TIB ester **18** (221 mg, 0.46 mmol, 1.5 equiv),[8] vinyl boronic ester **31** (65.0 mg, 0.31 mmol, 1.0 equiv), *s*BuLi (0.33 mL, 0.43 mmol, 1.4 equiv) and (+)-sparteine (0.11 mL, 0.46 mmol, 1.5 equiv) gave (*S*)-allylic alcohol **47** (53.8 mg, 0.16 mmol, 52% o2s, dr ≥ 19:1) after purification by flash column chromatography (PE:MTBE 3:1) as a colorless oil.

^{1}H-NMR (400 MHz, C_6D_6): δ = 5.31 (m$_c$, 1H), 4.03 (m$_c$, 2H), 3.68-3.57 (m, 2H), 3.48-3.36 (m, 3H), 2.06-1.98 (m, 1H), 1.81-1.71 (m, 2H), 1.70-1.61 (m, 1H), 1.48 (m$_c$, 1H), 1.28 (m$_c$, 1H), 1.06 (brs, 1H), 1.00 (s, 9H), 0.91 (d, *J* = 6.6 Hz, 3H), 0.82 (d, *J* = 6.7 Hz, 3H), 0.08 (s, 6H) ppm;

^{13}C{^{1}H}-NMR (101 MHz, C_6D_6): δ = 137.8, 122.5, 80.5, 69.6, 65.4, 64.3, 35.6, 33.7, 33.4, 26.2, 24.7, 18.6, 16.6, 16.5, −5.2 ppm;

HRMS (ESI) m/z: calcd for $C_{18}H_{36}O_3SiNa$ $[M+Na]^+$ 351.2331, found: 351.2320;

R$_f$ = 0.3 (PE:MTBE 3:1, vanillin);

$[\alpha]_D^{20}$ = −24.0 (*c* 0.5, $CHCl_3$).

(*R*)-Allylic alcohol 48a

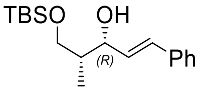

48a
$C_{18}H_{30}O_2Si$
306.52

According to GP4, literature known TIB ester **21** (213 mg, 0.49 mmol, 1.5 equiv),[10] vinyl boronic ester **32** (75.0 mg, 0.33 mmol, 1.0 equiv), *s*BuLi (0.35 mL, 0.46 mmol, 1.4 equiv) and (+)-sparteine (0.12 mL, 0.49 mmol, 1.5 equiv) gave allylic alcohol **48** (53.4 mg, 0.17 mmol, 52% o2s, dr 1:1) after purification by flash column chromatography (PE:MTBE 95:5) as a colorless oil.
According to GP5, carbamate **22** (162 mg, 0.49 mmol, 1.5 equiv), vinyl boronic ester **32** (75.0 mg, 0.33 mmol, 1.0 equiv), *s*BuLi (0.35 mL, 0.46 mmol, 1.4 equiv), (+)-sparteine (0.12 mL, 0.49 mmol, 1.5 equiv), magnesium turnings (15.8 mg, 0.66 mmol, 2.0 equiv) and dibromoethane (0.06 mL, 0.66 mmol, 2.0 equiv) gave (*R*)-allylic alcohol **48a** (45.1 mg, 0.15 mmol, 45% o2s, dr ≥ 19:1) after purification by flash column chromatography (PE:MTBE 95:5) as a colorless oil.

Analytical data are given for (*R*)-allylic alcohol **48a** obtained by the reaction of carbamate **22** and (+)-sparteine (dr ≥ 19:1):

^{1}H-NMR (400 MHz, $CDCl_3$): δ = 7.41-7.39 (m, 2H), 7.33-7.30 (m, 2H), 7.25-7.21 (m, 1H), 6.65 (dd, *J* = 15.9, 1.2 Hz, 1H), 6.26 (dd, *J* = 15.9, 5.7 Hz, 1H), 4.48 (brs, 1H), 3.76 (dd, *J* = 9.9, 4.2 Hz, 1H), 3.70 (dd, *J* = 9.9, 6.9 Hz, 1H), 3.49 (d, *J* = 4.9 Hz, 1H), 2.03 (m_c, 1H), 0.94-0.92 (m, 12H), 0.09 (s, 6H) ppm;

^{13}C{^{1}H}-NMR (101 MHz, $CDCl_3$): δ = 137.2, 130.4, 130.3, 128.7, 127.5, 126.5, 75.8, 67.5, 40.1, 26.0, 18.3, 11.5, −5.46, −5.48 ppm;

HRMS (ESI) m/z: calcd for $C_{18}H_{30}O_2SiNa$ $[M+Na]^+$ 329.1913, found: 329.1903;

R$_f$ = 0.3 (PE:MTBE 9:1, uv, vanillin);

$[\alpha]_D^{20}$ = −22.0 (*c* 0.5, $CHCl_3$).

(*S*)-Allylic alcohol 49

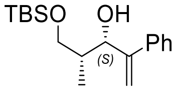

49

$C_{18}H_{30}O_2Si$

306.52

According to GP5, carbamate **22** (162 mg, 0.49 mmol, 1.5 equiv), vinyl boronic ester **33** (75.0 mg, 0.33 mmol, 1.0 equiv), *s*BuLi (0.35 mL, 0.46 mmol, 1.4 equiv), (+)-sparteine (0.12 mL, 0.49 mmol, 1.5 equiv), magnesium turnings (15.8 mg, 0.66 mmol, 2.0 equiv) and dibromoethane (0.06 mL, 0.66 mmol, 2.0 equiv) gave (*S*)-allylic alcohol **49** (79.3 mg, 0.26 mmol, 79% o2s, dr ≥ 19:1) after purification by flash column chromatography (PE:MTBE 95:5) as a colorless oil.

^{1}H-NMR (400 MHz, $CDCl_3$): δ = 7.36-7.26 (m, 5H), 5.43 (t, *J* = 1.8 Hz, 1H), 5.35 (t, *J* = 1.6 Hz, 1H), 5.03 (m_c, 1H), 3.77 (dd, *J* = 9.8, 3.6 Hz, 1H), 3.66 (dd, *J* = 9.8, 5.1 Hz, 1H), 3.32 (d, *J* = 2.5 Hz, 1H), 1.67 (m_c, 1H), 0.92 (s, 9H), 0.80 (d, *J* = 7.1 Hz, 3H), 0.07 (s, 6H) ppm;

^{13}C{^{1}H}-NMR (101 MHz, $CDCl_3$): δ = 150.1, 140.8, 128.5, 127.6, 127.0, 112.9, 75.0, 68.3, 36.9, 26.0, 18.3, 9.1, −5.4, −5.5 ppm;

HRMS (ESI) m/z: calcd for $C_{18}H_{30}O_2Na$ $[M+Na]^+$ 329.1913, found: 329.1914;

R$_f$ = 0.3 (PE:MTBE 95:5, uv, vanillin);

$[\alpha]_D^{20}$ = +62.1 (*c* 0.7, $CHCl_3$).

(*R*)-Allylic alcohol 50

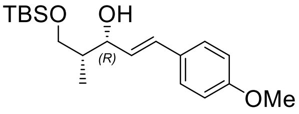

50
$C_{19}H_{32}O_3Si$
336.55

According to GP5, carbamate **22** (143 mg, 0.43 mmol, 1.5 equiv), vinyl boronic ester **34** (75.0 mg, 0.29 mmol, 1.0 equiv), *s*BuLi (0.31 mL, 0.40 mmol, 1.4 equiv), (+)-sparteine (0.10 mL, 0.43 mmol, 1.5 equiv), magnesium turnings (14.0 mg, 0.58 mmol, 2.0 equiv) and dibromoethane (0.05 mL, 0.58 mmol, 2.0 equiv) gave (*R*)-allylic alcohol **50** (65.3 mg, 0.19 mmol, 66% o2s, dr ≥ 19:1) after purification by flash column chromatography (PE:MTBE 9:1) as a colorless oil.

^{1}H-NMR (400 MHz, C_6D_6): δ = 7.28-7.24 (m, 2H), 6.79-6.75 (m, 2H), 6.71 (dd, J = 15.9, 1.0 Hz, 1H), 6.14 (dd, J = 15.9, 5.7 Hz, 1H), 4.46 (brs, 1H), 3.62 (dd, J = 9.8, 6.6 Hz, 1H), 3.56 (dd, J = 9.8, 4.8 Hz, 1H), 3.30 (s, 3H), 2.48 (d, J = 3.9 Hz, 1H), 1.87 (m_c, 1H), 0.96-0.94 (m, 12H), 0.03 (s, 6H) ppm;

^{13}C{^{1}H}-NMR (101 MHz, C_6D_6): δ = 159.7, 130.5, 130.0, 129.4, 128.0, 114.4, 74.7, 67.0, 54.8, 41.2, 26.1, 18.4, 11.4, −5.42, −5.43 ppm;

HRMS (ESI) m/z: calcd for $C_{19}H_{32}O_3SiNa$ $[M+Na]^+$ 359.2018, found: 359.2016;

R_f = 0.3 (PE:MTBE 9:1, uv, vanillin);

$[\alpha]_D^{20}$ = −17.9 (*c* 0.7, $CHCl_3$).

(*R*)-Allylic alcohol 51

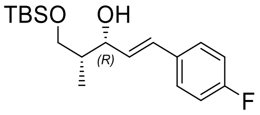

51
$C_{18}H_{29}FO_2Si$
324.51

According to GP5, carbamate **22** (150 mg, 0.45 mmol, 1.5 equiv), vinyl boronic ester **35** (75.0 mg, 0.30 mmol, 1.0 equiv), *s*BuLi (0.33 mL, 0.42 mmol, 1.4 equiv), (+)-sparteine (0.10 mL, 0.45 mmol, 1.5 equiv), magnesium turnings (14.7 mg, 0.60 mmol, 2.0 equiv) and dibromoethane (0.05 mL, 0.60 mmol, 2.0 equiv) gave (*R*)-allylic alcohol **51** (40.4 mg, 0.12 mmol, 40% o2s, dr ≥ 19:1) after purification by flash column chromatography (PE:MTBE 9:1) as a colorless oil.

^{1}H-NMR (400 MHz, $CDCl_3$): δ = 7.37-7.32 (m, 2H), 7.02-6.98 (m, 2H), 6.61 (dd, J = 15.9, 1.3 Hz, 1H), 6.17 (dd, J = 15.9, 5.6 Hz, 1H), 4.46 (brs, 1H), 3.77 (dd, J = 9.9, 4.1 Hz, 1H), 3.69 (dd, J = 9.9, 6.9 Hz, 1H), 3.50 (d, J = 4.9 Hz, 1H), 2.01 (m_c, 1H), 0.93-0.91 (m, 12H), 0.09 (s, 3H), 0.08 (s, 3H) ppm;

^{13}C{^{1}H}-NMR (101 MHz, $CDCl_3$): δ = 162.3 (d, J = 246.0 Hz), 133.5 (d, J = 3.3 Hz), 130.1 (d, J = 2.2 Hz), 129.3, 128.0 (d, J = 8.0 Hz), 115.5 (d, J = 21.5 Hz), 75.8, 67.6, 40.0, 26.0, 18.3, 11.4, −5.46, −5.48 ppm;

HRMS (ESI) m/z: calcd for $C_{18}H_{29}FO_2SiNa$ $[M+Na]^+$ 347.1819, found: 347.1822;

R$_f$ = 0.3 (PE:MTBE 9:1, uv, vanillin);

$[\alpha]_D^{20}$ = −14.9 (c 0.9, $CHCl_3$).

2-4. Synthesis of (−)-sachalinol A

Alcohol S1

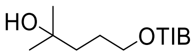

S1
$C_{22}H_{36}O_3$
348.53

To a stirred solution of ketone **61** (1.02 g, 3.06 mmol, 1.0 equiv) in THF (10.0 mL) at 0 °C was added MeMgBr (3.0 M in Et_2O, 1.5 mL, 4.59 mmol, 1.5 equiv). The reaction mixture was was warmed to rt and stirred for 4 h at that temperature. The reaction mixture was cooled to 0 °C, sat. aq. NH_4Cl was added, and the phases were separated. The aqueous layer was extracted with MTBE (3x). The combined organic phases were dried over Na_2SO_4 and concentrated *in vacuo*. The crude material was purified by flash column chromatography (PE:MTBE 3:1) to afford alcohol **S1** (0.91 g, 2.61 mmol, 85%) as a colorless oil.

^{1}H-NMR (400 MHz, $CDCl_3$): δ = 7.01 (s, 2H), 4.33 (t, J = 6.6 Hz, 2H), 2.94-2.80 (m, 3H), 1.87-1.79 (m, 2H), 1.61-1.56 (m, 2H), 1.26-1.23 (m, 24H) ppm;

^{13}C{^{1}H}-NMR (101 MHz, $CDCl_3$): δ = 171.1, 150.2, 144.9, 130.7, 121.0, 70.7, 65.5, 40.3, 34.6, 31.6, 29.5, 24.3, 24.1, 23.8 ppm;

HRMS (ESI) m/z: calcd for $C_{22}H_{36}O_3Na$ $[M+Na]^+$ 371.2562, found: 371.2565;

R$_f$ = 0.2 (PE:MTBE 3:1, uv, vanillin).

TBS-ether 57

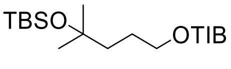

57
$C_{28}H_{50}O_3Si$
462.79

To a solution of alcohol **S1** (0.87 g, 2.49 mmol, 1.0 equiv) in CH_2Cl_2 (5.0 mL) at −78 °C were added 2,6-lutidine (0.87 mL, 7.47 mmol, 3.0 equiv) and TBSOTf (0.86 mL, 3.74 mmol, 1.5 equiv) successively. The reaction mixture was stirred for 10 min at −78 °C and then 1.5 h at 0 °C. After the addition of sat. aq. $NaHCO_3$ the organic layer was separated, and the aqueous layer was extracted with CH_2Cl_2 (3x). The combined organic layers were washed with aq. $KHSO_4$ (1.0 M) and sat. aq. NaCl, dried over Na_2SO_4 and concentrated *in vacuo*. The crude

material was purified by flash column chromatography (PE:MTBE 100:1) to afford TBS-ether **57** (1.01 g, 2.18 mmol, 88%) as a colorless oil.

^{1}H-NMR (400 MHz, $CDCl_3$): δ = 7.00 (s, 2H), 4.30 (t, J = 6.8 Hz, 2H), 2.94-2.81 (m, 3H), 1.86-1.79 (m, 2H), 1.53-1.49 (m, 2H), 1.25 (d, J = 6.8 Hz, 18H), 1.21 (s, 6H), 0.84 (s, 9H), 0.07 (s, 6H) ppm;

^{13}C{^{1}H}-NMR (101 MHz, $CDCl_3$): δ = 171.1, 150.2, 144.9, 130.9, 121.0, 73.1, 65.9, 41.5, 34.6, 31.6, 29.9, 26.0, 24.3, 24.1, 23.9, 18.2, −2.0 ppm;

HRMS (ESI) m/z: calcd for $C_{28}H_{50}O_3SiNa$ $[M+Na]^+$ 485.3427, found: 485.3427;

R$_f$ = 0.5 (PE:MTBE 100:1, uv, vanillin).

Allylic alcohol 62

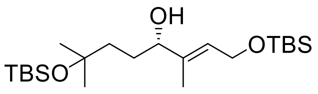

62
$C_{22}H_{48}O_3Si_2$
416.79

According to GP4, TIB ester **57** (389 mg, 0.84 mmol, 1.5 equiv), literature known vinyl boronic ester **58** (175 mg, 0.56 mmol, 1.0 equiv),[11] *s*BuLi (0.60 mL, 0.78 mmol, 1.4 equiv) and (+)-sparteine (0.19 mL, 0.84 mmol, 1.5 equiv) gave allylic alcohol **62** (157 mg, 0.38 mmol, 68% o2s, er ≥ 19:1) after purification by flash column chromatography (PE:MTBE 7:1) as a colorless oil.

^{1}H-NMR (400 MHz, C_6D_6): δ = 5.66 (m_c, 1H), 4.23 (m_c, 2H), 3.80 (m_c, 1H), 1.73-1.64 (m, 2H), 1.60-1.52 (m, 4H), 1.42-1.33 (m, 1H), 1.13 (s, 3H), 1.12 (s, 3H), 1.02-0.99 (m, 19H), 0.114-0.105 (m, 12H) ppm;

^{13}C{^{1}H}-NMR (101 MHz, C_6D_6): δ = 139.2, 125.9, 77.6, 73.6, 60.1, 41.3, 30.2, 30.1, 29.8, 26.197, 26.191, 18.5, 18.4, 11.9, −1.80, −1.81, −4.897, −4.904 ppm;

HRMS (ESI) m/z: calcd for $C_{22}H_{48}O_3Si_2Na$ $[M+Na]^+$ 439.3040, found: 439.3037;

R$_f$ = 0.3 (PE:MTBE 7:1, vanillin);

$[\boldsymbol{\alpha}]_{\mathbf{D}}^{\mathbf{20}}$ = −3.85 (c 0.3, $CHCl_3$).

(−)-Sachalinol A (55)

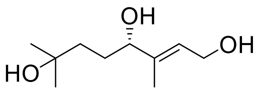

55
$C_{10}H_{20}O_3$
188.27

To a solution of allylic alcohol **62** (60.0 mg, 0.14 mmol, 1.0 equiv) in THF (1.4 mL) at 0 °C was added TBAF (1.0 M in THF, 1.4 mL, 1.40 mmol, 10.0 equiv). The reaction mixture was

warmed to 65 °C and stirred for 4 h at that temperature. The solvent was removed *in vacuo* and the crude material was purified by flash column chromatography (PE:acetone 1:1) to afford (−)-sachalinol A (**55**) (25.1 mg, 0.13 mmol, 93%) as a colorless oil.

^{1}H-NMR (400 MHz, CD_3OD): δ = 5.55 (m_c, 1H), 4.13 (d, J = 6.5 Hz, 2H), 3.91 (t, J = 6.5 Hz, 1H), 1.64 (s, 3H), 1.62-1.49 (m, 3H), 1.41-1.33 (m, 1H), 1.180 (s, 3H), 1.176 (s, 3H) ppm;

^{13}C{^{1}H}-NMR (101 MHz, CD_3OD): δ = 140.8, 126.2, 78.6, 71.1, 59.2, 40.8, 30.6, 29.3, 29.2, 11.6 ppm;

HRMS (ESI) m/z: calcd for $C_{10}H_{20}O_3Na$ $[M+Na]^+$ 211.1310, found: 211.1308;

R$_f$ = 0.3 (PE:acetone 1:1, vanillin);

$[\alpha]_D^{20}$ = −12.7 (*c* 0.6, MeOH).

Analytical data are in accordance with the literature.[12, 13]

2-5. Synthesis of (−)-rosiridol

Allylic alcohol 64

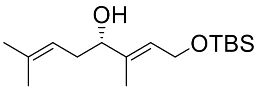

64

$C_{16}H_{32}O_2Si$

284.52

According to GP4, TIB ester **59** (1.58 g, 4.80 mmol, 1.5 equiv), literature known vinyl boronic ester **58** (1.00 g, 3.20 mmol, 1.0 equiv),[11] *s*BuLi (3.4 mL, 4.48 mmol, 1.4 equiv) and (+)-sparteine (1.1 mL, 4.80 mmol, 1.5 equiv) gave allylic alcohol **64** (0.63 g, 2.21 mmol, 69% o2s, er ≥ 19:1) after purification by flash column chromatography (PE:MTBE 9:1 → 7:1) as a colorless oil.

^{1}H-NMR (400 MHz, C_6D_6): δ = 5.71 (m_c, 1H), 5.16 (m_c, 1H), 4.23 (m_c, 2H), 3.87 (m_c, 1H), 2.31-2.17 (m, 2H), 1.62 (d, J = 0.9 Hz, 3H), 1.55 (d, J = 0.7 Hz, 3H), 1.50 (s, 3H), 1.16 (brs, 1H), 1.00 (s, 9H), 0.10 (s, 6H) ppm;

^{13}C{^{1}H}-NMR (101 MHz, C_6D_6): δ = 138.6, 133.9, 126.0, 121.0, 76.7, 60.2, 34.6, 26.2, 25.9, 18.5, 17.9, 12.0, −4.91, −4.92 ppm;

HRMS (ESI) m/z: calcd for $C_{16}H_{32}O_2SiNa$ $[M+Na]^+$ 307.2069, found: 307.2067;

R$_f$ = 0.5 (PE:MTBE 95:5, vanillin);

$[\alpha]_D^{20}$ = −19.1 (*c* 1.4, $CHCl_3$).

(−)-Rosiridol 56

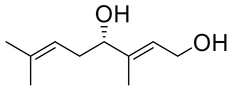

56
$C_{10}H_{18}O_2$
170.25

To a solution of allylic alcohol **64** (50.0 mg, 0.18 mmol, 1.0 equiv) in THF (1.8 mL) at 0 °C was added TBAF (1.0 M in THF, 0.90 mL, 0.90 mmol, 5.0 equiv). The reaction mixture was warmed to rt and stirred for 1 h at that temperature. The solvent was removed *in vacuo* and the crude material was purified by flash column chromatography (PE:MTBE 1:3) to afford (−)-rosiridol (**56**) (29.7 mg, 0.17 mmol, 94%) as a colorless oil.

^{1}H-NMR (400 MHz, $CDCl_3$): δ = 5.66 (m_c, 1H), 5.11 (m_c, 1H), 4.21 (m_c, 2H), 4.01 (m_c, 1H), 2.32-2.22 (m, 2H), 1.73 (s, 3H), 1.68 (s, 3H), 1.64 (s, 3H) ppm;

^{13}C{^{1}H}-NMR (101 MHz, $CDCl_3$): δ = 140.5, 135.5, 124.6, 119.8, 76.5, 59.3, 34.3, 26.0, 18.1, 12.3 ppm;

HRMS (EI) m/z: mass itself not found, but the following fragment calcd for C_2H_9 $[M-C_8H_9O_2]^+$ 69.0699, found: 69.0704;

R*f* = 0.3 (PE:MTBE 1:3, vanillin);

$[\alpha]_D^{20}$ = −7.21 (*c* 1.1, acetone).

Analytical data are in accordance with the literature.[12–14]

2-6. Analysis of enantioselectivity

General Procedure 7 (GP7): Mosher ester

To a stirred solution of allylic alcohol (1.0 equiv) in CH_2Cl_2 (0.02 M) were added Et_3N (10.0 equiv), DMAP (6.0 equiv) and (*R*)-MTPACl (6.0 equiv) successively. The reaction mixture was stirred at rt until TLC showed full conversion. After the addition of MTBE and aq. NaOH (2.0 M) the organic layer was separated. The organic layer was washed with aq. NaOH (2.0 M, 3x), sat. aq. $NaHCO_3$ (3x), aq. $CuSO_4$ (1.0 M) and sat. aq. NaCl, dried over Na_2SO_4 and concentrated *in vacuo*. Finally, the ^{1}H-NMR spectrum was analyzed for potential diastereoisomers.

(*S*)-Mosher ester S2

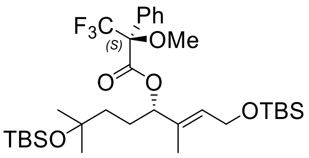

S2
$C_{32}H_{55}F_3O_5Si_2$
632.95

Following GP7, allylic alcohol **62** (25.0 mg, 60.0 µmol, 1.0 equiv), Et_3N (84 µL, 600 µmol, 10.0 equiv), DMAP (44.0 mg, 360 µmol, 6.0 equiv) and (*R*)-MTPACl (67 µL, 360 µmol, 6.0 equiv) gave (*S*)-mosher ester **S2** (32.5 mg, 51.3 µmol, 86%, dr ≥ 19:1) as a yellow oil.

^{1}H-NMR (400 MHz, C_6D_6): δ = 7.72 (d, J = 7.8 Hz, 2H), 7.16-7.04 (m, 3H), 5.85 (m$_c$, 1H), 5.50 (m$_c$, 1H), 4.14 (d, J = 6.0 Hz, 2H), 3.49 (s, 3H), 1.95-1.85 (m, 1H), 1.78-1.69 (m, 1H), 1.57 (s, 3H), 1.36-1.19 (m, 2H), 1.02 (s, 3H), 1.01 (s, 3H), 0.97 (s, 9H), 0.96 (s, 9H), 0.07-0.06 (m, 12H) ppm.

(*S*)-Mosher ester S3

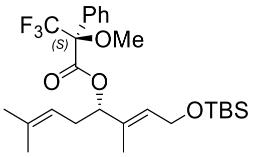

S3
$C_{26}H_{39}F_3O_4Si$
500.67

Following GP7, allylic alcohol **64** (16.0 mg, 56.2 µmol, 1.0 equiv), Et_3N (78 µL, 562 µmol, 10.0 equiv), DMAP (41.2 mg, 337 µmol, 6.0 equiv) and (*R*)-MTPACl (63 µL, 337 µmol, 6.0 equiv) gave (*S*)-mosher ester **S3** (26.1 mg, 52.1 µmol, 93%, dr ≥ 19:1) as a yellow oil.

^{1}H-NMR (400 MHz, C_6D_6): δ = 7.71 (d, J = 7.6 Hz, 2H), 7.13-7.03 (m, 3H), 5.82 (m$_c$, 1H), 5.51 (m$_c$, 1H), 4.96 (m$_c$, 1H), 4.12 (d, J = 5.9 Hz, 2H), 3.46 (s, 3H), 2.45-2.38 (m, 1H), 2.22-2.15 (m, 1H), 1.54 (s, 3H), 1.51 (s, 3H), 1.39 (s, 3H), 0.97 (s, 9H), 0.05 (s, 6H) ppm.

3 References

[1] P. Beak, L. G. Carter. Dipole-stabilized carbanions from esters: .alpha.-oxo lithiations of 2,6-substituted benzoates of primary alcohols. *J. Org. Chem.* **1981**, *46*, 2363.

[2] Lautens, M.; Colucci, J. T.; Hiebert, S.; Smith, N. D.; Bouchain, G. Total Synthesis of Ionomycin Using Ring-Opening Strategies. *Org. Lett.* **2002**, *4*, 1879–1882.

[3] Rink, C.; Navickas, V.; Maier, M. E. An Approach to the Core Structure of Leiodermatolide. *Org. Lett.* **2011**, *13*, 2334–2337.

[4] Hoppe, D.; Hintze, F.; Tebben, P.; Paetow, M.; Ahrens, H.; Schwerdtfeger, J.; Sommerfeld, P.; Haller, J.; Guarnieri, W.; Kolczewski, S.; Hense, T.; Hoppe, I. Enantioselective synthesis via sparteine-induced asymmetric deprotonation. *Pure & Appl. Chem.* **1994**, *66*, 1479–1486.

[5] Würthwein, E.-U.; Hoppe, D. Enantioselective Lithiation of O-Alkyl and O-Alk-2-enyl Carbamates in the Presence of (−)-Sparteine and (−)-α-Isosparteine. A Theoretical Study. *J. Org. Chem.* ***2005***, *70*, 4443–4451.

[6] Linne, Y.; Bonandi, E.; Tabet, C.; Geldsetzer, J.; Kalesse, M. The Total Synthesis of Chondrochloren A. *Angew. Chem. Int. Ed.* **2021**, *60*, 6938−6942.

[7] Linne, Y.; Birkner, M.; Flormann, J.; Lücke, D.; Becker, J. A.; Kalesse, M. Sparteine-Free, Highly Stereoselective Construction of Complex Allylic Alcohols Using 1,2-Metallate Rearrangements. *JACSAu* **2023**, *3*, 1695–1710.

[8] Linne, Y.; Birkner, M.; Kalesse, M. Synthesis of the C18–C27 Fragment of Georatusin. *Arkivoc* **2021**, *4*, 152−167.

[9] Linne, Y.; Schönwald, A.; Weißbach, S.; Kalesse, M. Desymmetrization of C2-Symmetric Bis(Boronic Esters) by Zweifel Olefinations. *Chem. Eur. J.* **2020**, *26*, 7998–8002.

[10] García-Ruiz, C.; Chen, J. L. Y.; Sandford, C.; Feeney, K.; Lorenzo, P.; Berionni, G.; Mayr, H.; Aggarwal, V. K. Stereospecific Allylic Functionalization: The Reactions of Allylboronate Complexes with Electrophiles. *J. Am. Chem. Soc.* **2017**, *139*, 15324−15327.

[11] Hesse, M. J.; Butts, C. P.; Willis, C. L.; Aggarwal, V. K. Diastereodivergent Synthesis of Trisubstituted Alkenes through Protodeboronation of Allylic Boronic Esters: Application to the Synthesis of the Californian Red Scale Beetle Pheromone. *Angew. Chem. Int. Ed.* **2012**, *51*, 12444–12448.

[12] (a) Fan, W.; Tezuka, Y.; Ni, K. M.; Kadota, S. Prolyl Endopeptidase Inhibitors from the Underground Part of Rhodiola sachalinensis. *Chem. Pharm. Bull.* **2001**, *49*, 396–401; (b) Ma, G.; Li, W.; Dou, D.; Chang, X.; Bai, H.; Satou, T.; Li, J.; Sun, D.; Kang, T.; Nikaido, T.; Koike, K. Rhodiolosides A–E, Monoterpene Glycosides from Rho-diola rosea. *Chem. Pharm. Bull.* **2006**, *54*, 1229–1233; (c) Li, W.; Dou, D.; Koike, K. Revised Absolute Stereochemistry of Rhodiolosides A–D, Rhodiolol A and Sachalinol A from Rhodiola rosea. *Chem. Pharm. Bull.* **2008**, *56*, 1047–1048.

[13] For previous syntheses of sachalinol A and rosiridol see: (a) Simon, K.; Jones, P. G.; Lindel, T. Total Syntheses of Rhodiolosides A and D and of Sachalinols A–C. *Eur. J. Org. Chem.* **2011**, *2011*, 1493–1503; (b) Schöttner, E.; Simon, K.; Friedel, M.; Jones, P. G.; Lindel, T. Synthesis and stereochemistry of (−)-rosiridol and (−)-rosiridin. *Tet. Lett.* **2008**, *49*, 5580–5582; (c) Díez, D.; Nuñez, M. G.; Moro, R. F.; Antón, A. B.; Garrido, N. M.; Marcos, I. S.; Basabe P. Use of Nitriles in Synthesis. First Total Synthesis of ent-Sachalinol A. *Synlett* **2006**, *11*, 1715–1716; (d) Hong, B.-C.; Hong, J.-H.; Tsai Y.-C. Regio- and Enantioselective Prenyl Anion Transfer: Application to the Total Synthesis of (−)-Rosiridol. *Angew. Chem. Int. Ed.* **1998**, *37*, 468–470.

[14] Yoshikawa, M.; Nakamura, S.; Li, X.; Matsuda, H. Reinvestigation of Absolute Stereostructure of (−)-Rosiridol: Structures of Monoterpene Glycosides, Rosiridin, Rosiridosides A, B, and C, from Rhodiola sachalinensis. *Chem. Pharm. Bull.* **2008**, *56*, 695–700.

4 Spectra

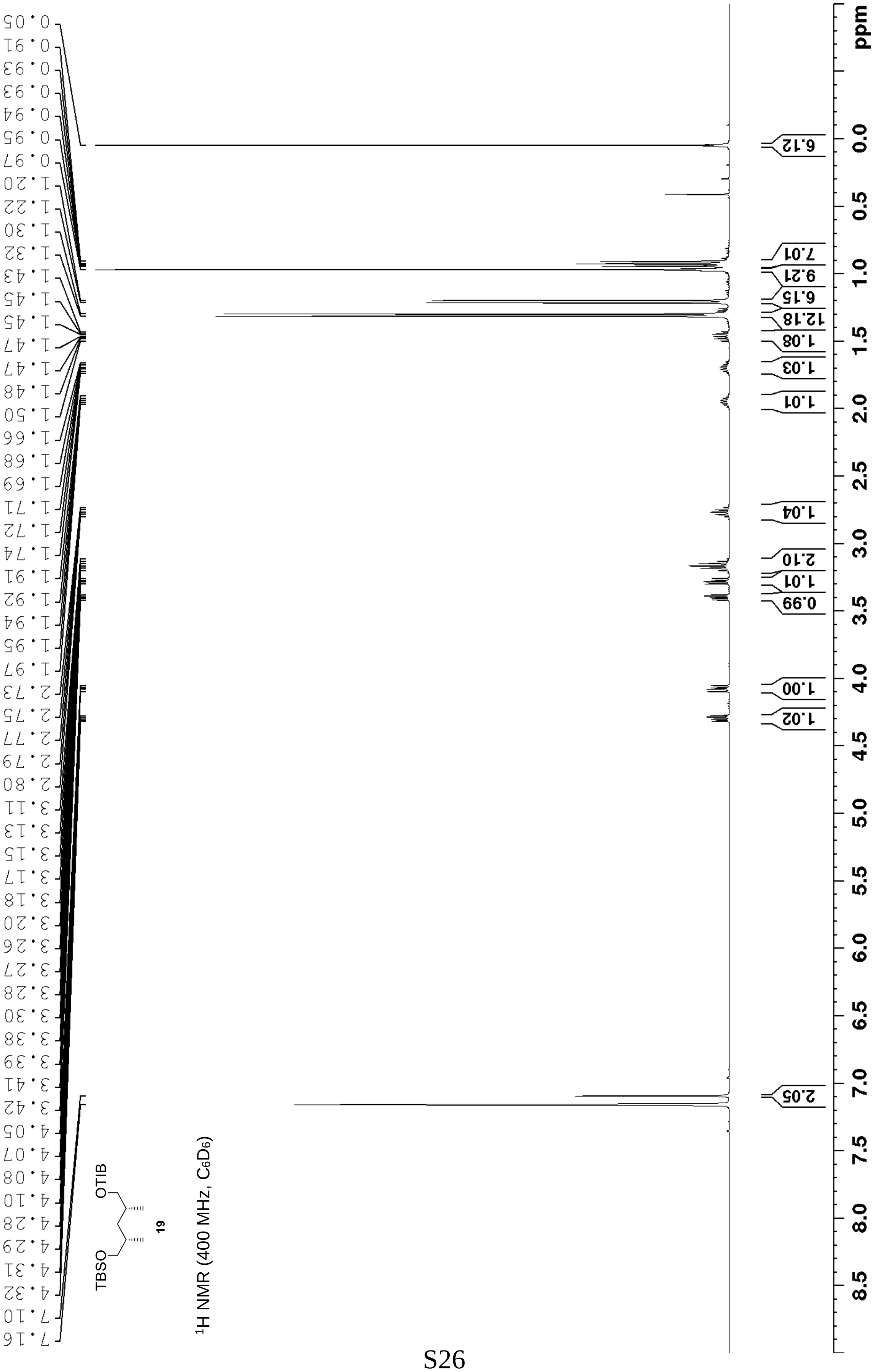
0.05
0.91
0.93
0.93
0.94
0.95
0.97
1.20
1.22
1.30
1.32
1.43
1.45
1.45
1.47
1.47
1.48
1.50
1.66
1.68
1.69
1.71
1.72
1.74
1.91
1.92
1.94
1.95
1.97
2.73
2.75
2.77
2.79
2.80
3.11
3.13
3.15
3.17
3.18
3.20
3.26
3.27
3.28
3.30
3.38
3.39
3.41
3.42
4.05
4.07
4.08
4.10
4.28
4.29
4.31
4.32
7.10
7.16
TBSO
OTIB
19
1H NMR (400 MHz, C6D6)
6.12
7.01
9.21
6.15
12.18
1.08
1.03
1.01
1.04
2.10
1.01
0.99
1.00
1.02
2.05
ppm
0.0
0.5
1.0
1.5
2.0
2.5
3.0
3.5
4.0
4.5
5.0
5.5
6.0
6.5
7.0
7.5
8.0
8.5

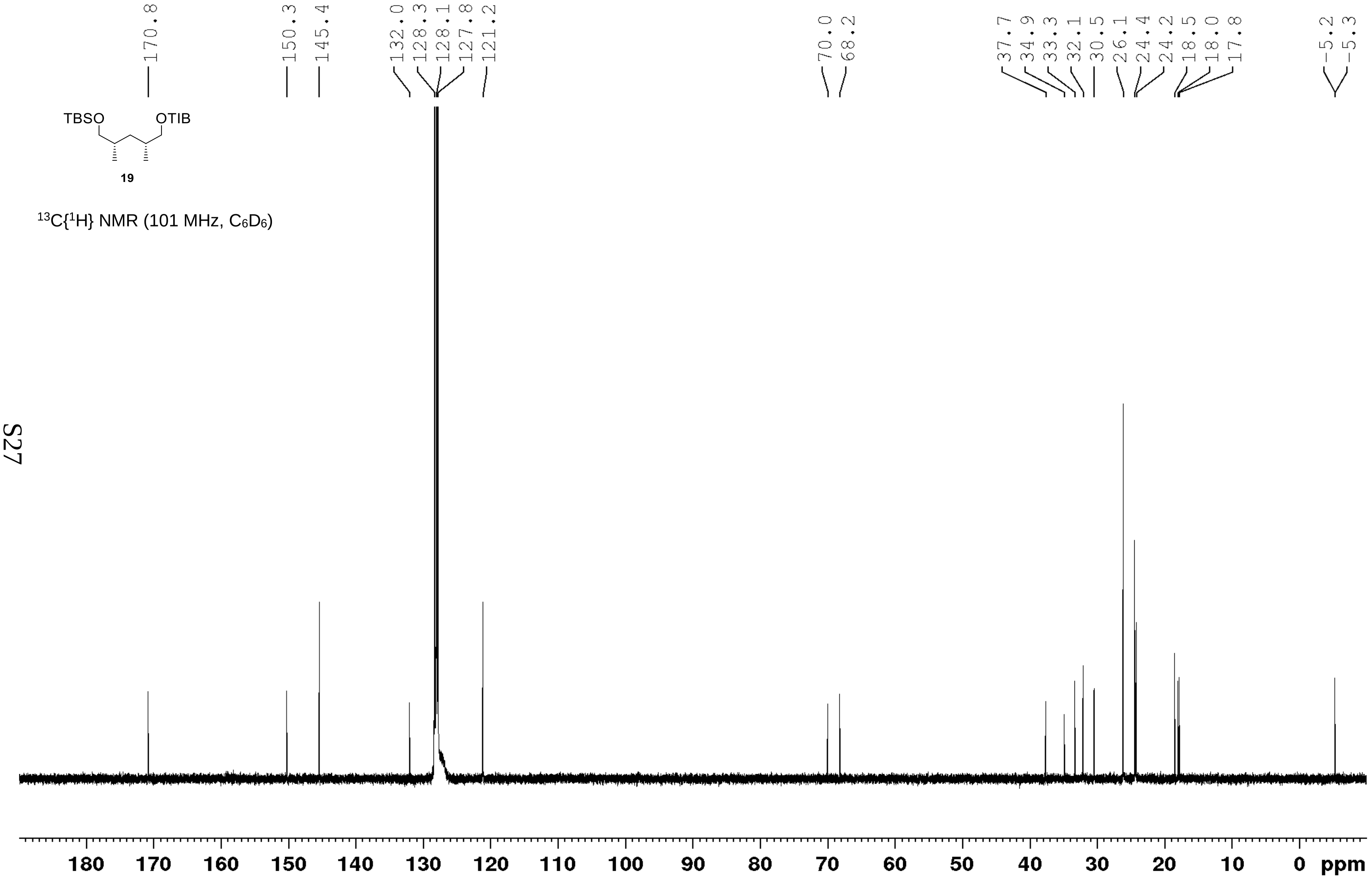

$^{13}C\{^{1}H\}$ NMR (101 MHz, C_6D_6)

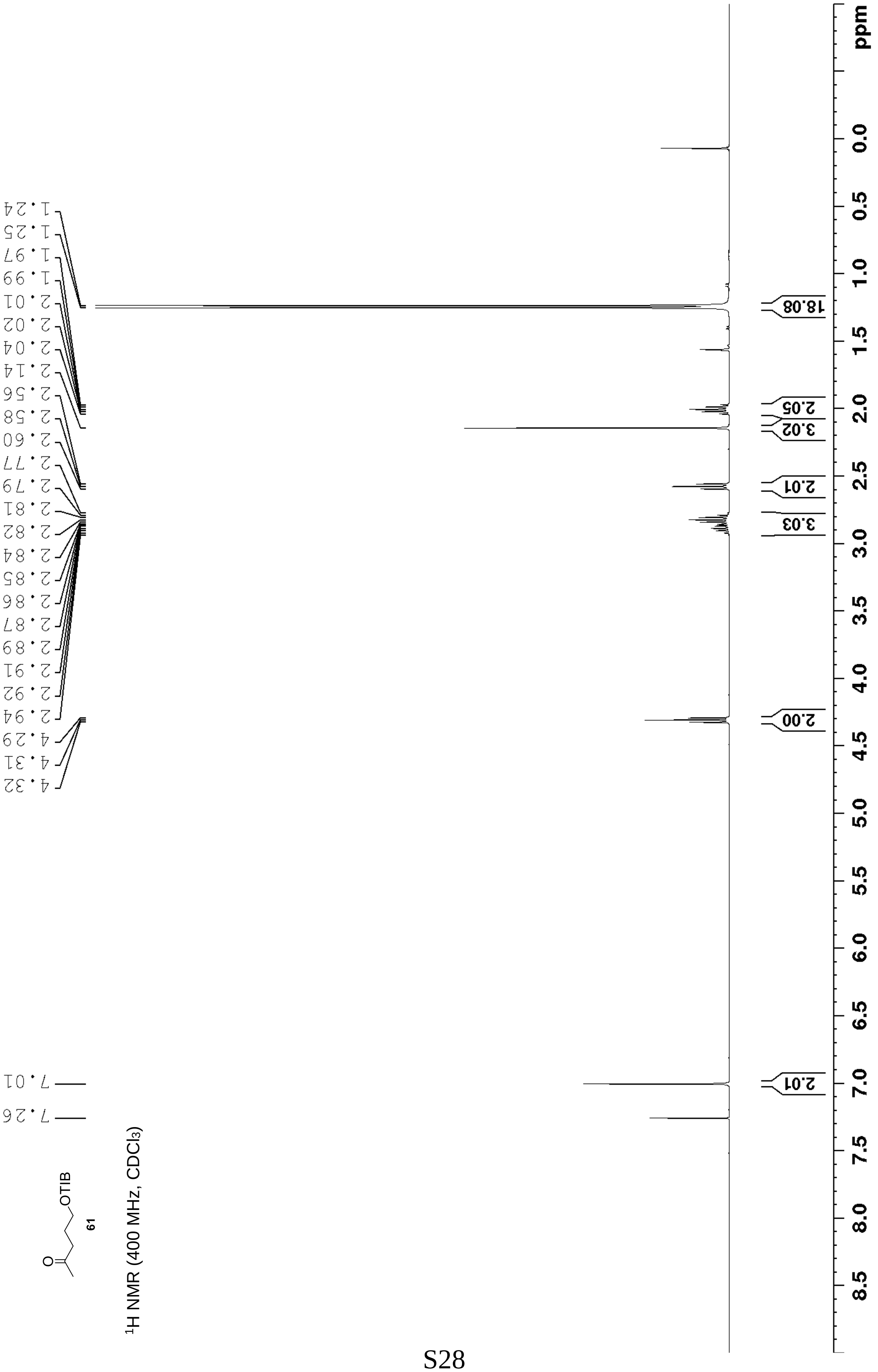
1H NMR (400 MHz, CDCl3)
OTIB
61
7.26
7.01
4.32
4.31
4.29
2.94
2.92
2.91
2.89
2.87
2.86
2.85
2.84
2.82
2.81
2.79
2.77
2.60
2.58
2.56
2.14
2.04
2.02
2.01
1.99
1.97
1.25
1.24
2.01
2.00
3.03
2.01
3.02
2.05
18.08
ppm

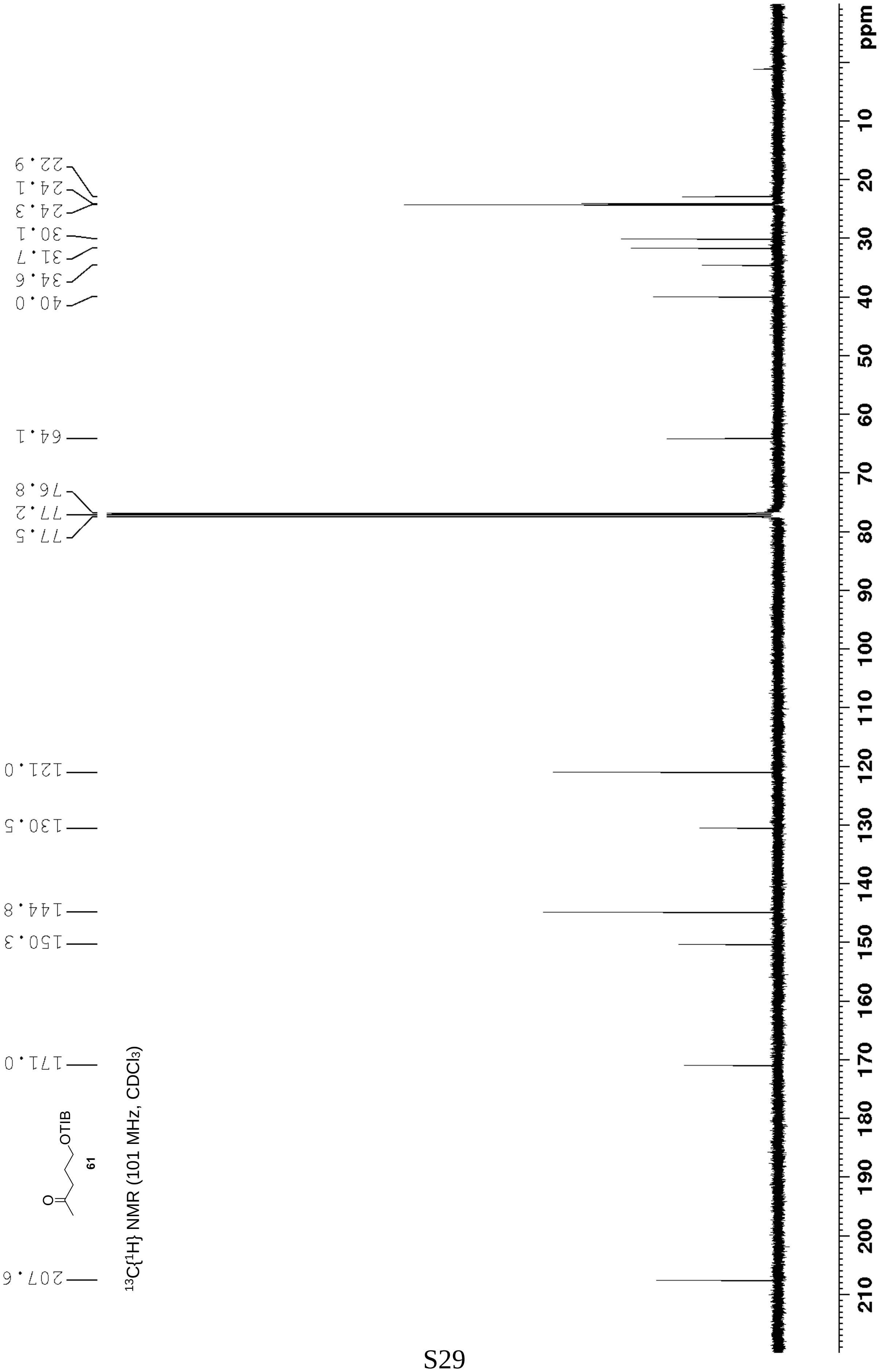
OTIB
61
13C{1H} NMR (101 MHz, CDCl3)
207.6
171.0
150.3
144.8
130.5
121.0
77.5
77.2
76.8
64.1
40.0
34.6
31.7
30.1
24.3
24.1
22.9
ppm

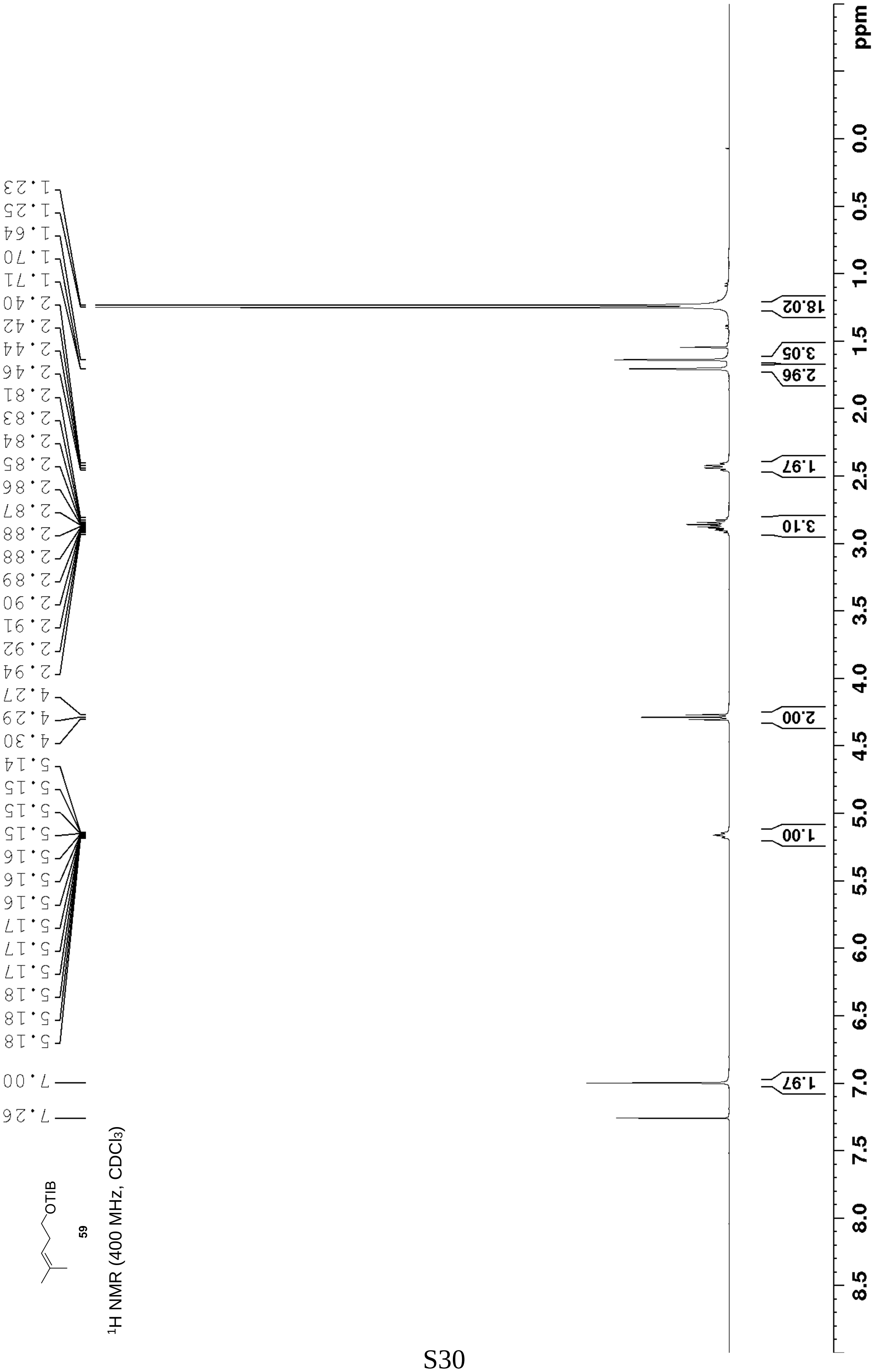
OTIB
59
1H NMR (400 MHz, CDCl3)
ppm
18.02
3.05
2.96
1.97
3.10
2.00
1.00
1.97

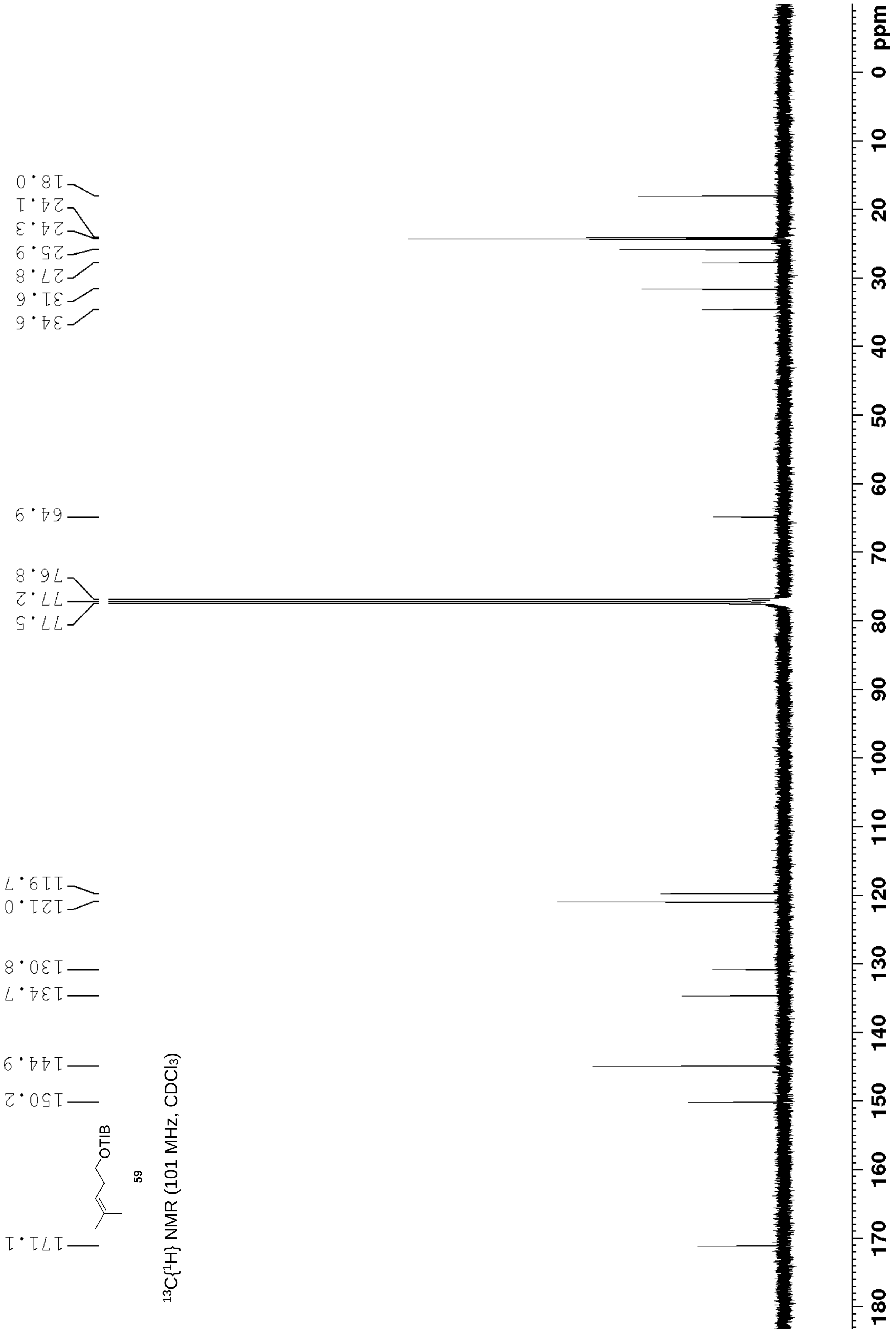

171.1
150.2
144.9
134.7
130.8
121.0
119.7
77.5
77.2
76.8
64.9
34.6
31.6
27.8
25.9
24.3
24.1
18.0
OTIB
59
13C{1H} NMR (101 MHz, CDCl3)
ppm

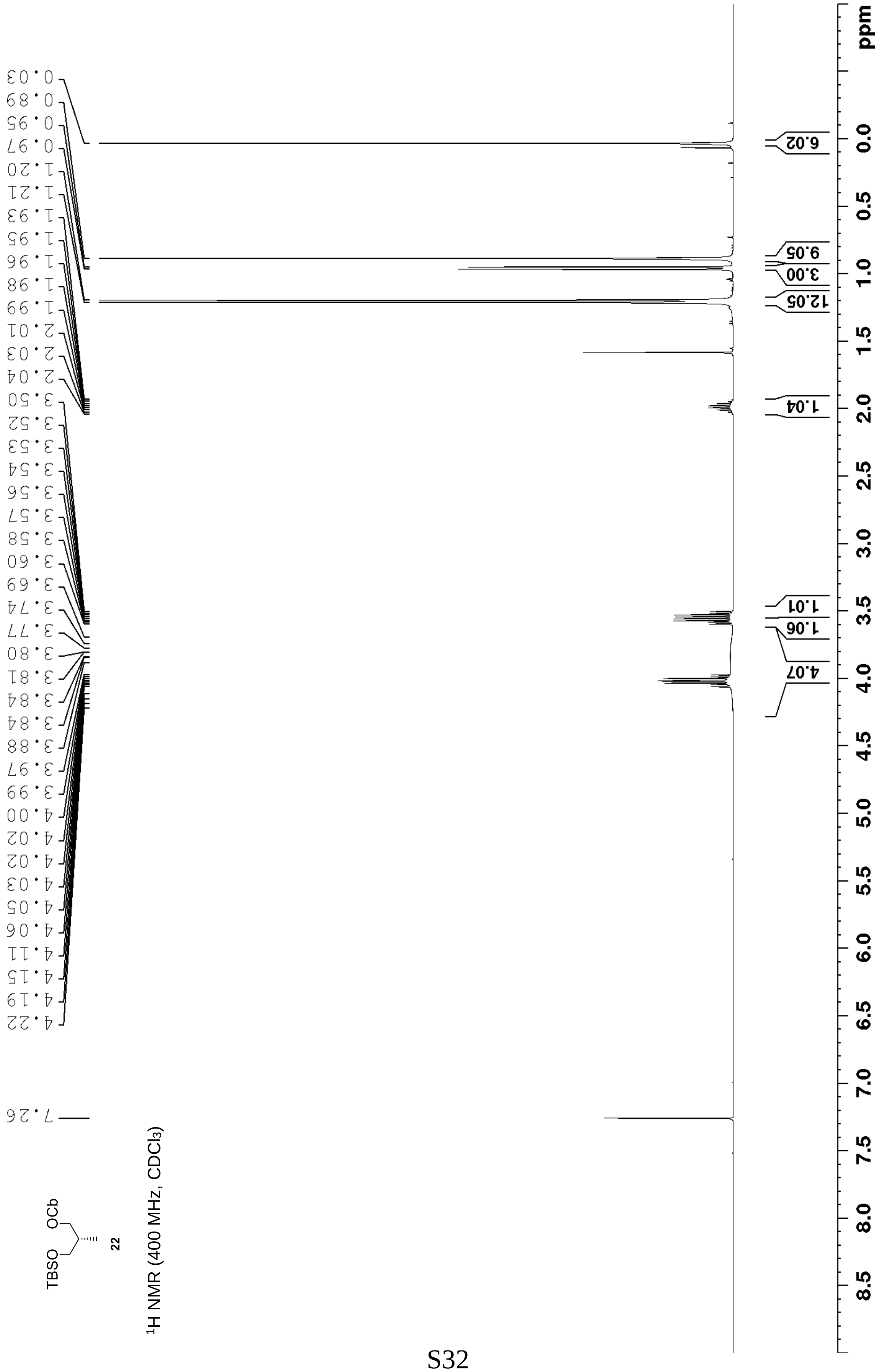
TBSO OCb
22
1H NMR (400 MHz, CDCl3)
0.03 0.89 0.95 0.97 1.20 1.21 1.93 1.95 1.96 1.98 1.99 2.01 2.03 2.04 3.50 3.52 3.53 3.54 3.56 3.57 3.58 3.60 3.69 3.74 3.77 3.80 3.81 3.84 3.84 3.88 3.97 3.99 4.00 4.02 4.02 4.03 4.05 4.06 4.11 4.15 4.19 4.22
7.26
6.02
9.05
3.00
12.05
1.04
1.01
1.06
4.07
ppm

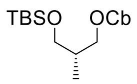

22

$^{13}C\{^{1}H\}$ NMR (101 MHz, $CDCl_3$)

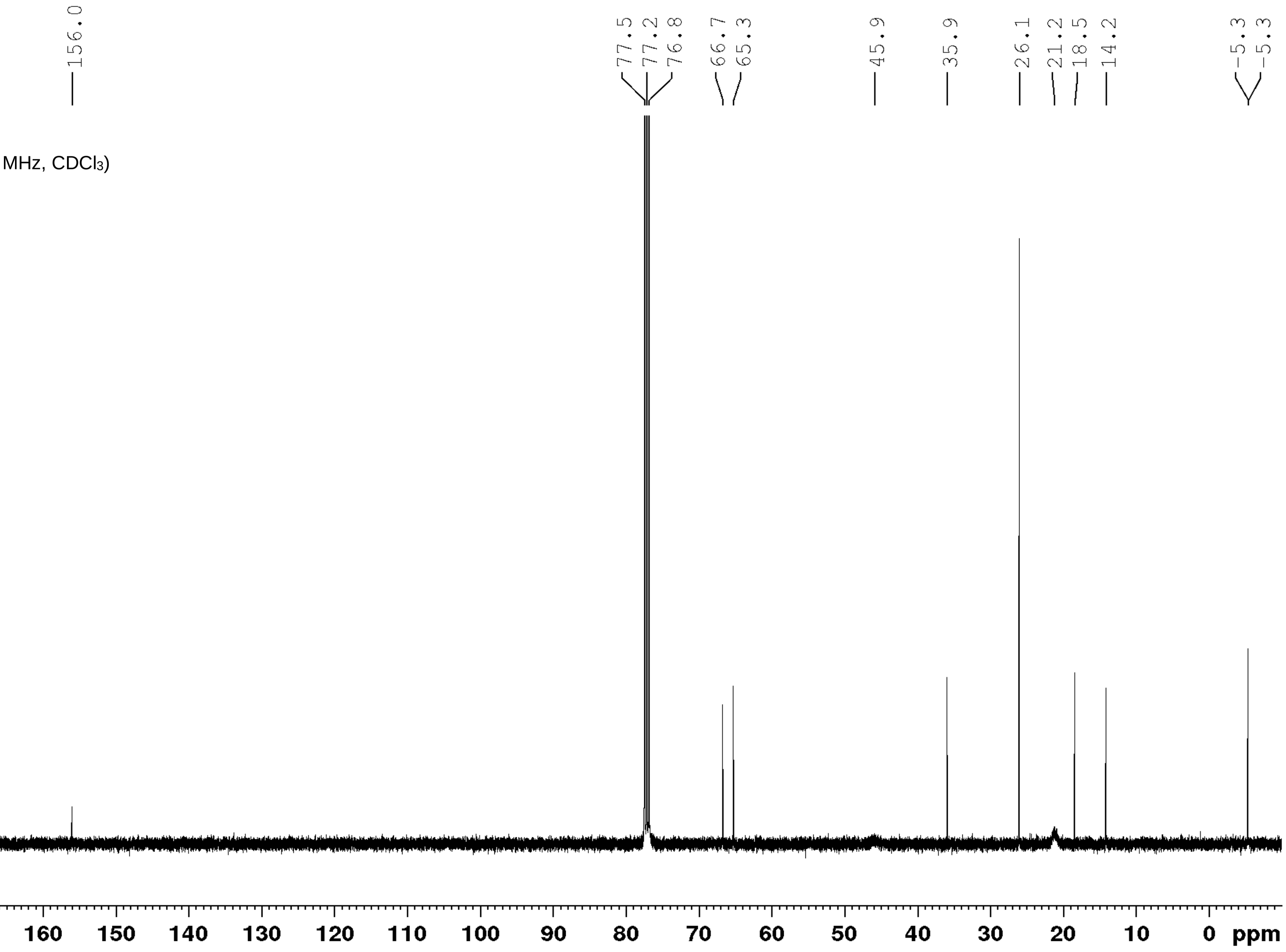

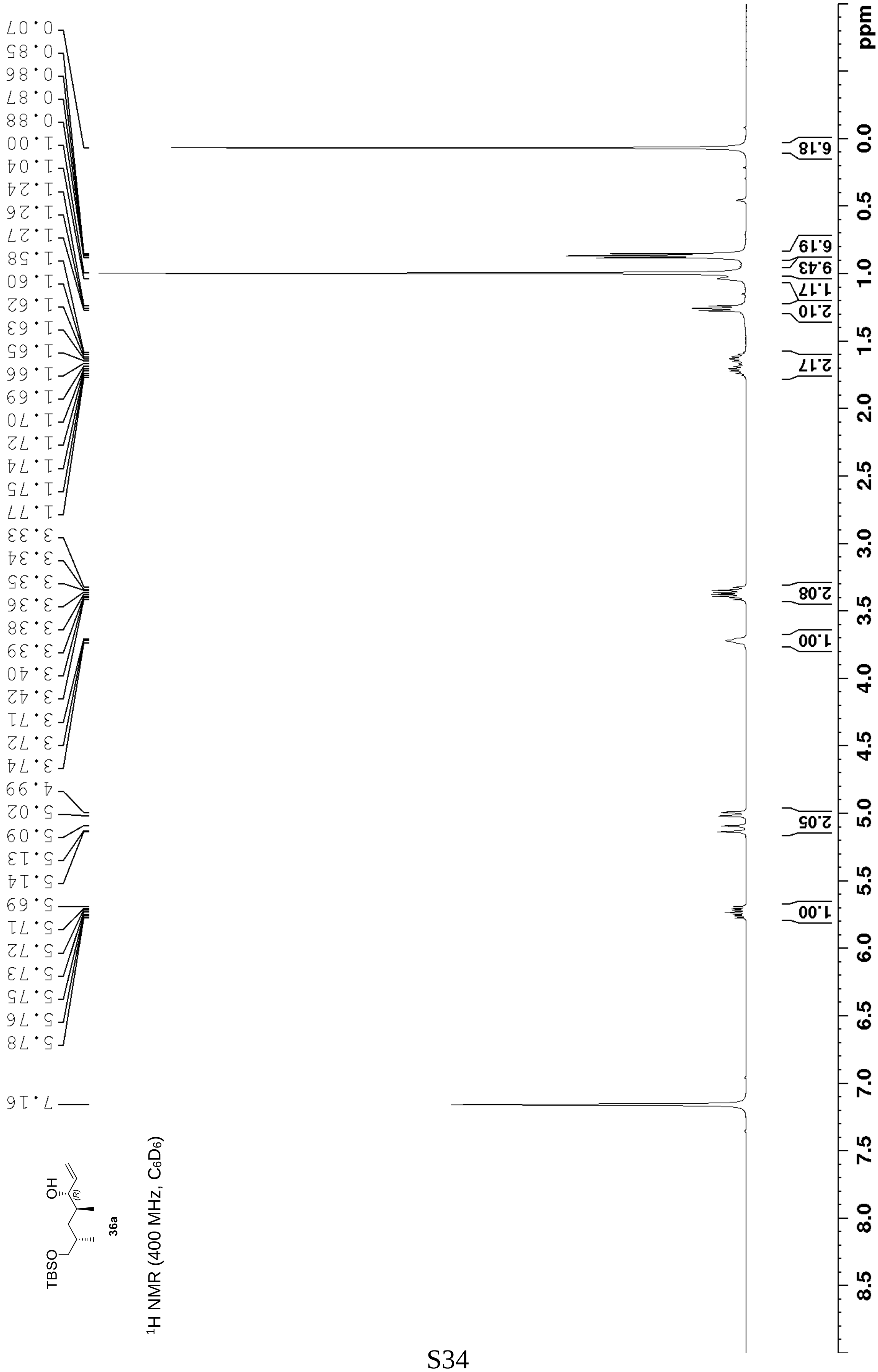
TBSO
OH
(R)
36a
1H NMR (400 MHz, C6D6)
ppm

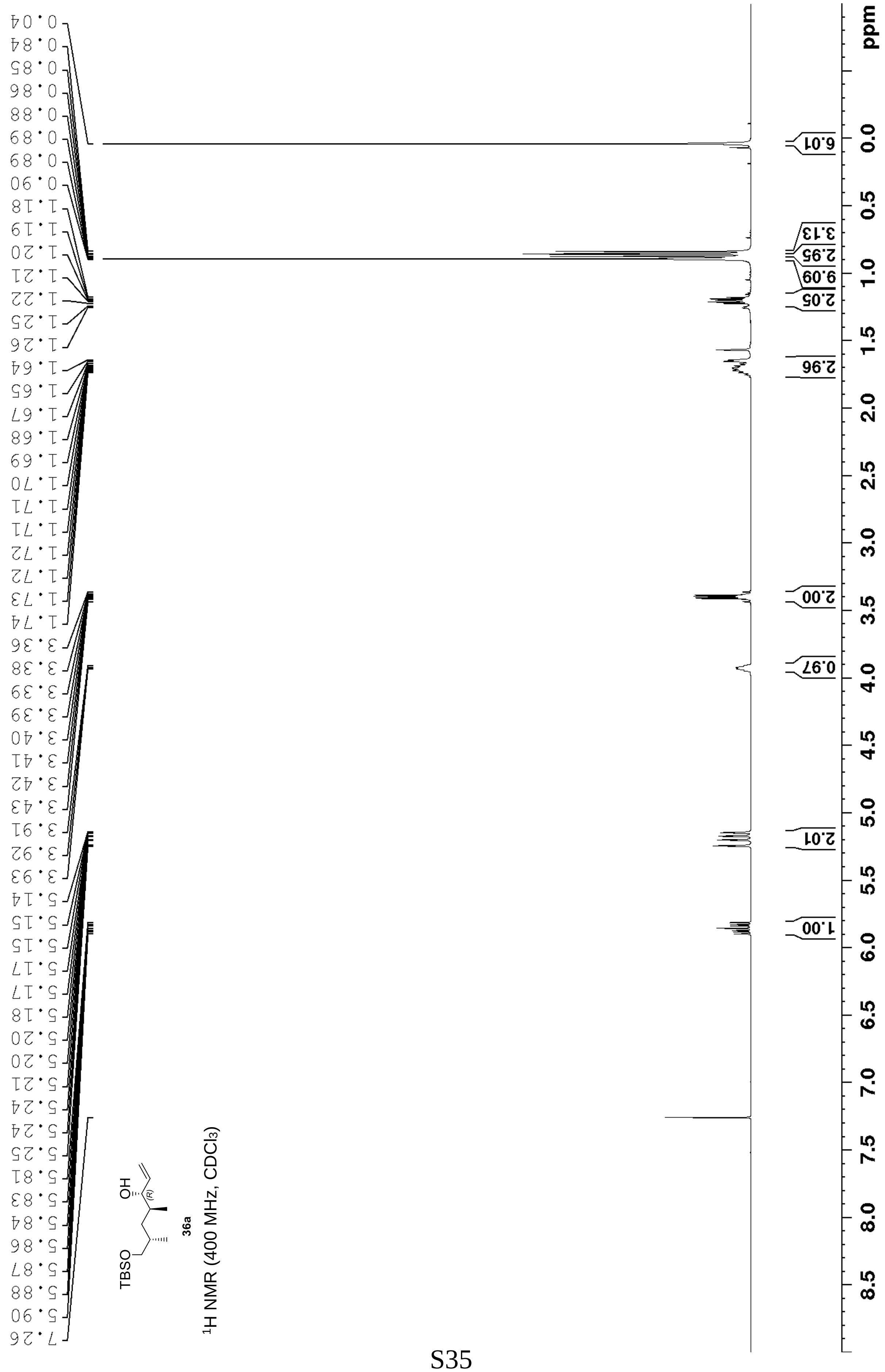
0.04
0.84
0.85
0.86
0.88
0.89
0.89
0.90
1.18
1.19
1.20
1.21
1.22
1.25
1.26
1.64
1.65
1.67
1.68
1.69
1.70
1.71
1.71
1.72
1.72
1.73
1.74
3.36
3.38
3.39
3.39
3.40
3.41
3.42
3.43
3.91
3.92
3.93
5.14
5.15
5.15
5.17
5.17
5.18
5.20
5.20
5.21
5.24
5.24
5.25
5.81
5.83
5.84
5.86
5.87
5.88
5.90
7.26
TBSO
OH
(R)
36a
1H NMR (400 MHz, CDCl3)
6.01
3.13
2.95
9.09
2.05
2.96
2.00
0.97
2.01
1.00
ppm

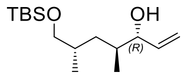

36a

$^{13}C\{^{1}H\}$ NMR (101 MHz, $CDCl_3$)

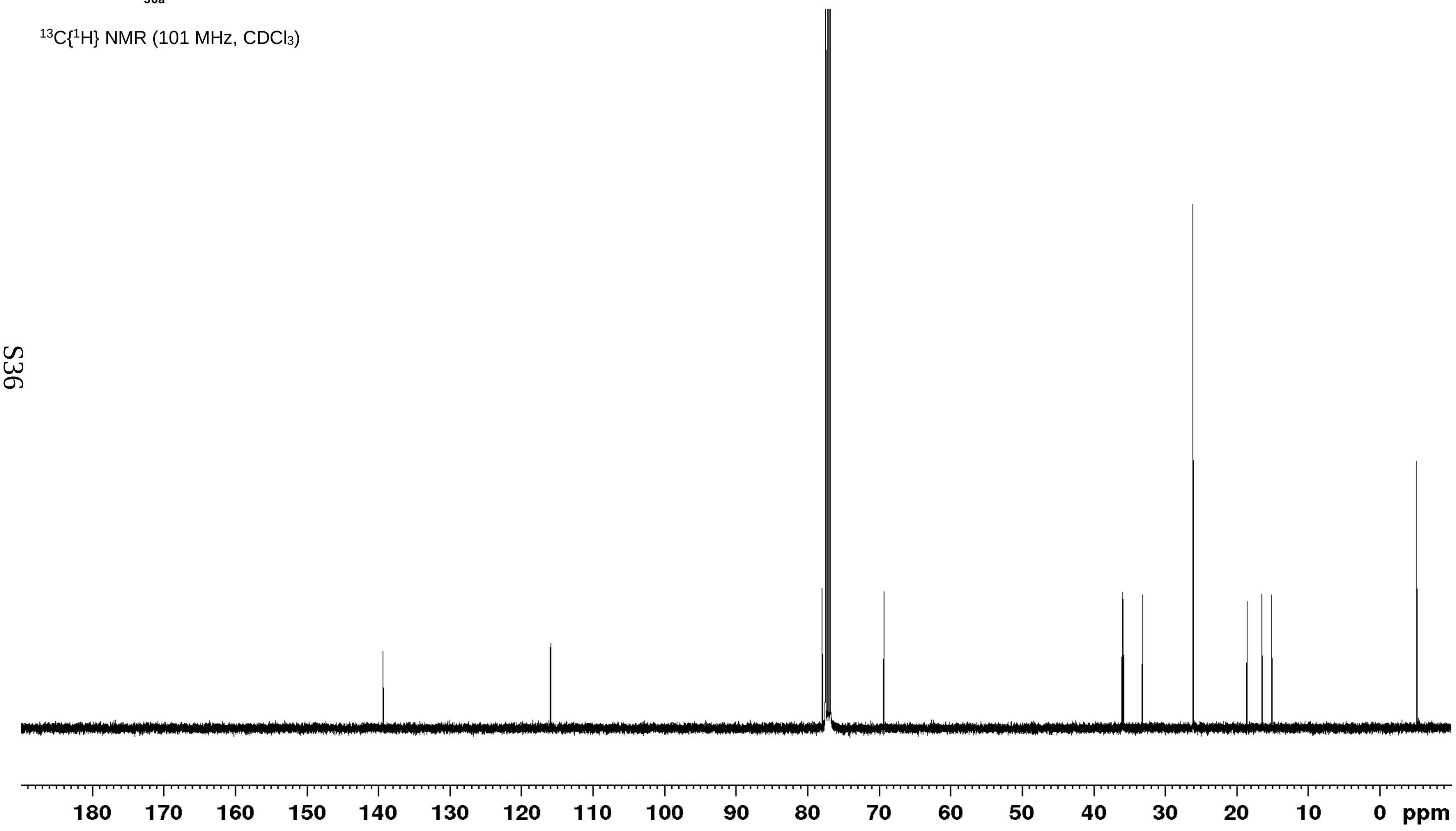

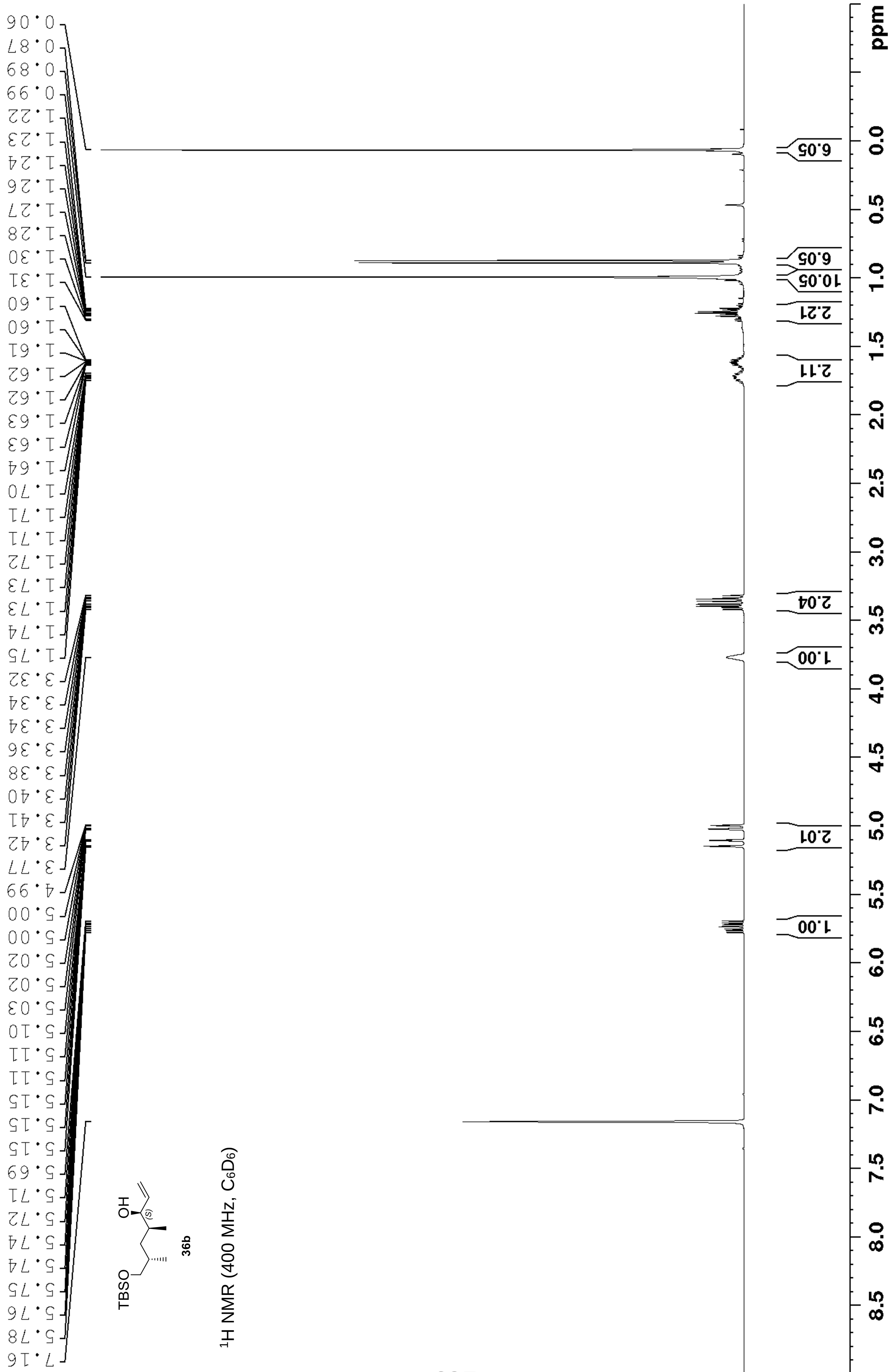
TBSO
OH
36b
1H NMR (400 MHz, C6D6)
ppm

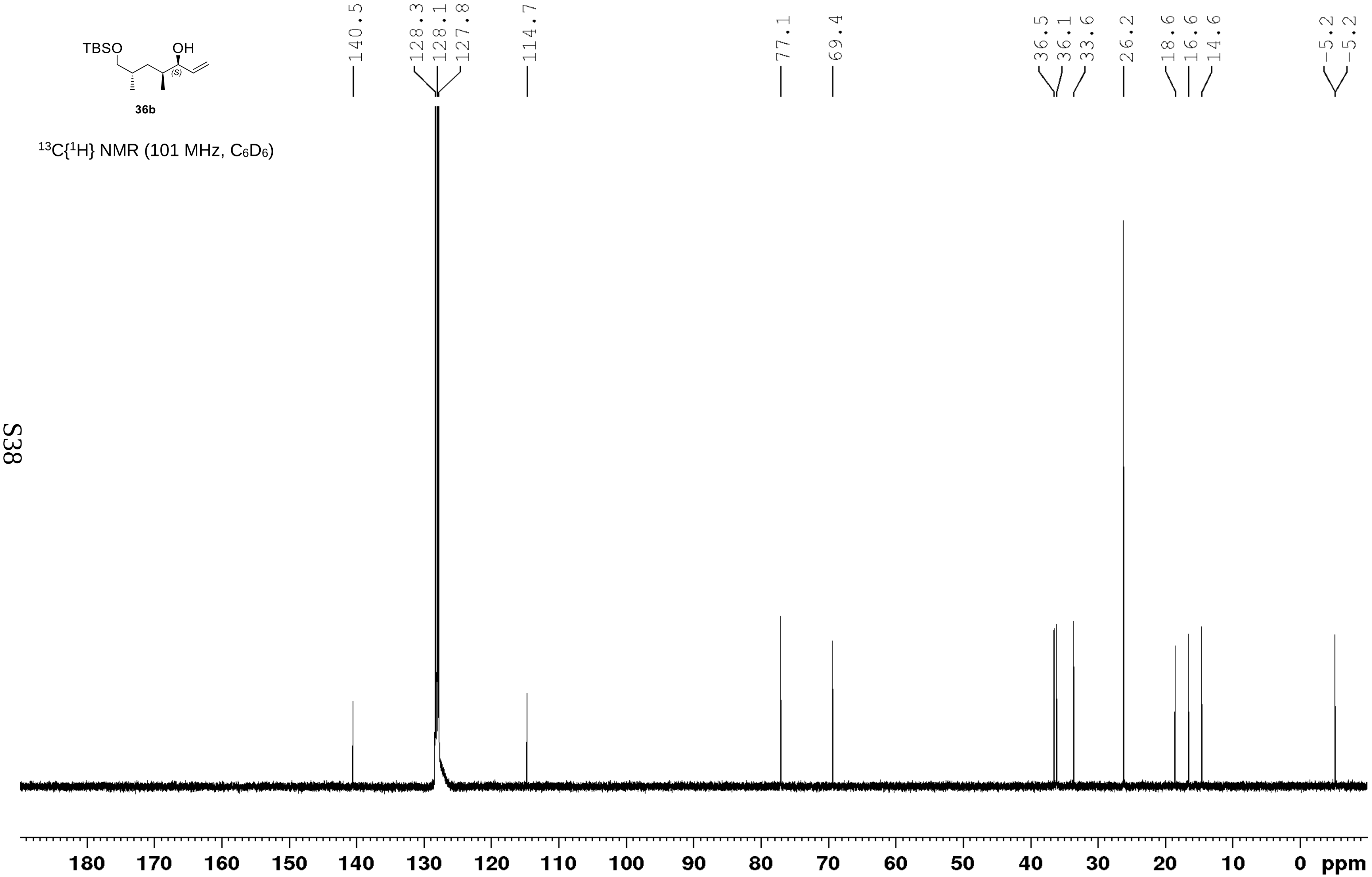

36b

$^{13}C\{^{1}H\}$ NMR (101 MHz, C_6D_6)

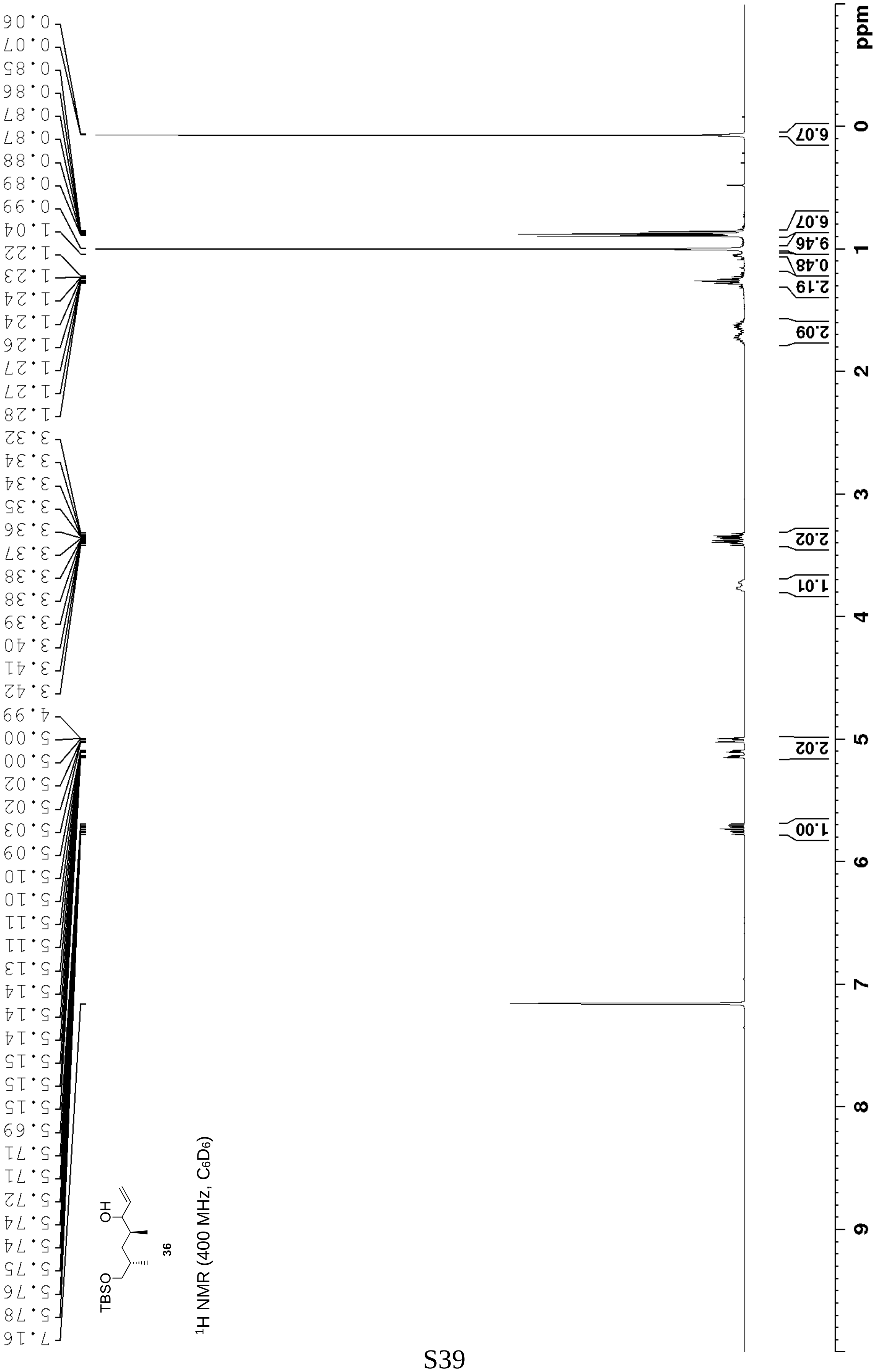

0.06
0.07
0.85
0.86
0.87
0.87
0.88
0.89
0.99
1.04
1.22
1.23
1.24
1.24
1.26
1.27
1.27
1.28
3.32
3.34
3.34
3.35
3.36
3.37
3.38
3.38
3.39
3.40
3.41
3.42
4.99
5.00
5.00
5.02
5.02
5.03
5.09
5.10
5.10
5.11
5.11
5.13
5.14
5.14
5.14
5.15
5.15
5.15
5.69
5.71
5.71
5.72
5.74
5.74
5.75
5.76
5.78
7.16
TBSO
OH
36
1H NMR (400 MHz, C6D6)
6.07
6.07
9.46
0.48
2.19
2.09
2.02
1.01
2.02
1.00
ppm

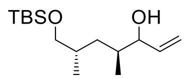

36

$^{13}C\{^1H\}$ NMR (101 MHz, C_6D_6)

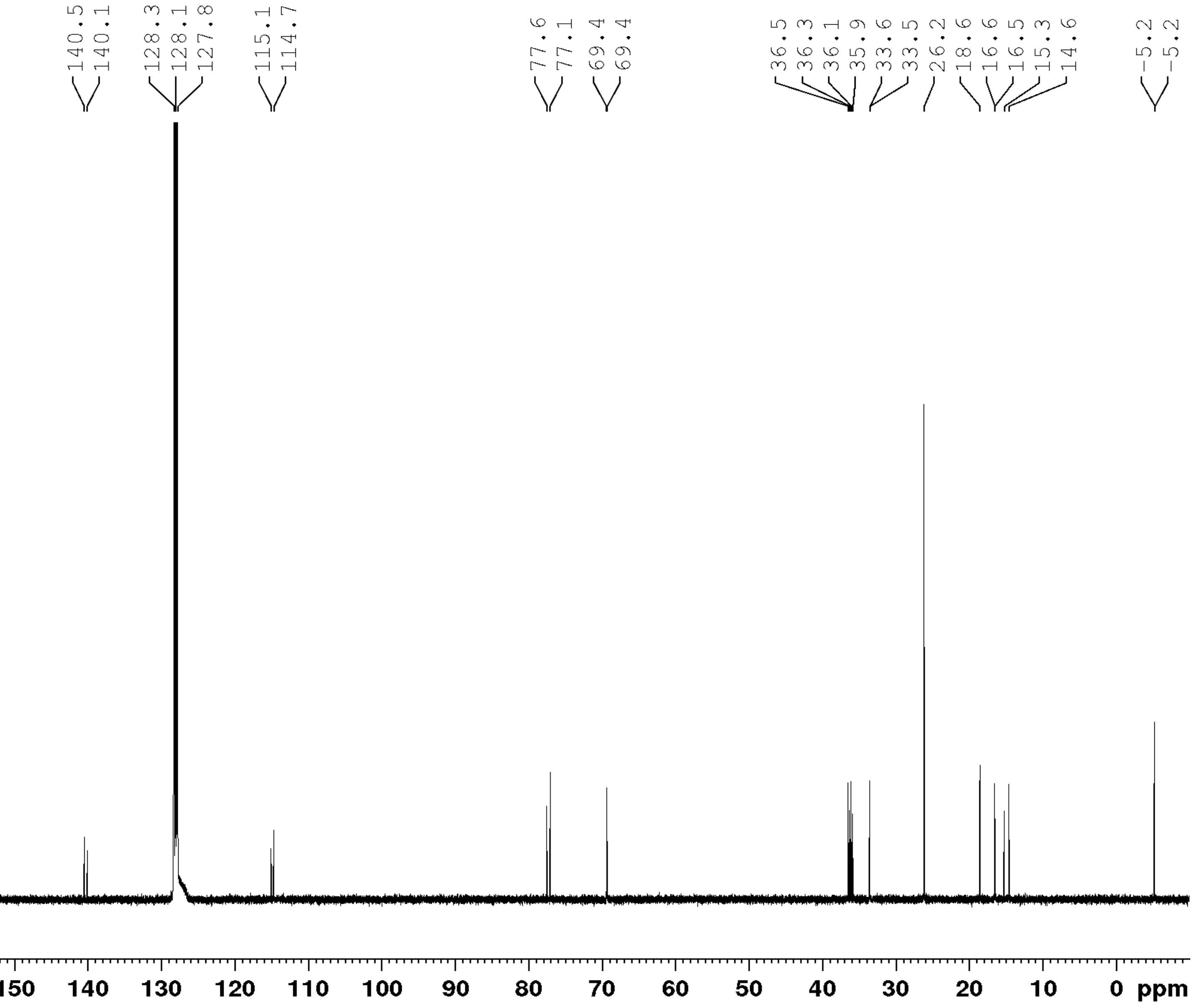

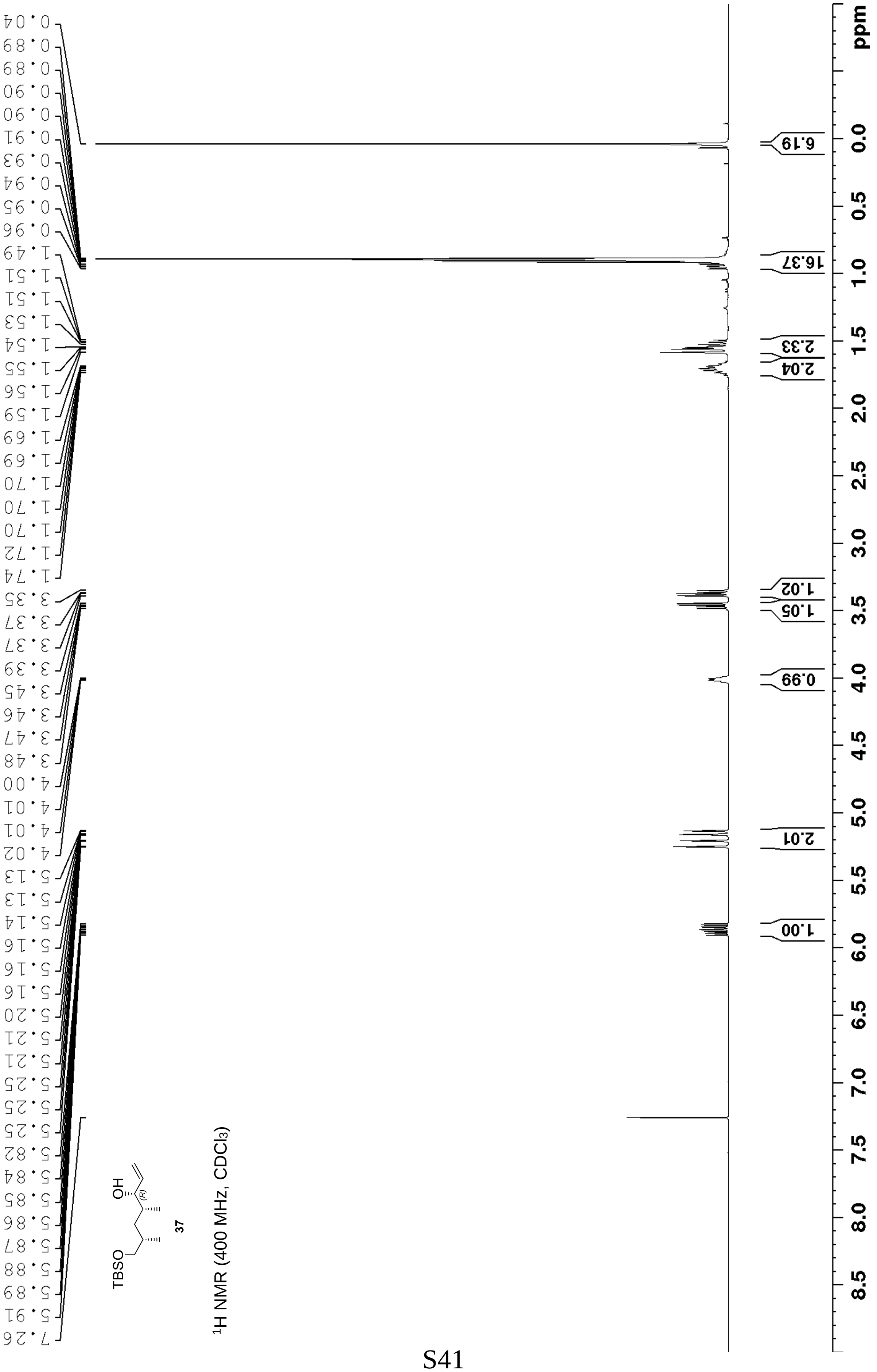
TBSO
OH
(R)
37
1H NMR (400 MHz, CDCl3)
ppm

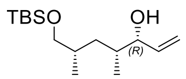

37

$^{13}C\{^{1}H\}$ NMR (101 MHz, $CDCl_3$)

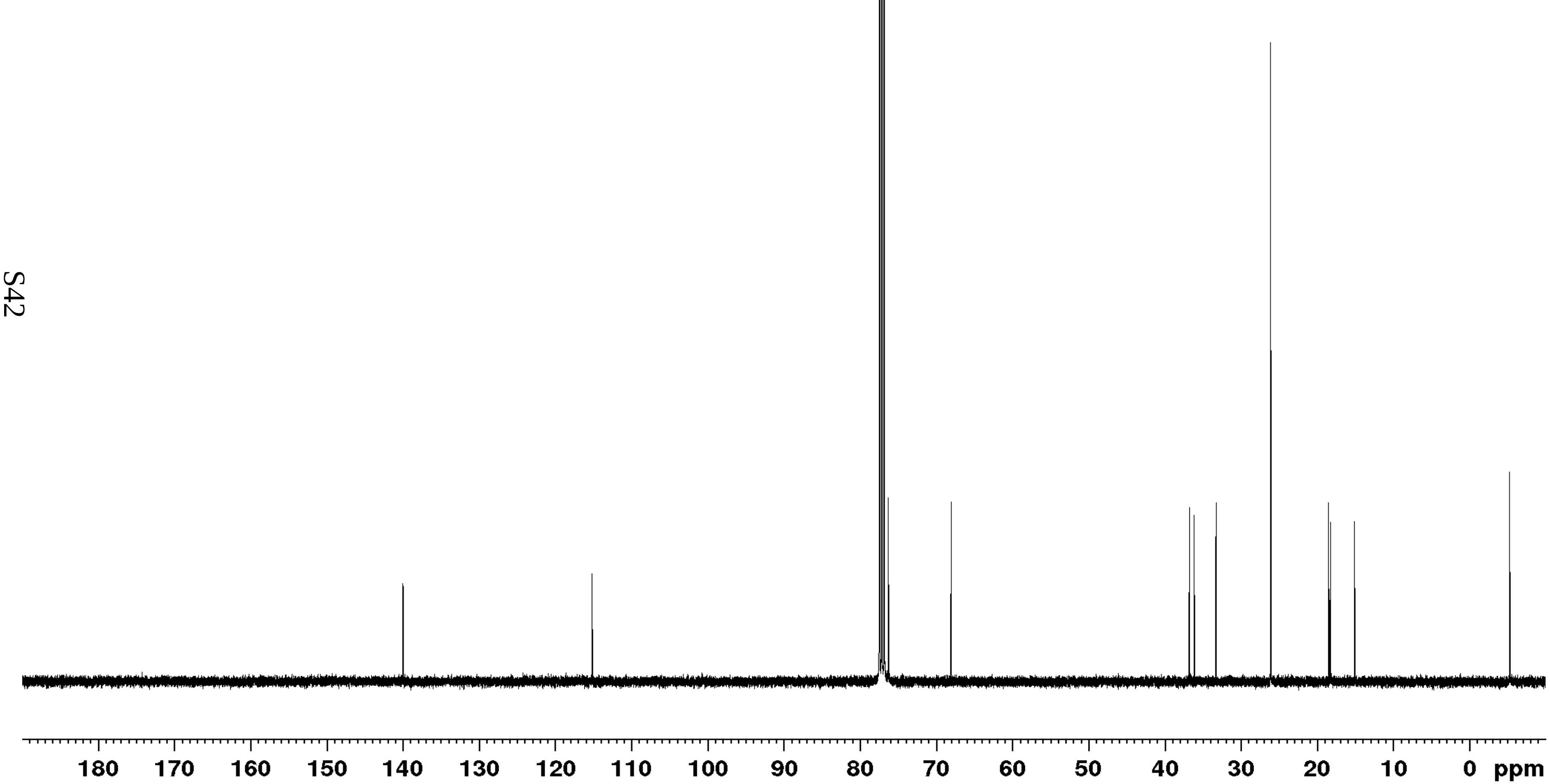

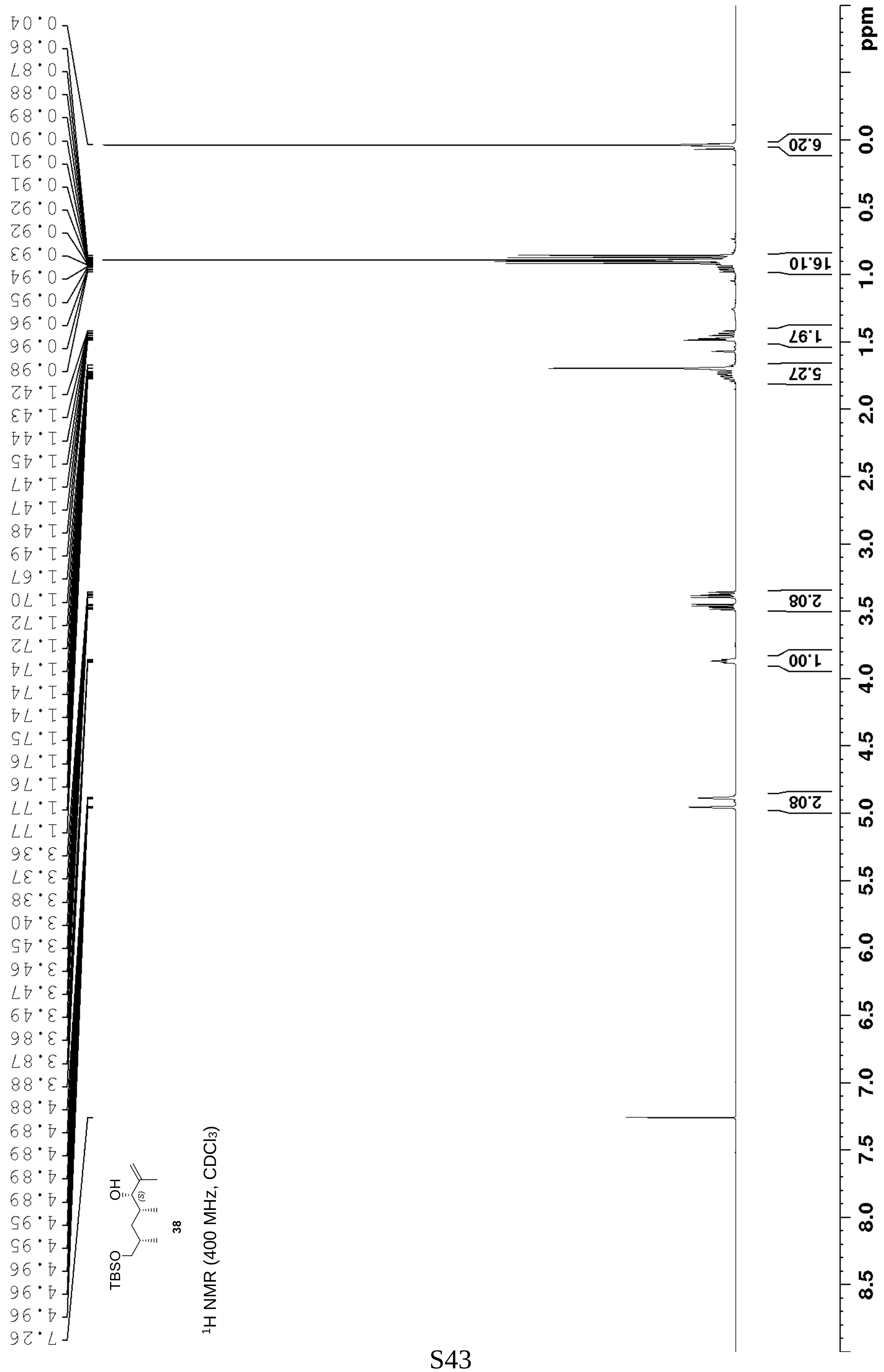

0.04
0.86
0.87
0.88
0.89
0.90
0.91
0.91
0.92
0.92
0.93
0.94
0.95
0.96
0.96
0.98
1.42
1.43
1.44
1.45
1.47
1.47
1.48
1.49
1.67
1.70
1.72
1.72
1.74
1.74
1.74
1.75
1.76
1.76
1.77
1.77
3.36
3.37
3.38
3.40
3.45
3.46
3.47
3.49
3.86
3.87
3.88
4.88
4.89
4.89
4.89
4.89
4.95
4.95
4.96
4.96
4.96
7.26
6.20
16.10
1.97
5.27
2.08
1.00
2.08
ppm
TBSO
OH
(S)
38
1H NMR (400 MHz, CDCl3)

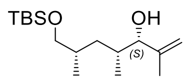

38

$^{13}C\{^{1}H\}$ NMR (101 MHz, $CDCl_3$)

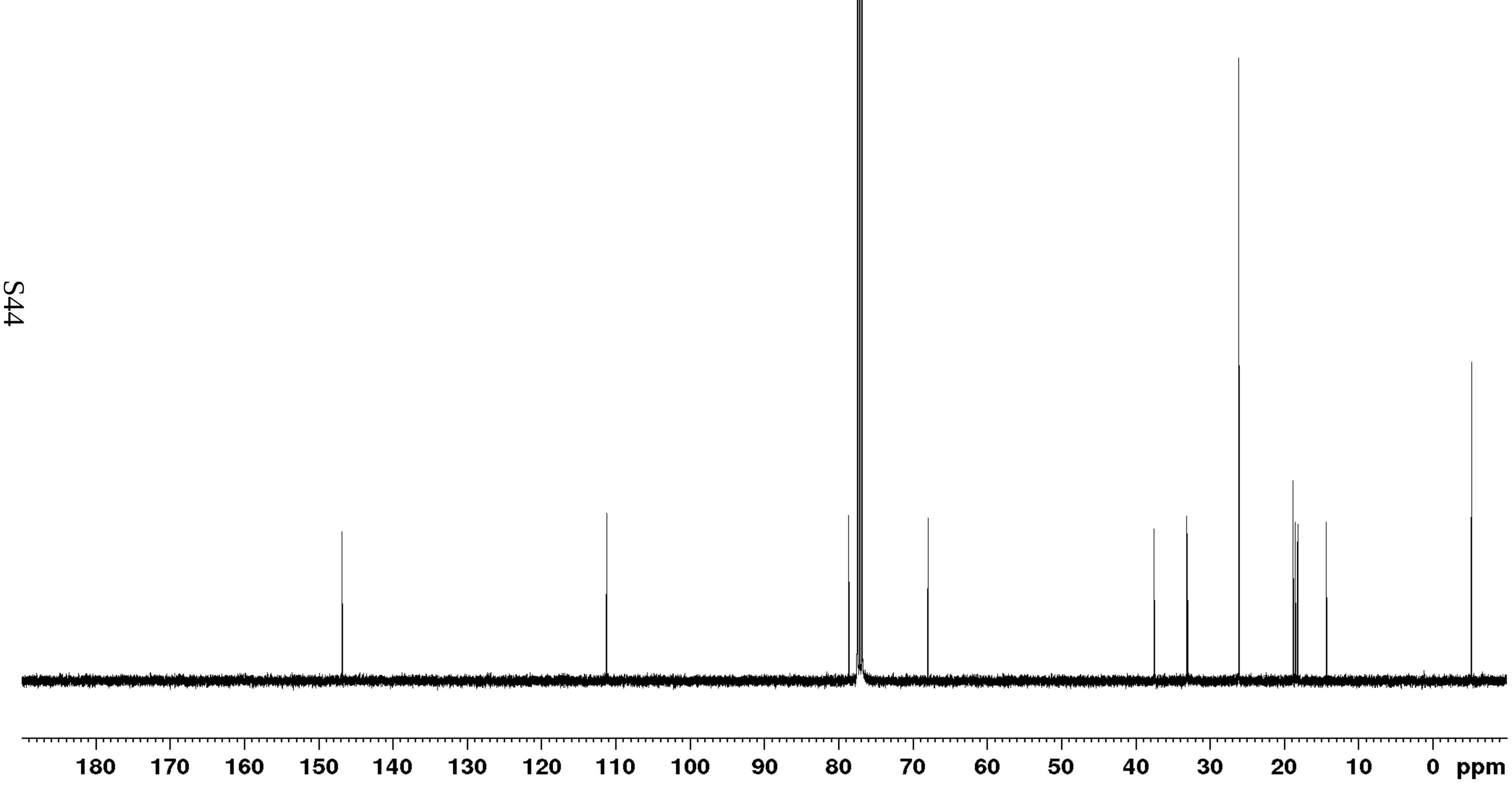

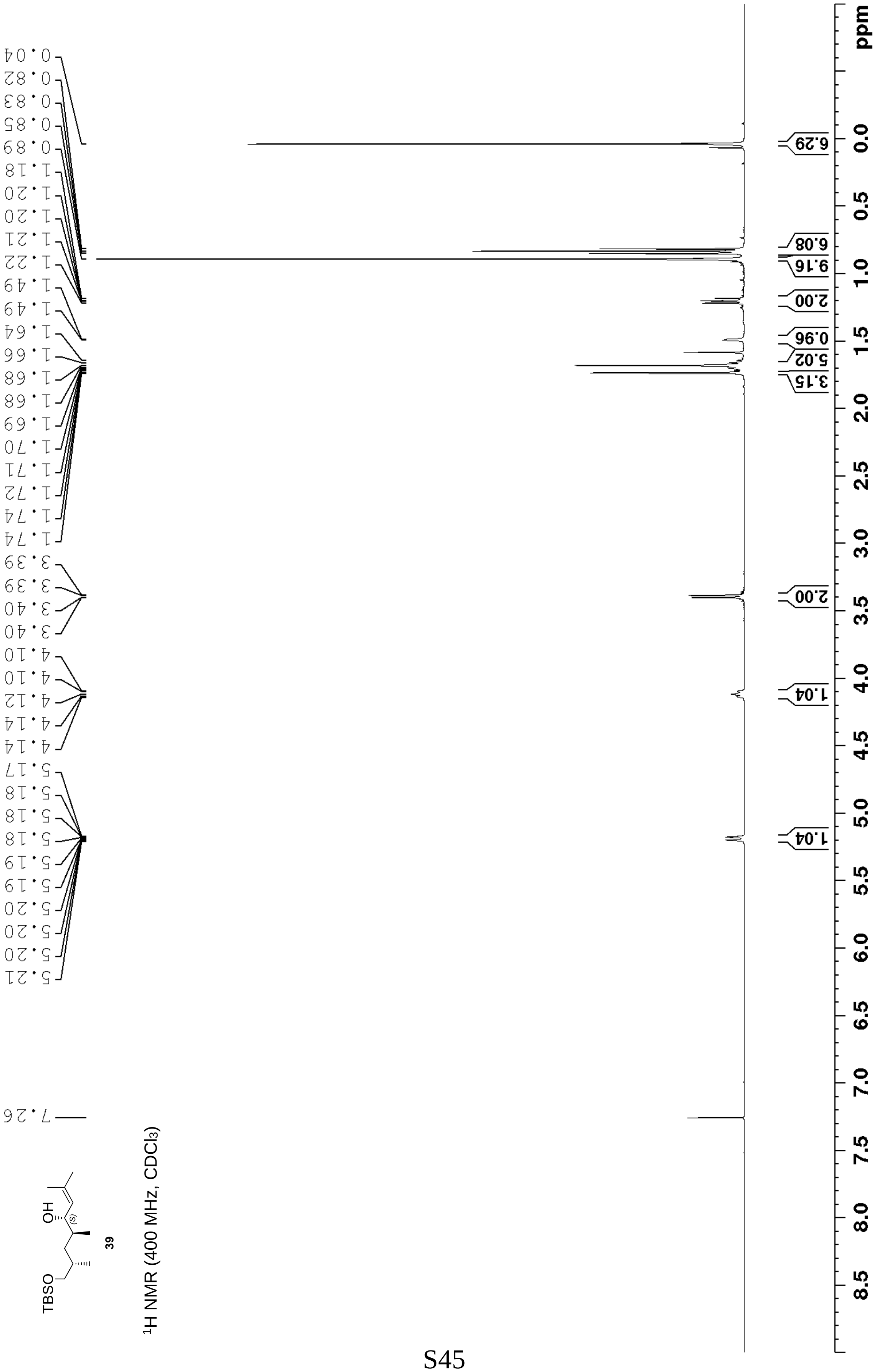
TBSO
OH
(S)
39
1H NMR (400 MHz, CDCl3)
7.26
5.21
5.20
5.20
5.20
5.19
5.19
5.18
5.18
5.18
5.17
4.14
4.14
4.12
4.10
4.10
3.40
3.40
3.39
3.39
1.74
1.74
1.72
1.71
1.70
1.69
1.68
1.68
1.66
1.64
1.49
1.49
1.22
1.21
1.20
1.20
1.18
0.89
0.85
0.83
0.82
0.04
6.29
6.08
9.16
2.00
0.96
5.02
3.15
2.00
1.04
1.04
ppm

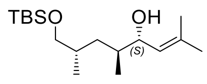

39

$^{13}C\{^{1}H\}$ NMR (101 MHz, $CDCl_3$)

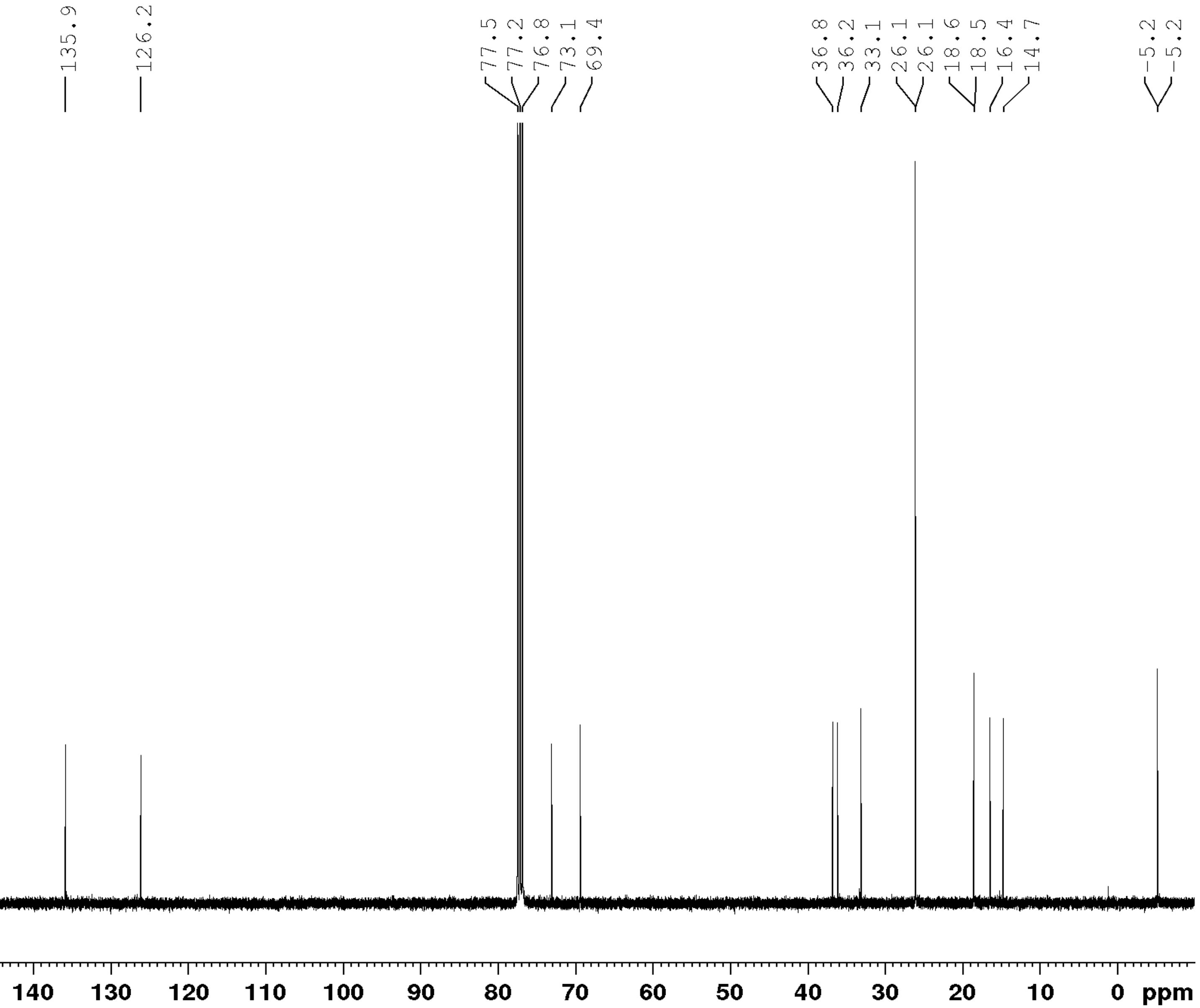

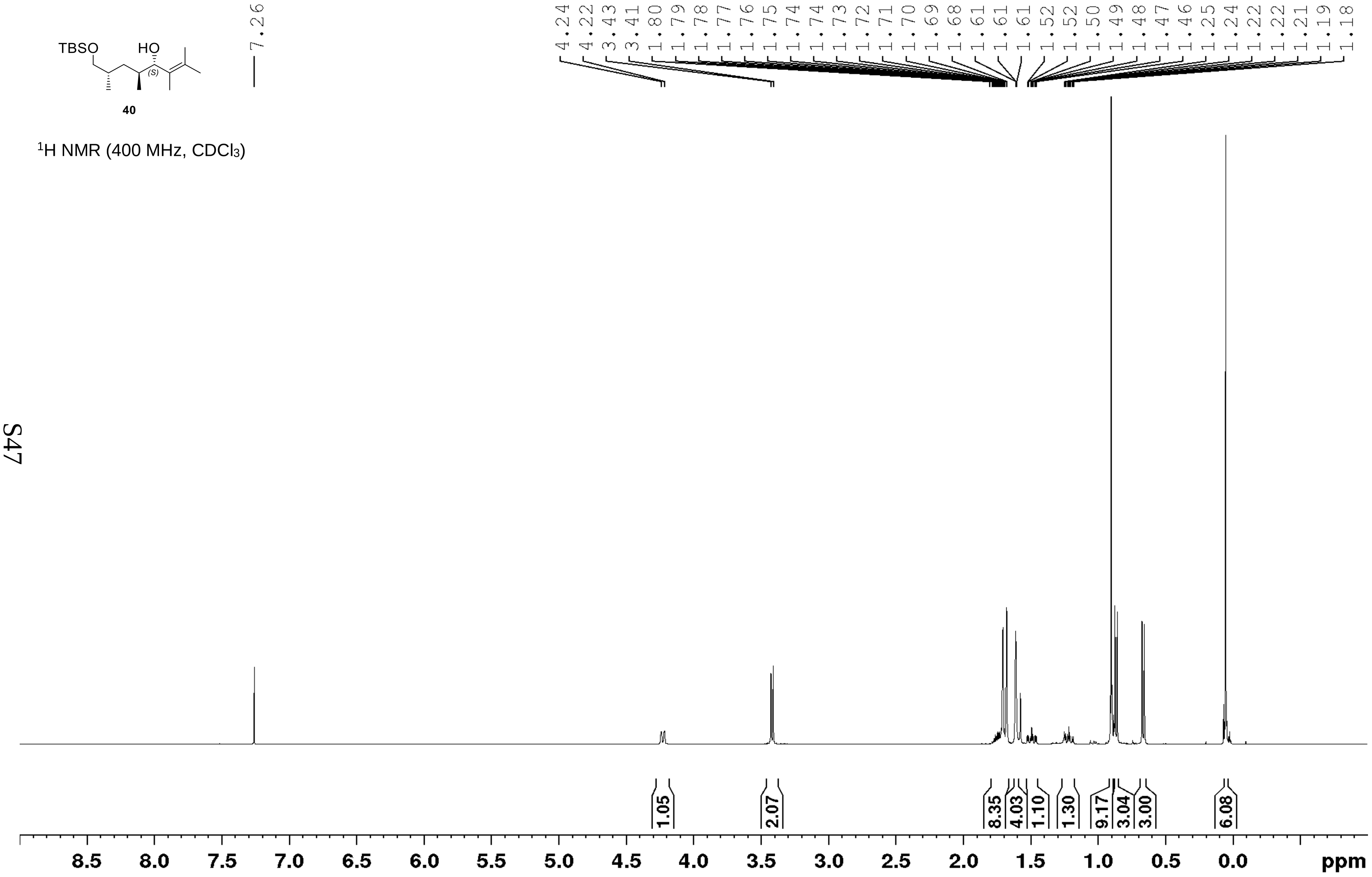

40

^{1}H NMR (400 MHz, $CDCl_3$)

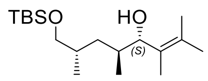

40

$^{13}C\{^{1}H\}$ NMR (101 MHz, $CDCl_3$)

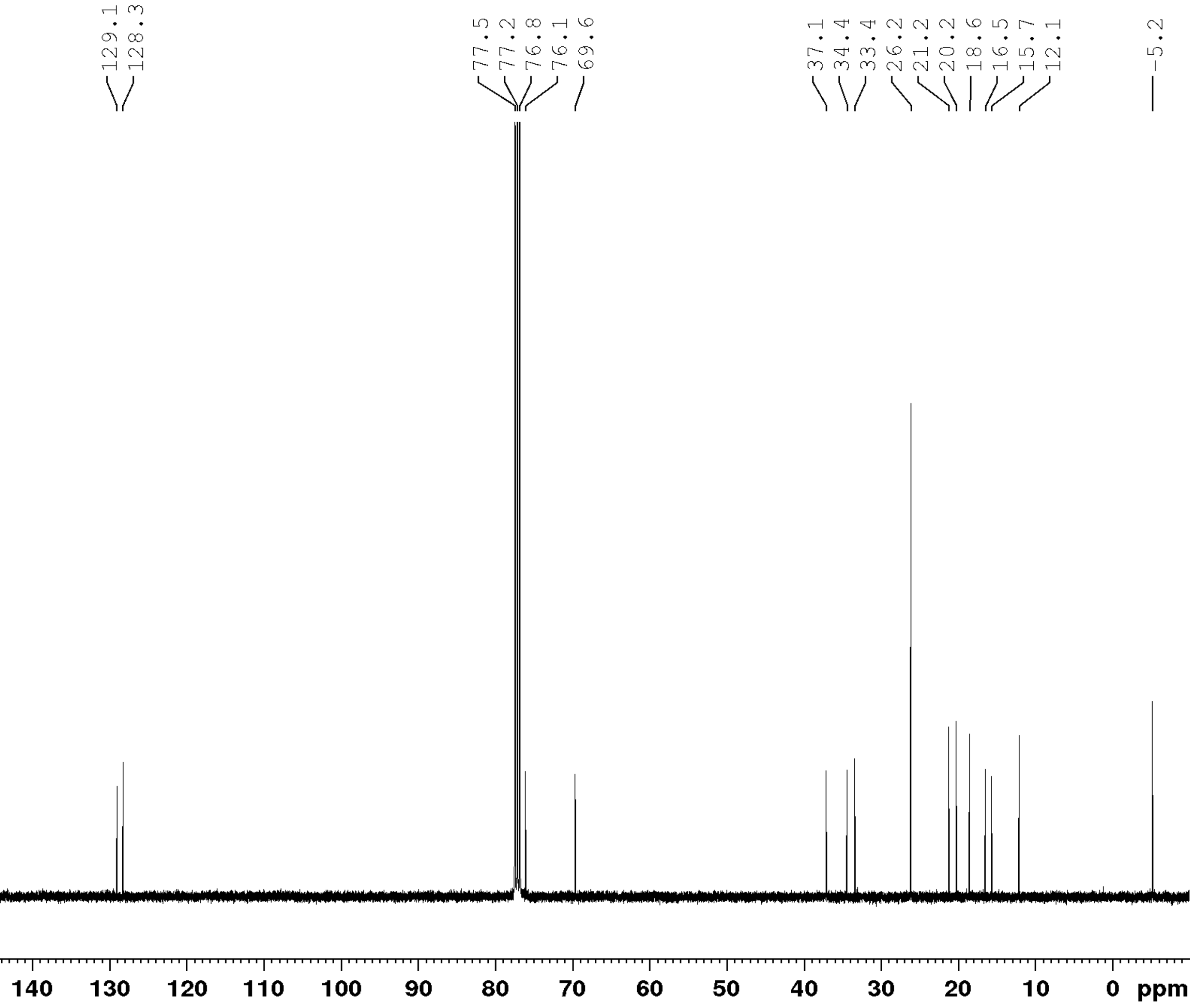

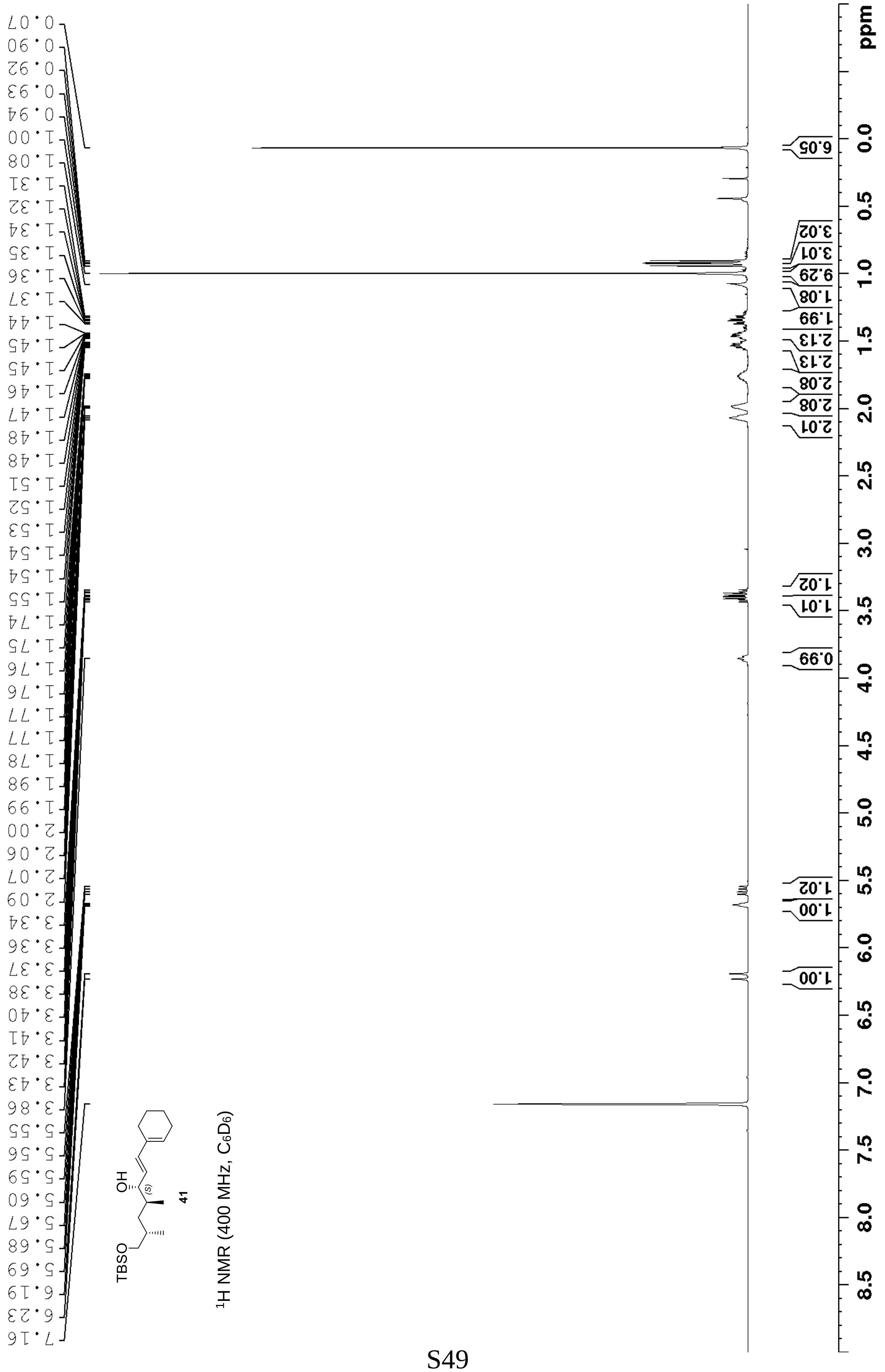

41

^{1}H NMR (400 MHz, C_6D_6)

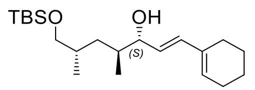

41

$^{13}C\{^{1}H\}$ NMR (101 MHz, C_6D_6)

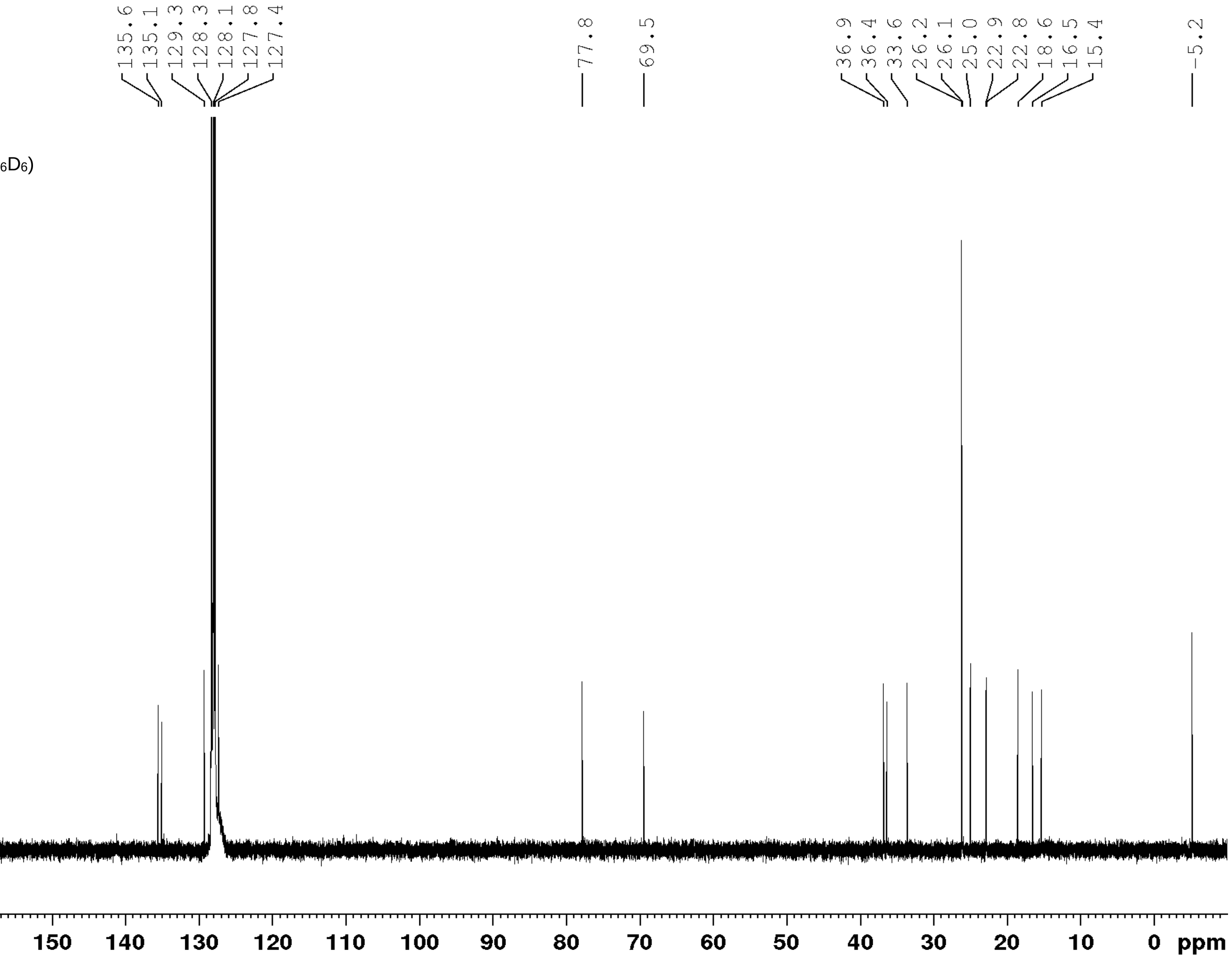

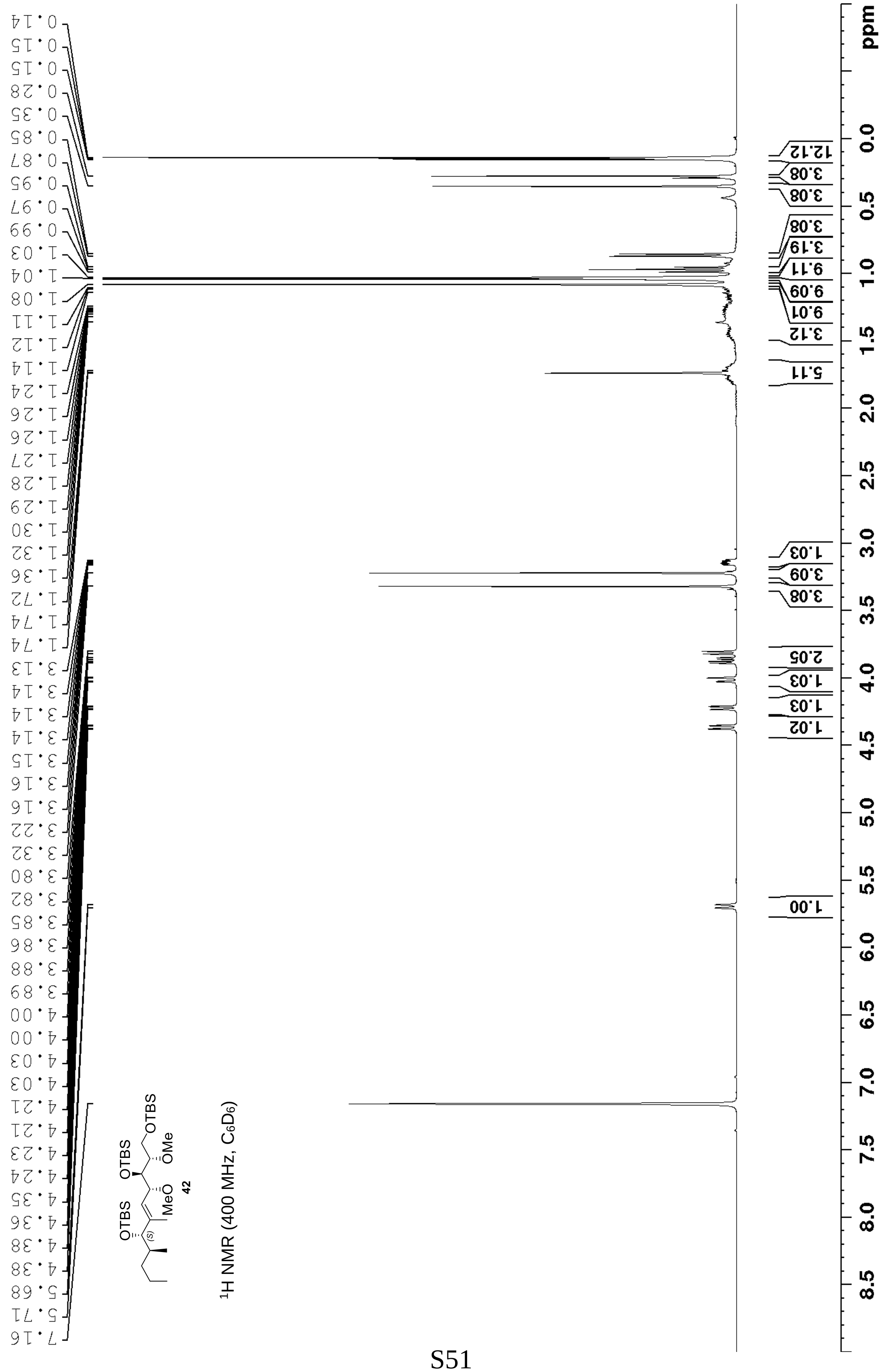
OTBS
OTBS
OTBS
MeO
OMe
42
1H NMR (400 MHz, C6D6)
ppm

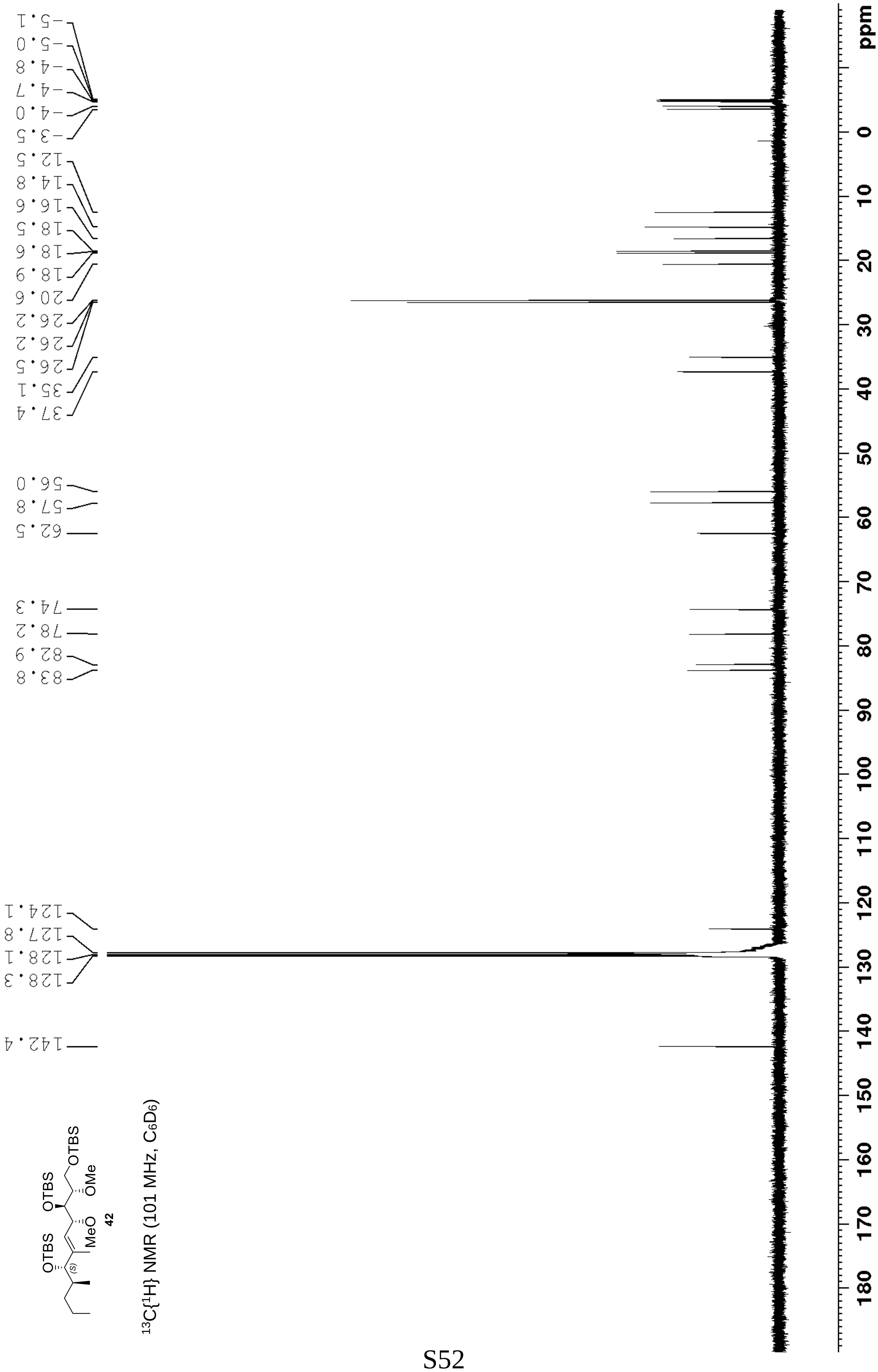

$^{13}C\{^{1}H\}$ NMR (101 MHz, C_6D_6)

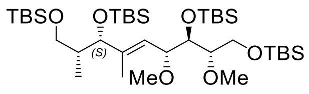

43

^{1}H NMR (400 MHz, C_6D_6)

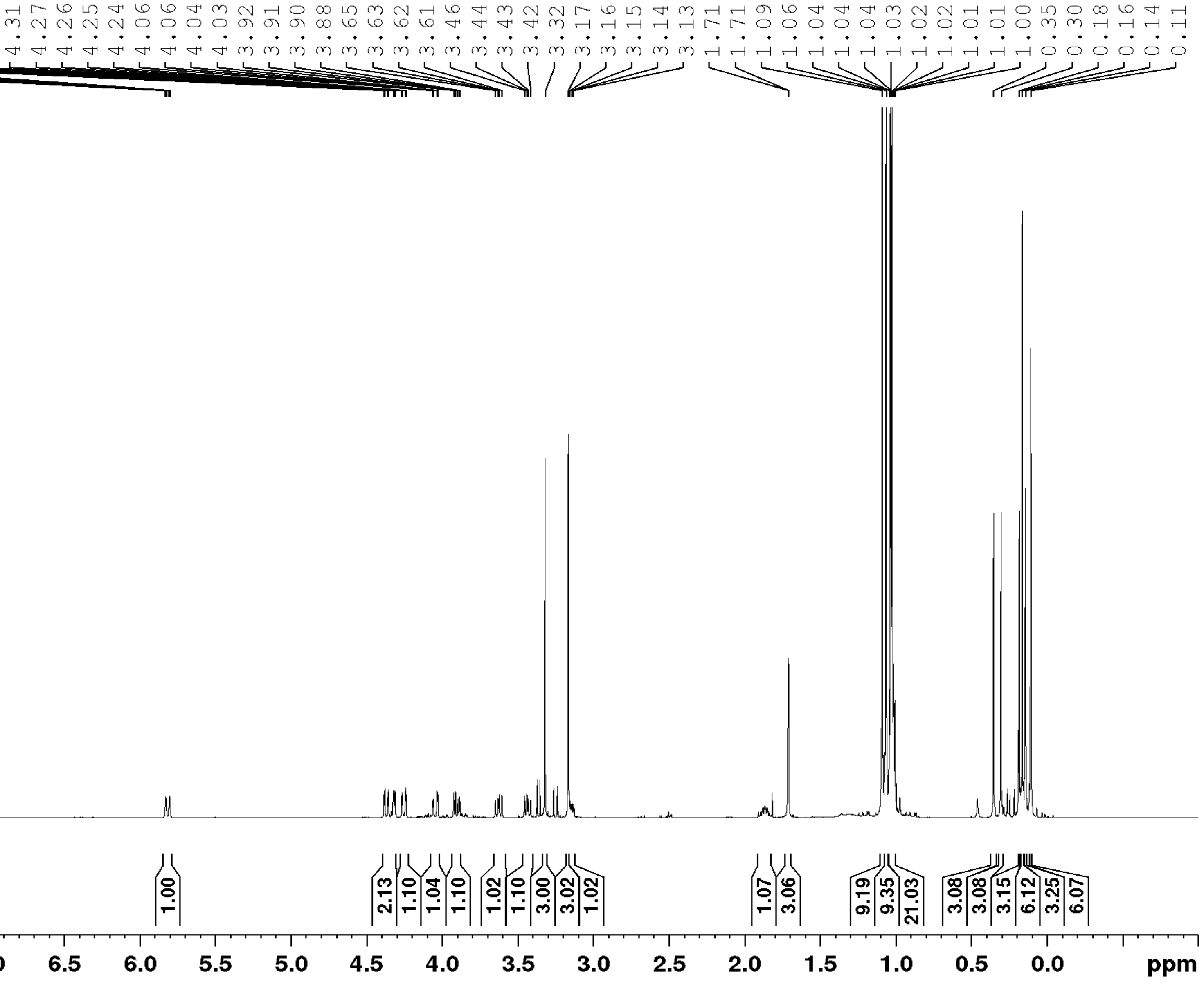

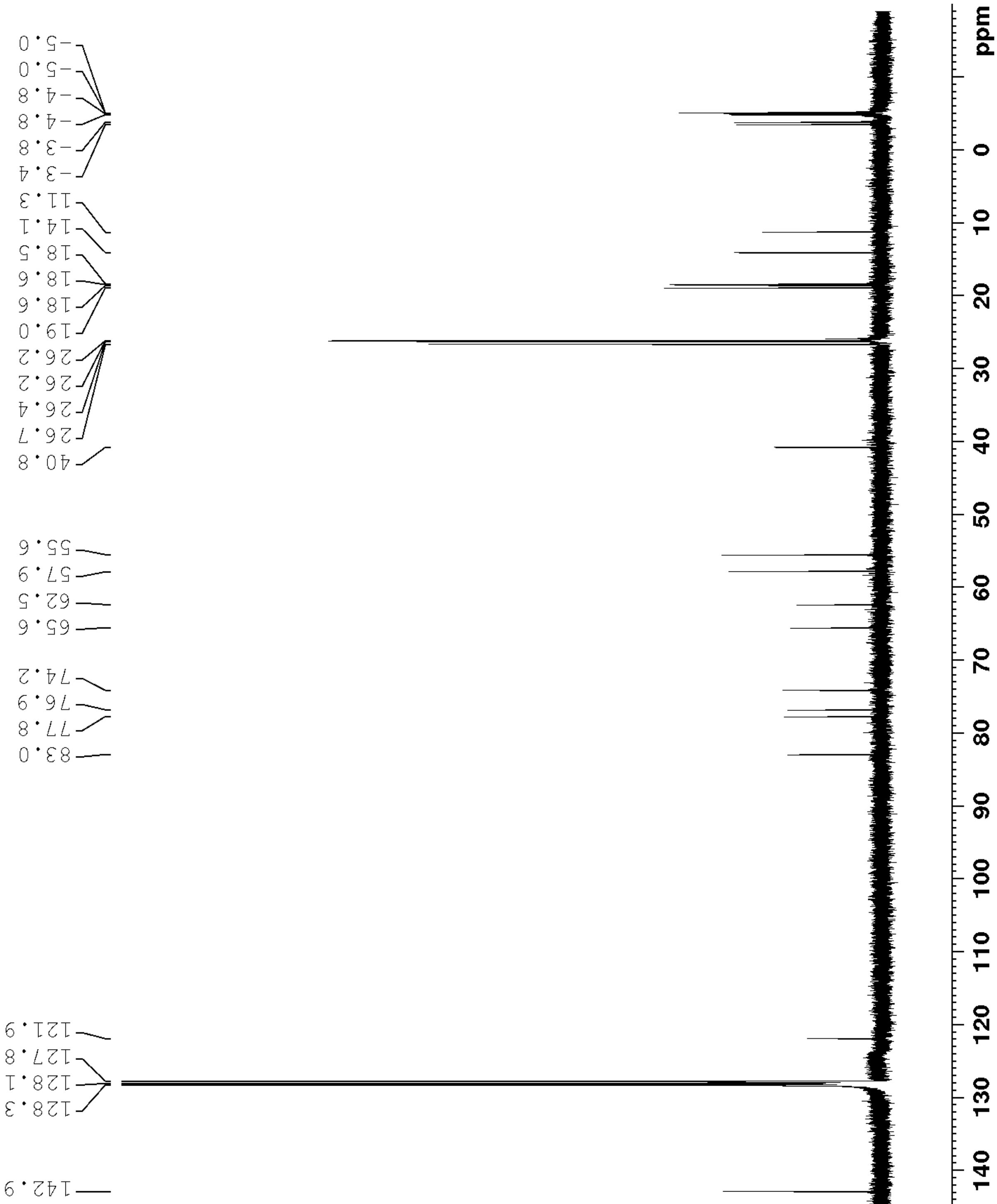

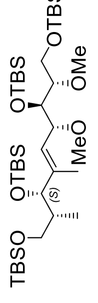

43

$^{13}C\{^{1}H\}$ NMR (101 MHz, C_6D_6)

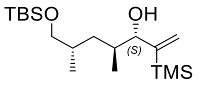

44

^{1}H NMR (400 MHz, C_6D_6)

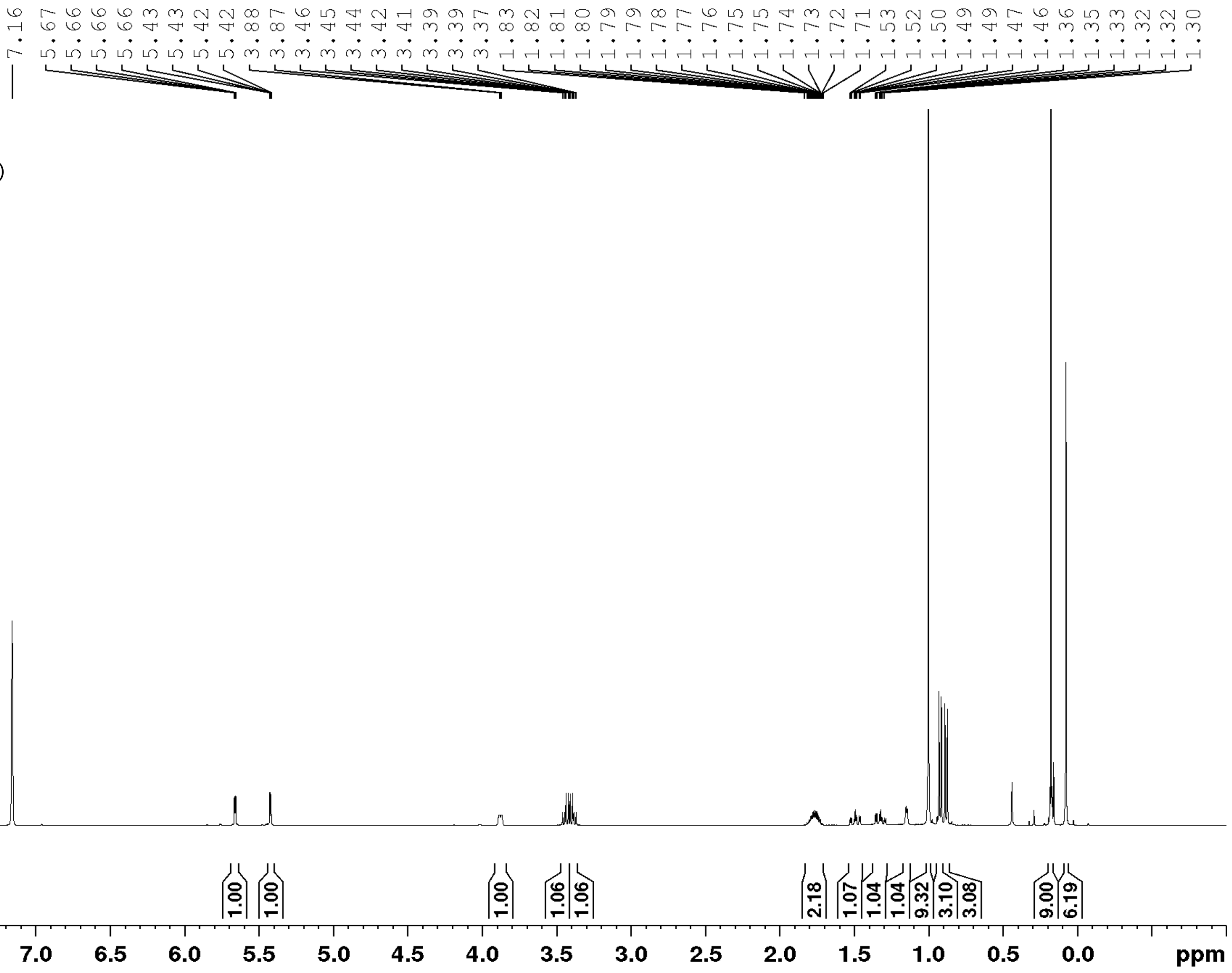

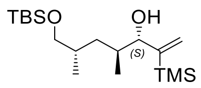

44

$^{13}C\{^1H\}$ NMR (101 MHz, C_6D_6)

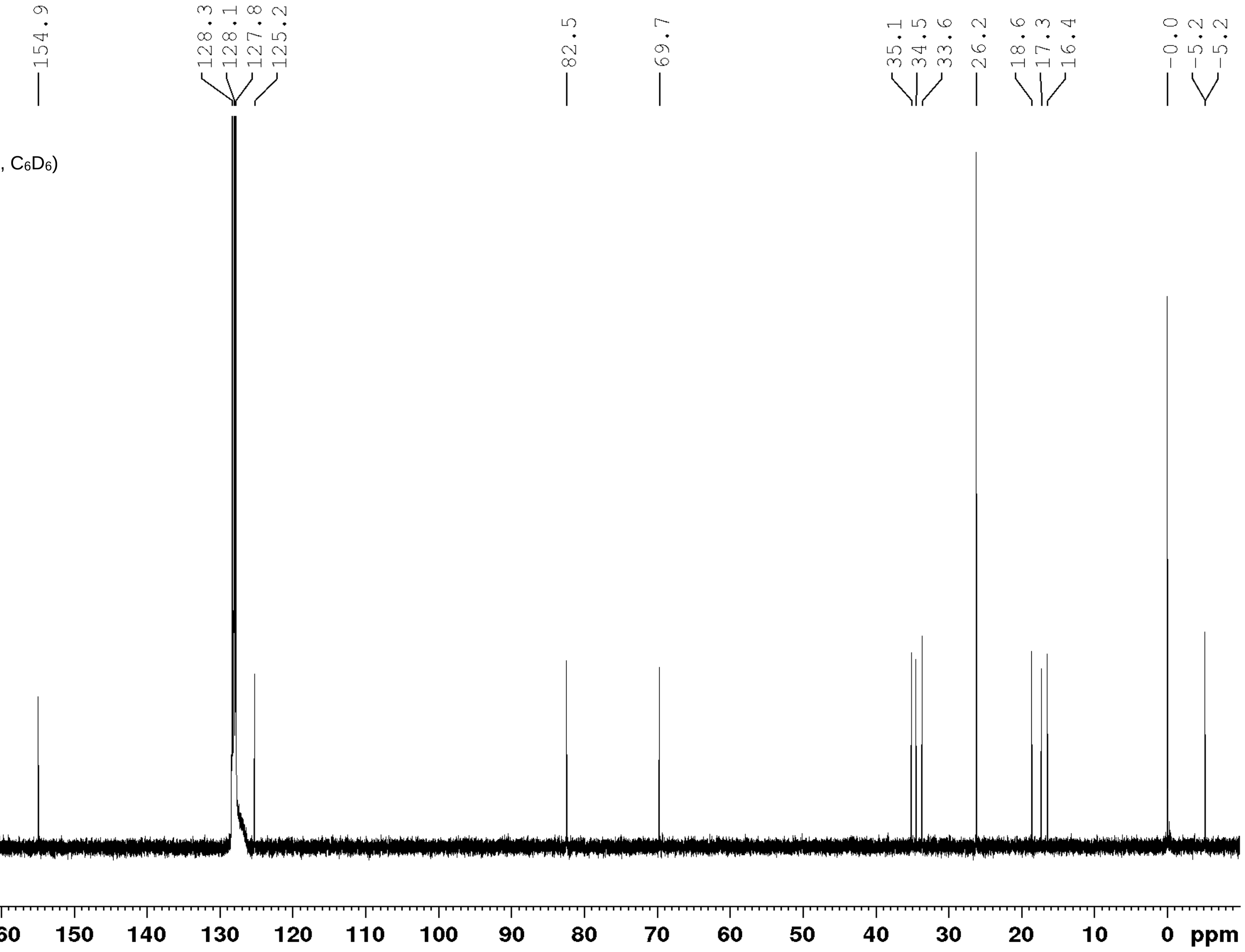

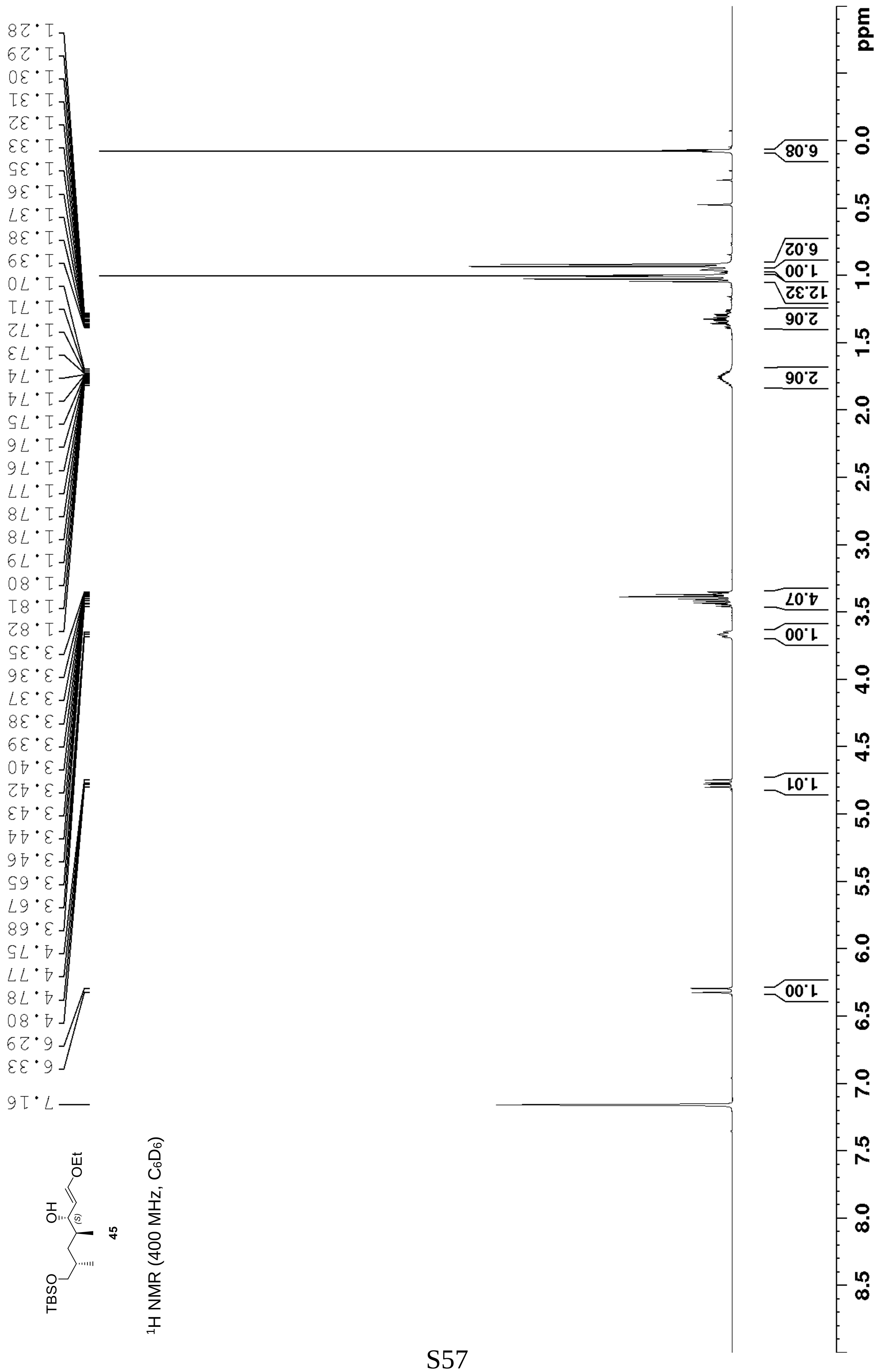
TBSO
OH
(S)
OEt
45
1H NMR (400 MHz, C6D6)
1.28
1.29
1.30
1.31
1.32
1.33
1.35
1.36
1.37
1.38
1.39
1.70
1.71
1.72
1.73
1.74
1.74
1.75
1.76
1.76
1.77
1.78
1.78
1.79
1.80
1.81
1.82
3.35
3.36
3.37
3.38
3.39
3.40
3.42
3.43
3.44
3.46
3.65
3.67
3.68
4.75
4.77
4.78
4.80
6.29
6.33
7.16
6.08
6.02
1.00
12.32
2.06
2.06
4.07
1.00
1.01
1.00
ppm
0.0
0.5
1.0
1.5
2.0
2.5
3.0
3.5
4.0
4.5
5.0
5.5
6.0
6.5
7.0
7.5
8.0
8.5

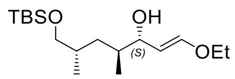

45

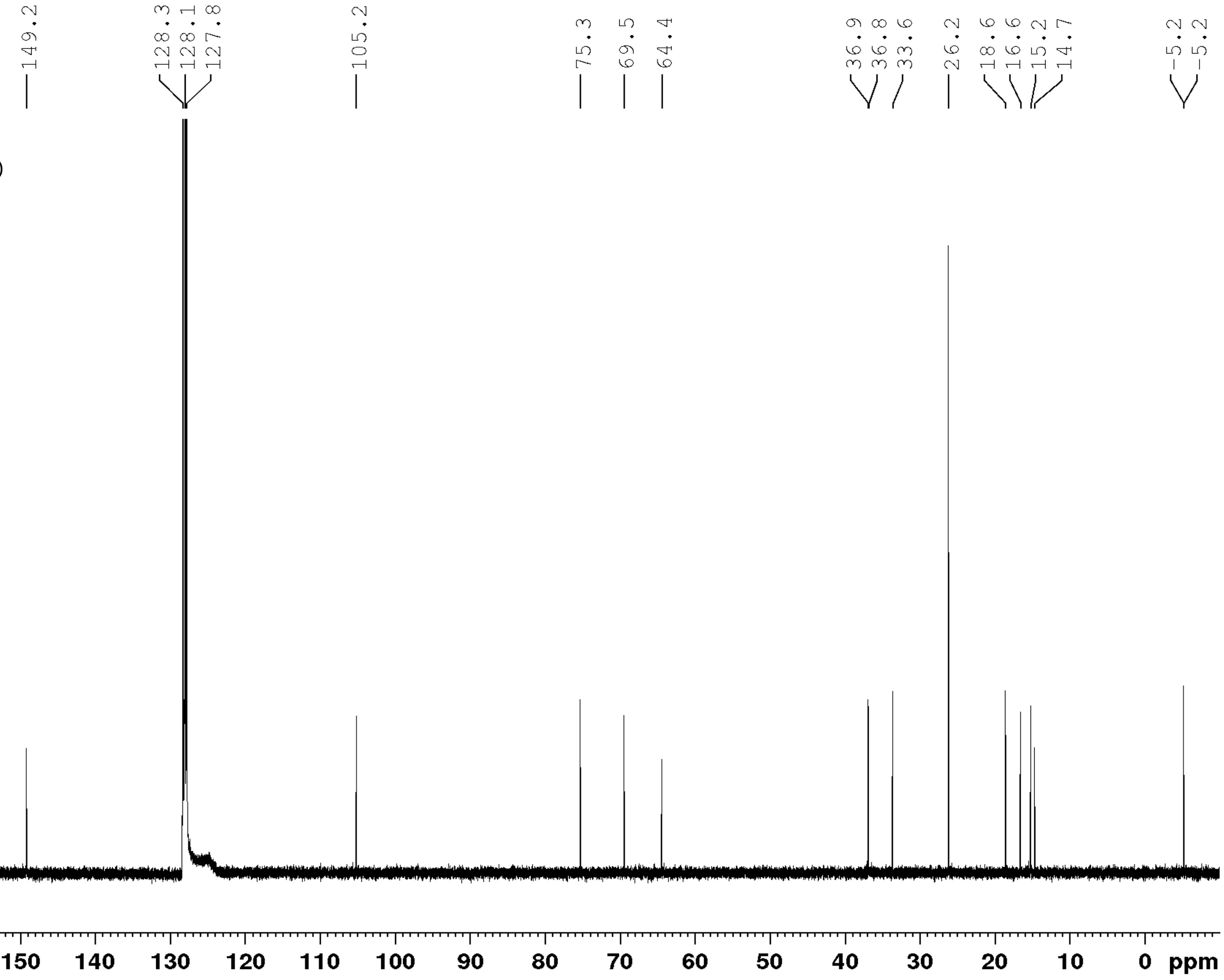

$^{13}C\{^1H\}$ NMR (101 MHz, C_6D_6)

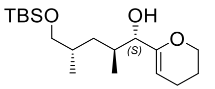

46

^{1}H NMR (400 MHz, C_6D_6)

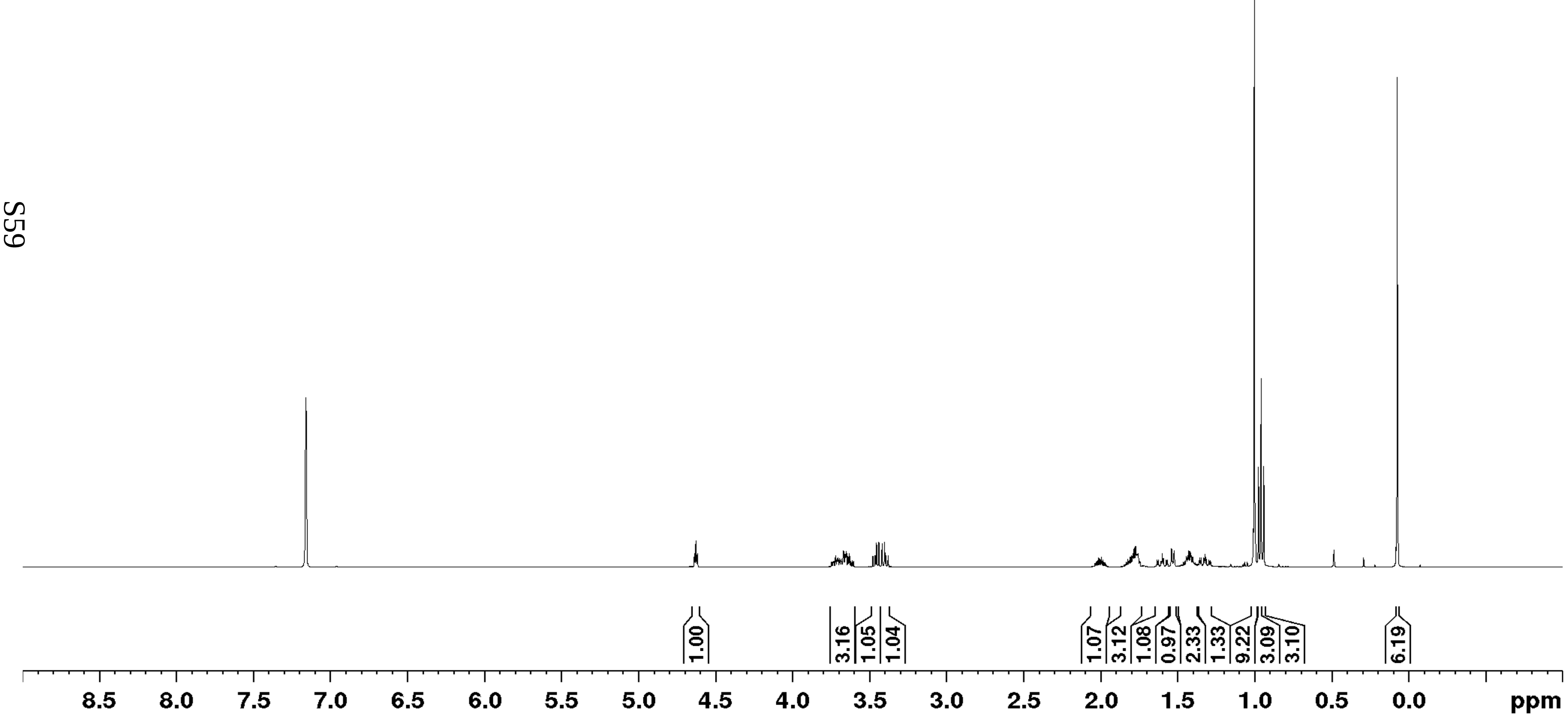

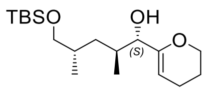

46

$^{13}C\{^1H\}$ NMR (101 MHz, C_6D_6)

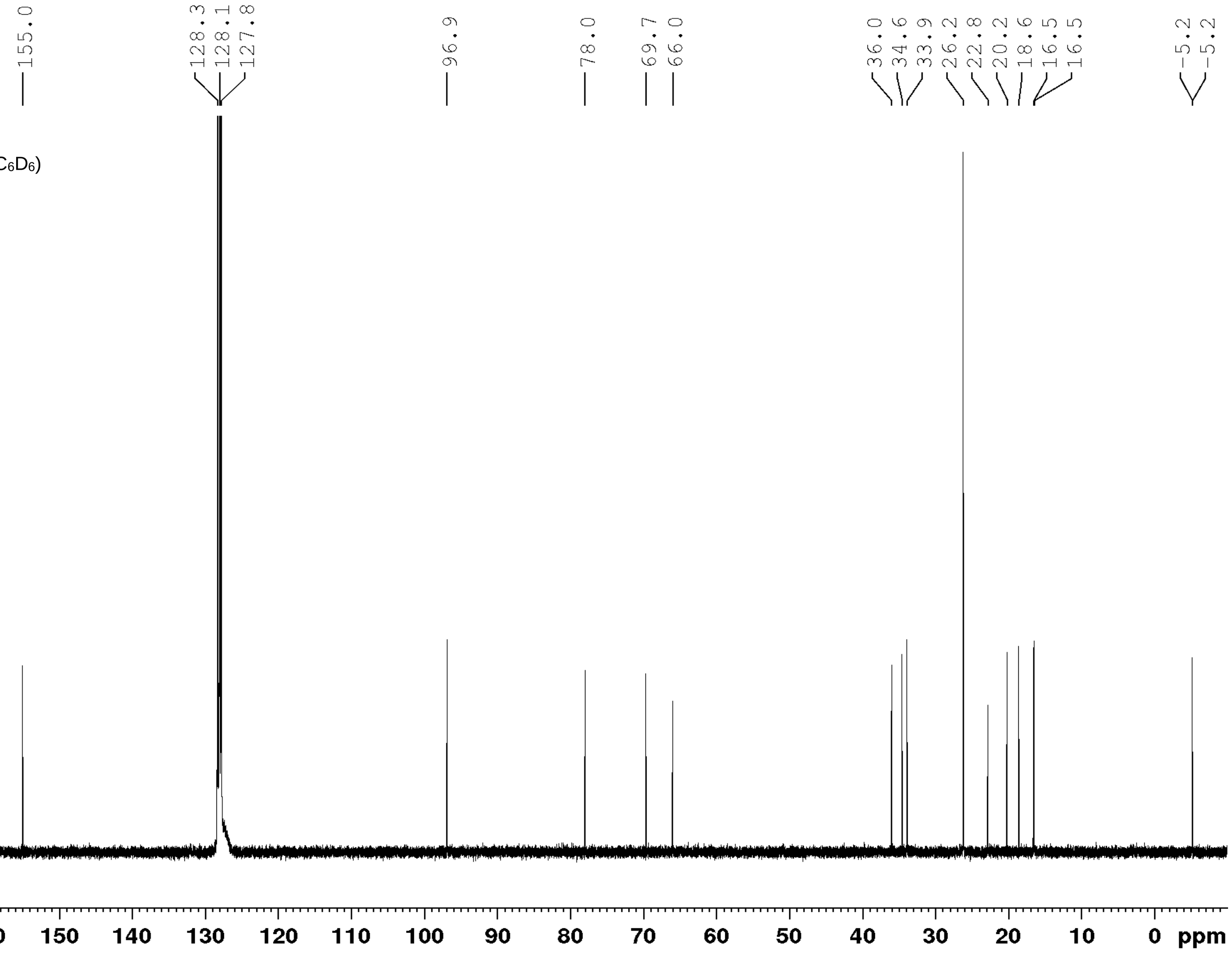

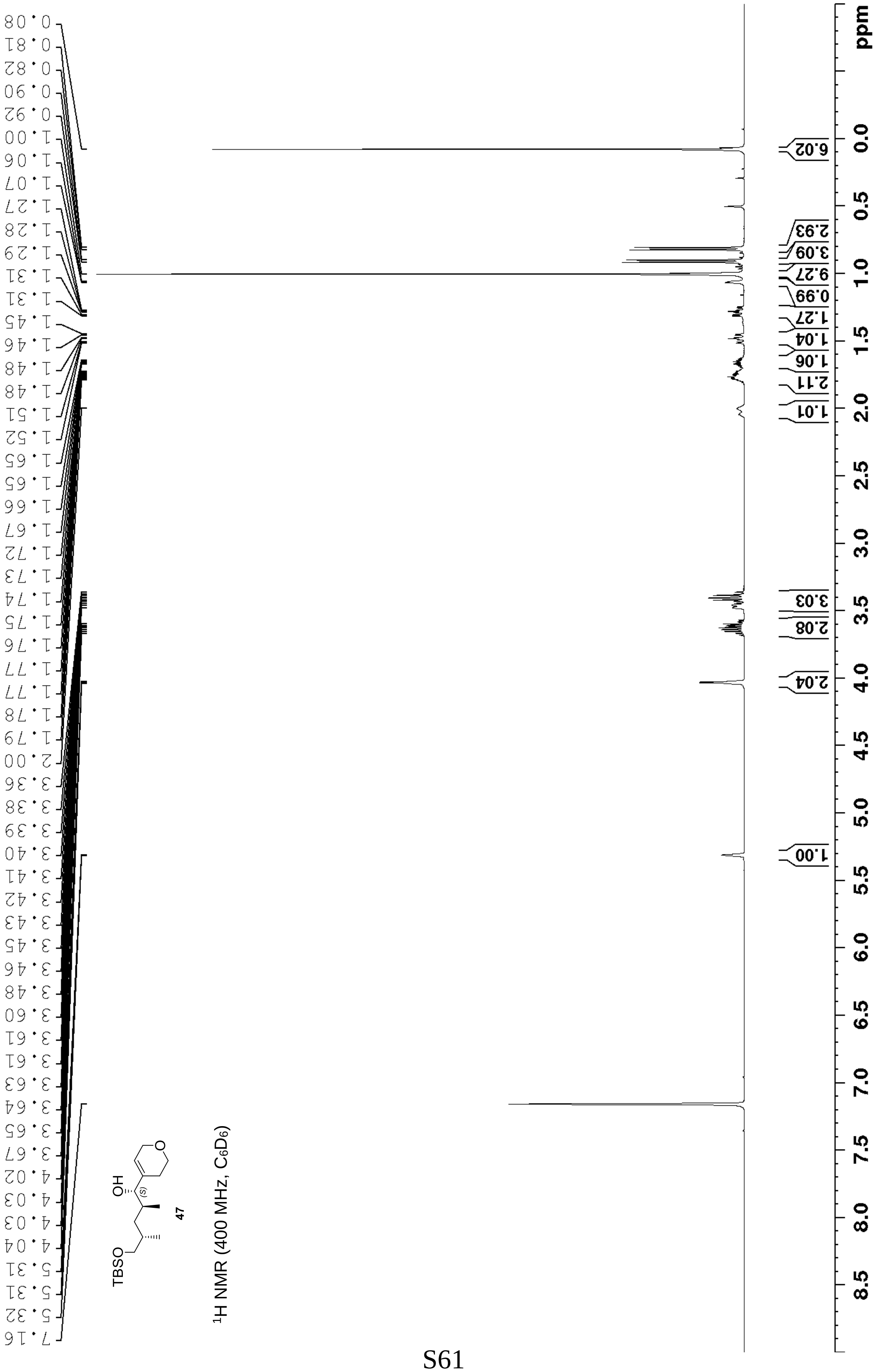
0.08 0.81 0.82 0.90 0.92 1.00 1.06 1.07 1.27 1.28 1.29 1.31 1.31 1.45 1.46 1.48 1.48 1.51 1.52 1.65 1.65 1.66 1.67 1.72 1.73 1.74 1.75 1.76 1.77 1.77 1.78 1.79 2.00 3.36 3.38 3.39 3.40 3.41 3.42 3.43 3.45 3.46 3.48 3.60 3.61 3.61 3.63 3.64 3.65 3.67 4.02 4.03 4.03 4.04 5.31 5.31 5.32 7.16
6.02
2.93
3.09
9.27
0.99
1.27
1.04
1.06
2.11
1.01
3.03
2.08
2.04
1.00
ppm
0.0 0.5 1.0 1.5 2.0 2.5 3.0 3.5 4.0 4.5 5.0 5.5 6.0 6.5 7.0 7.5 8.0 8.5
TBSO
OH
(S)
O
47
1H NMR (400 MHz, C6D6)

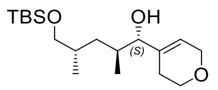

47

$^{13}C\{^{1}H\}$ NMR (101 MHz, C_6D_6)

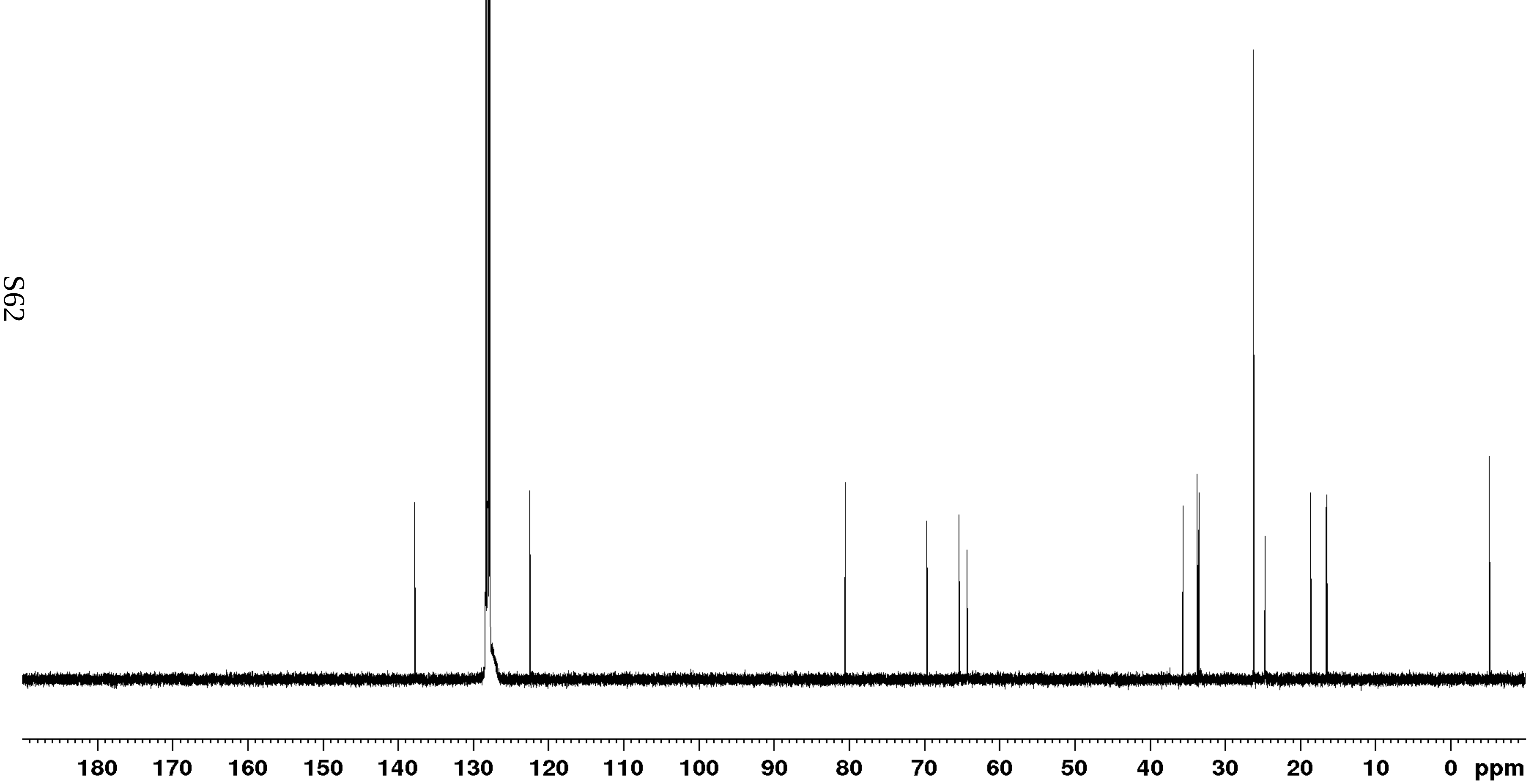

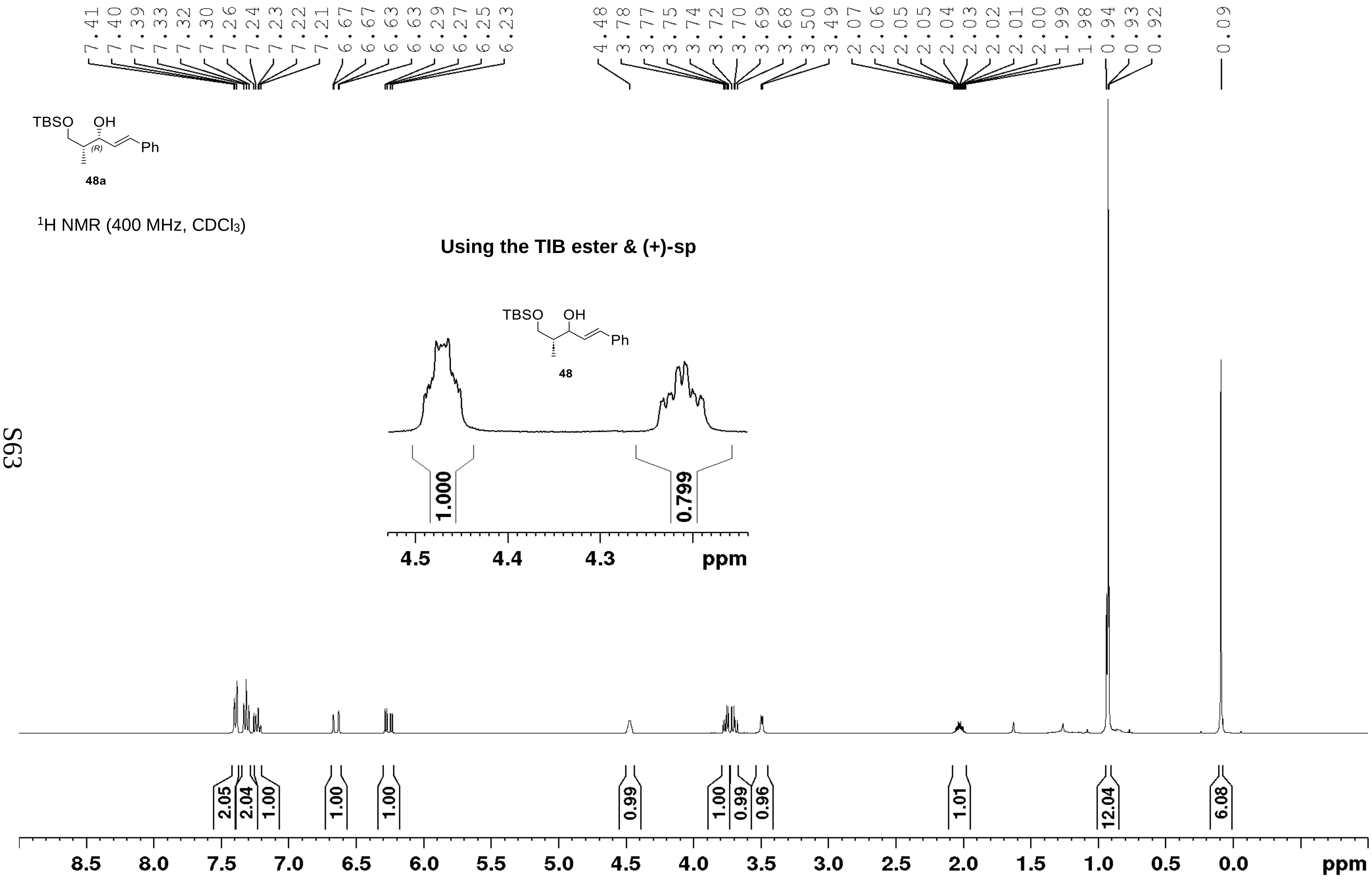
7.41 7.40 7.39 7.33 7.32 7.30 7.26 7.24 7.23 7.22 7.21 6.67 6.67 6.63 6.63 6.29 6.27 6.25 6.23
4.48 3.78 3.77 3.75 3.74 3.72 3.70 3.69 3.68 3.50 3.49 2.07 2.06 2.05 2.05 2.04 2.03 2.02 2.01 2.00 1.99 1.98 0.94 0.93 0.92
0.09
TBSO
OH
(R)
Ph
48a
¹H NMR (400 MHz, CDCl₃)
Using the TIB ester & (+)-sp
TBSO
OH
Ph
48
1.000
0.799
4.5
4.4
4.3
ppm
2.05
2.04
1.00
1.00
1.00
0.99
1.00
0.99
0.96
1.01
12.04
6.08
8.5 8.0 7.5 7.0 6.5 6.0 5.5 5.0 4.5 4.0 3.5 3.0 2.5 2.0 1.5 1.0 0.5 0.0
ppm

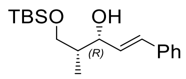

48a

$^{13}C\{^1H\}$ NMR (101 MHz, $CDCl_3$)

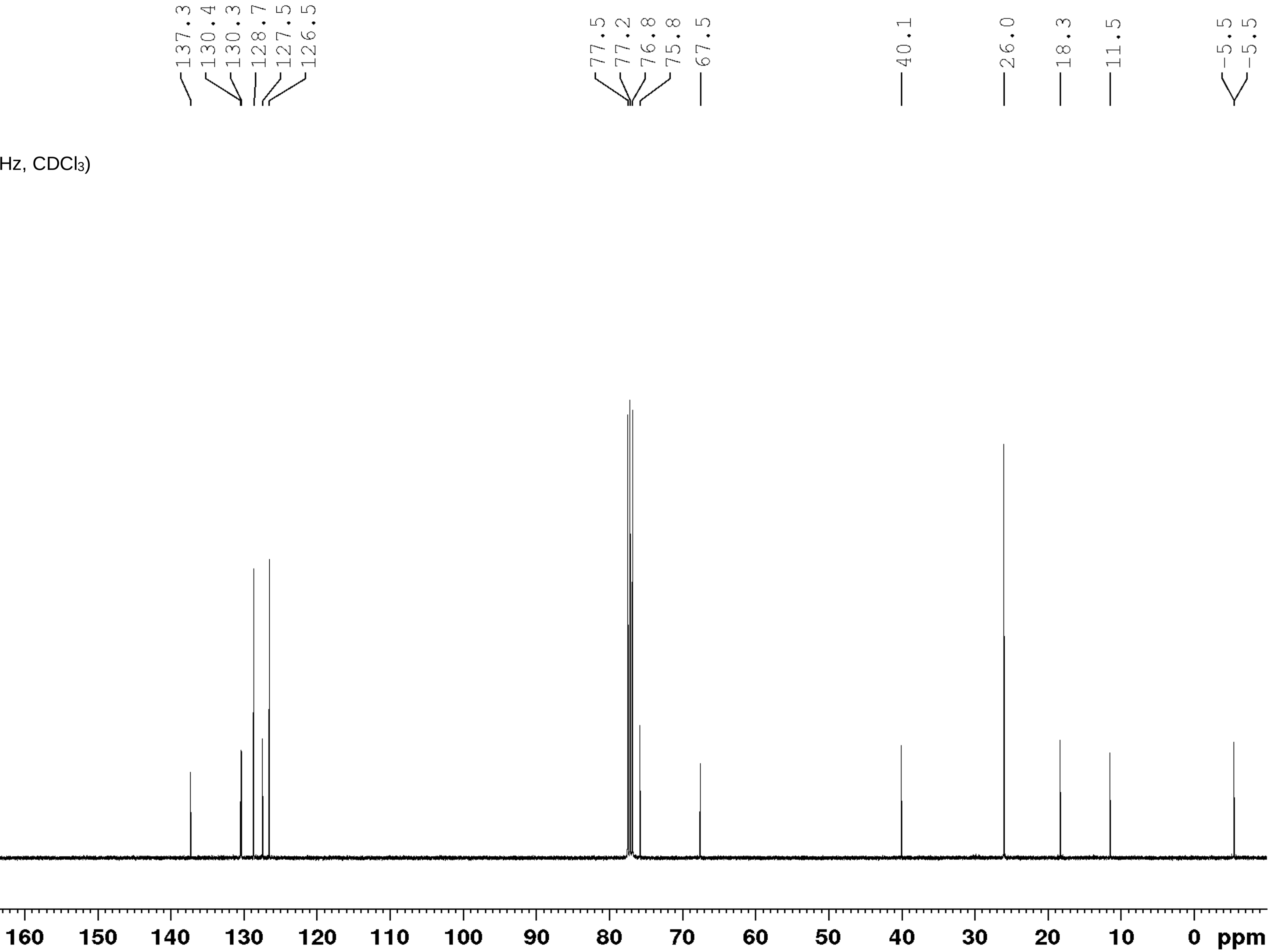

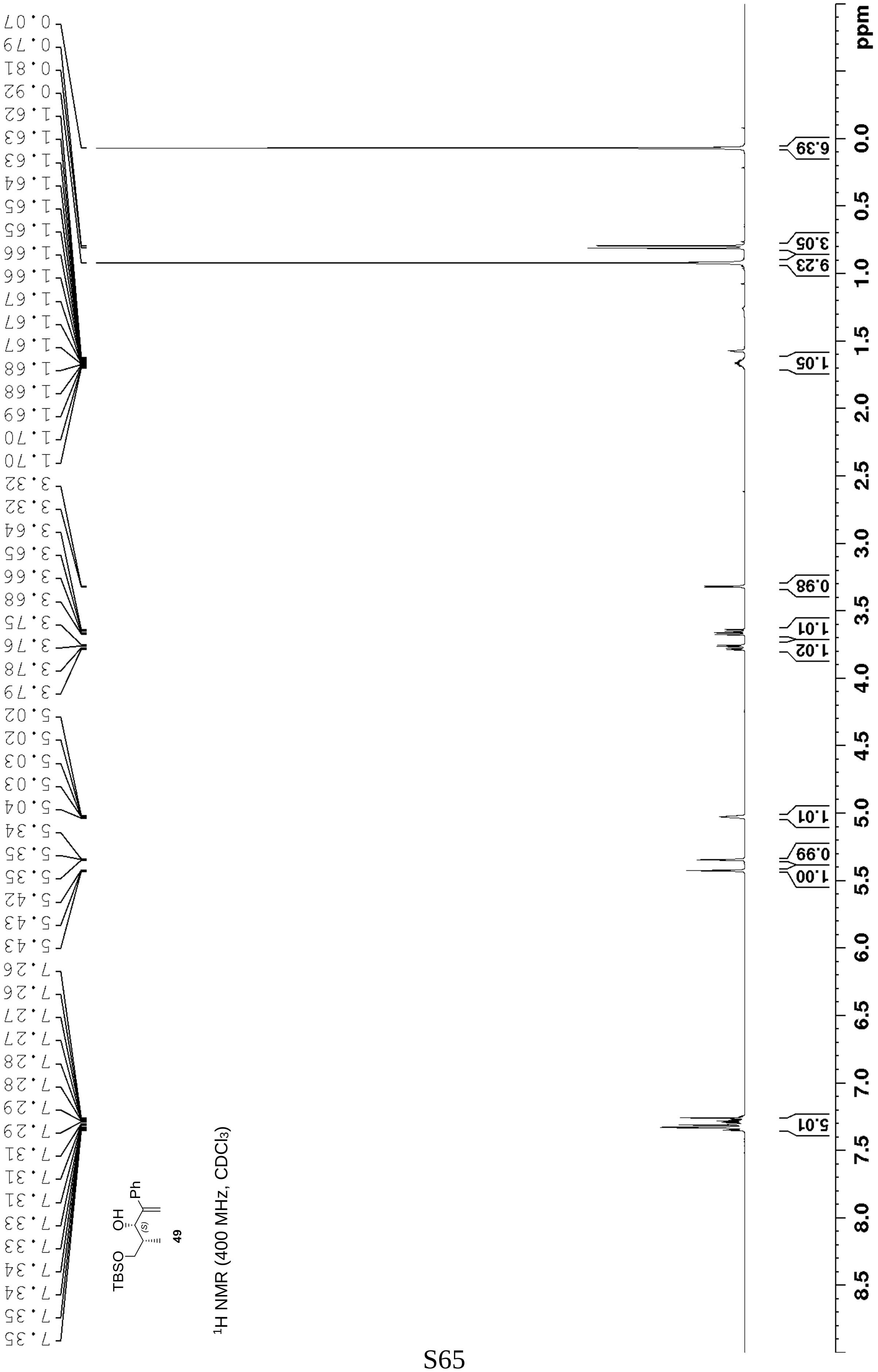

^{1}H NMR (400 MHz, $CDCl_3$)

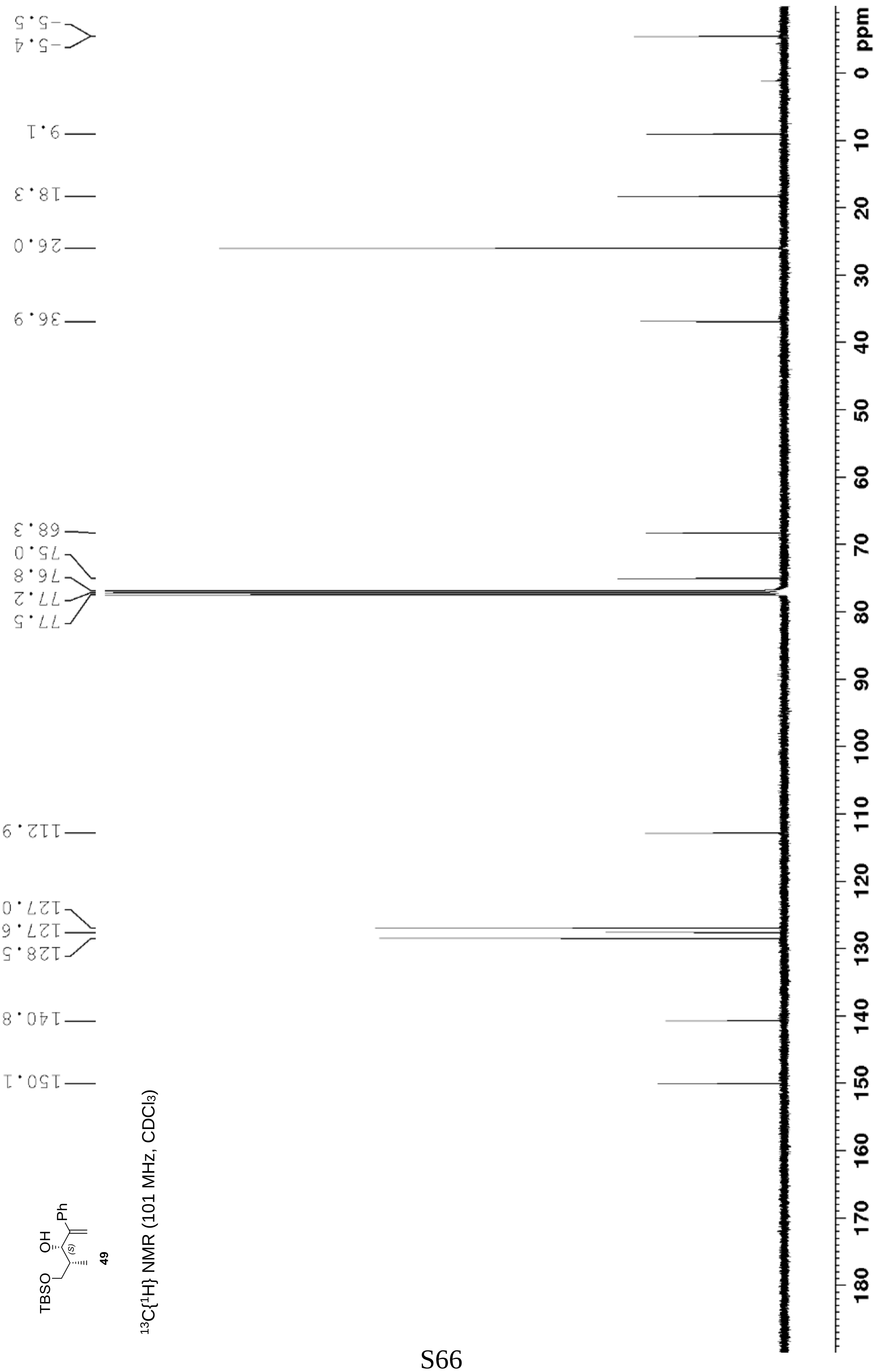

$^{13}C\{^1H\}$ NMR (101 MHz, $CDCl_3$)

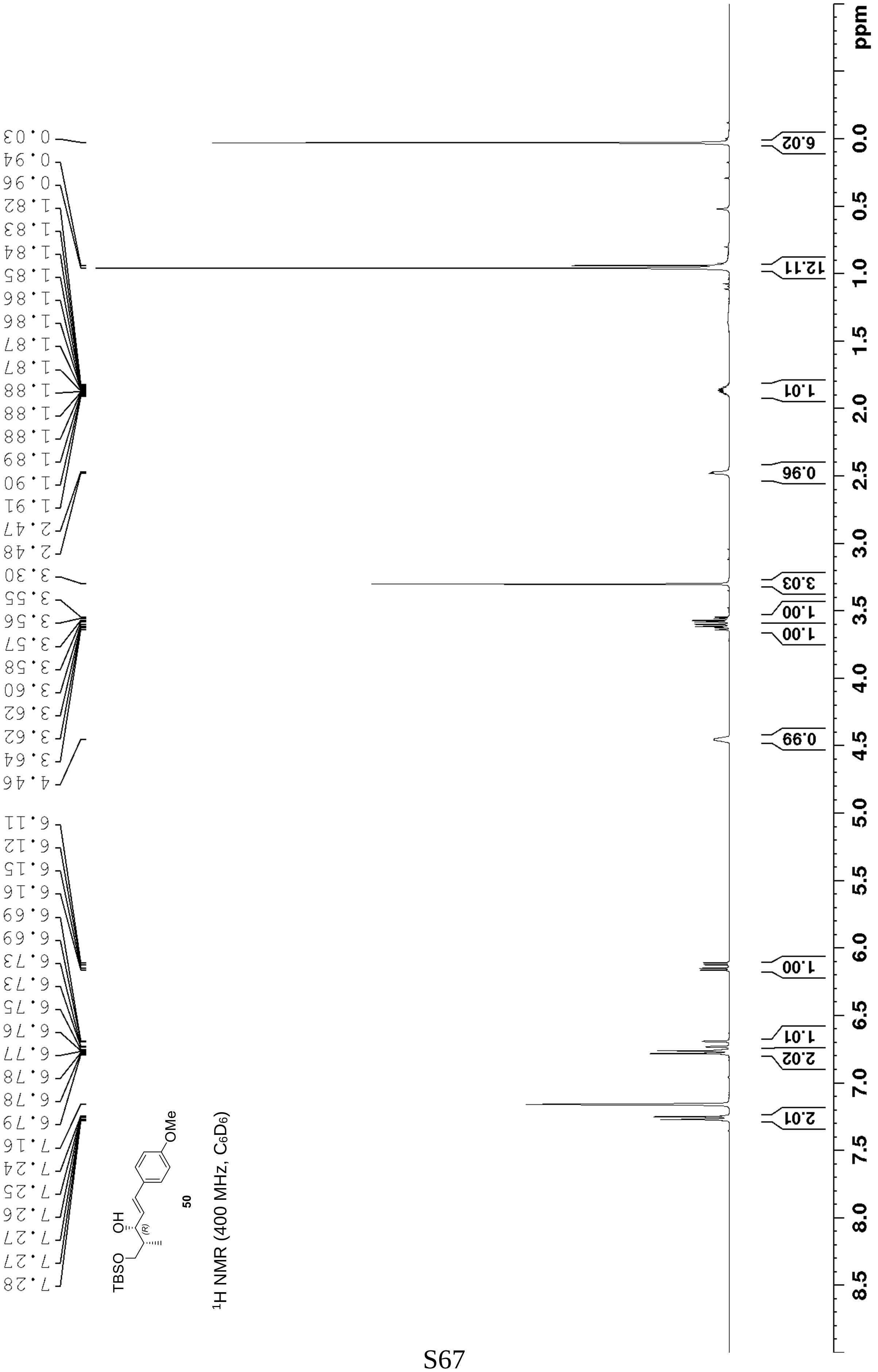
TBSO
OH
OMe
50
1H NMR (400 MHz, C6D6)
ppm

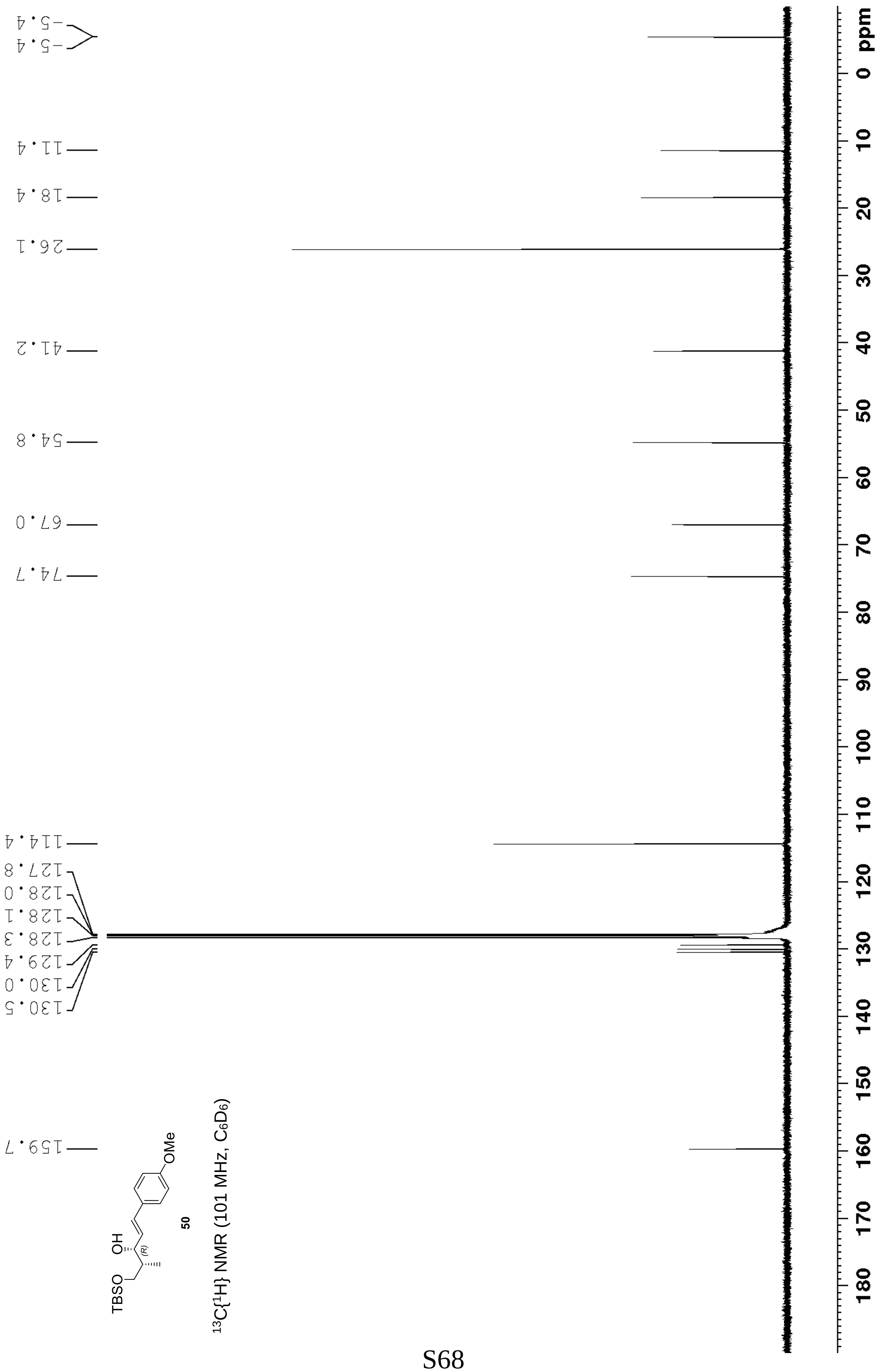
-5.4
-5.4
11.4
18.4
26.1
41.2
54.8
67.0
74.7
114.4
127.8
128.0
128.1
128.3
129.4
130.0
130.5
159.7
TBSO
OH
(R)
OMe
50
$^{13}C\{^1H\}$ NMR (101 MHz, C_6D_6)
ppm
0
10
20
30
40
50
60
70
80
90
100
110
120
130
140
150
160
170
180

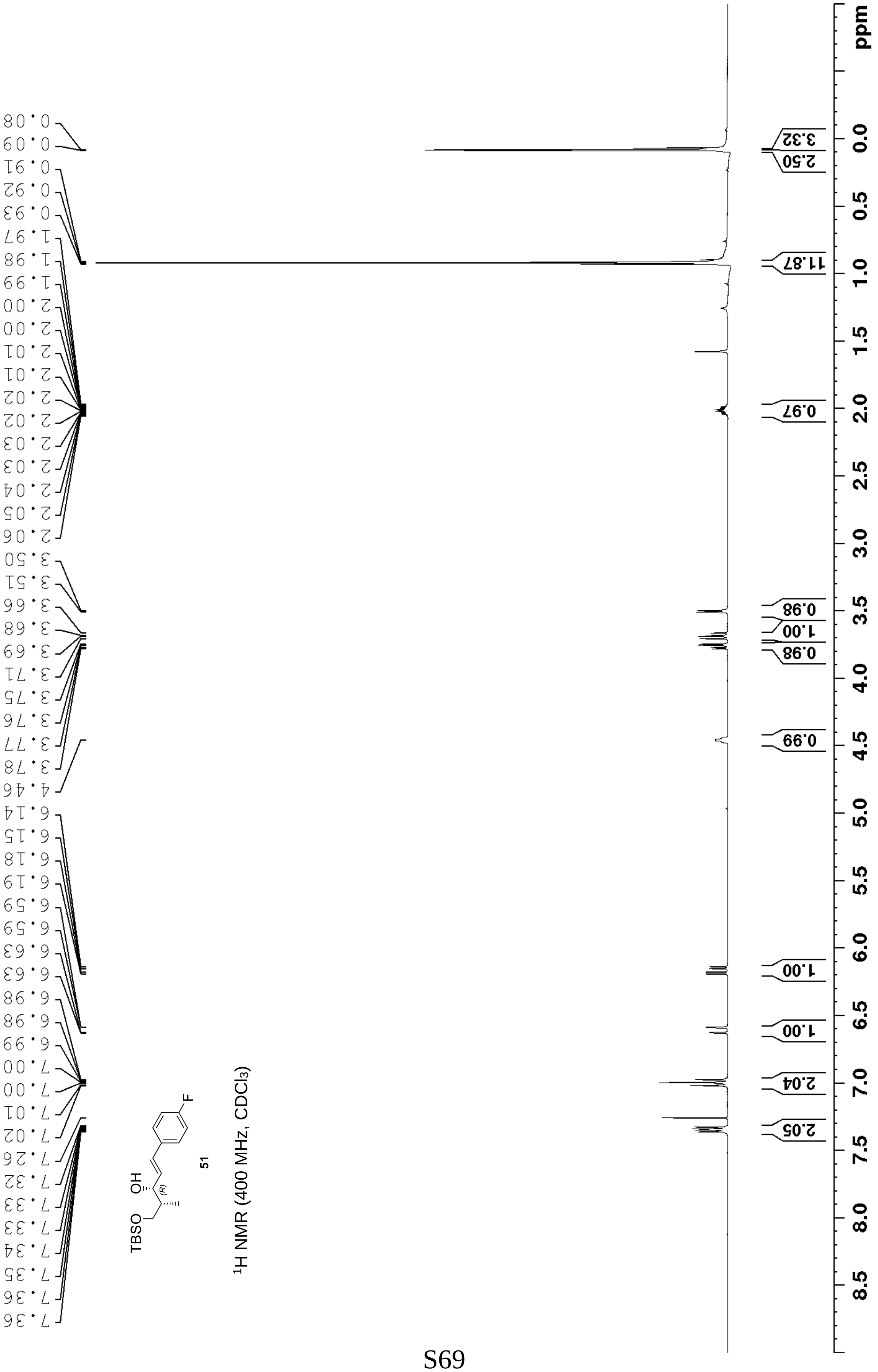

^{1}H NMR (400 MHz, $CDCl_3$)

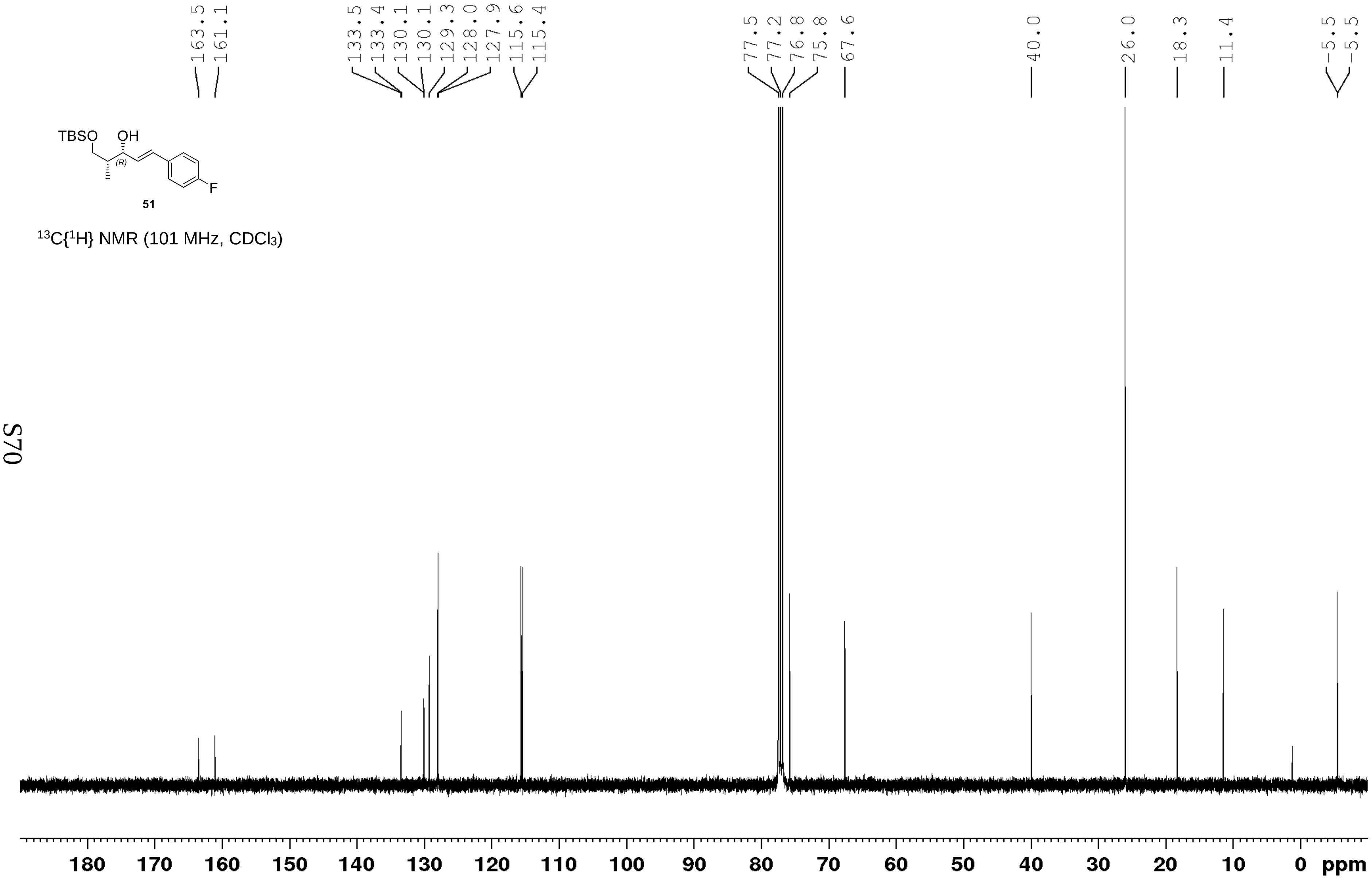

51

$^{13}C\{^{1}H\}$ NMR (101 MHz, $CDCl_3$)

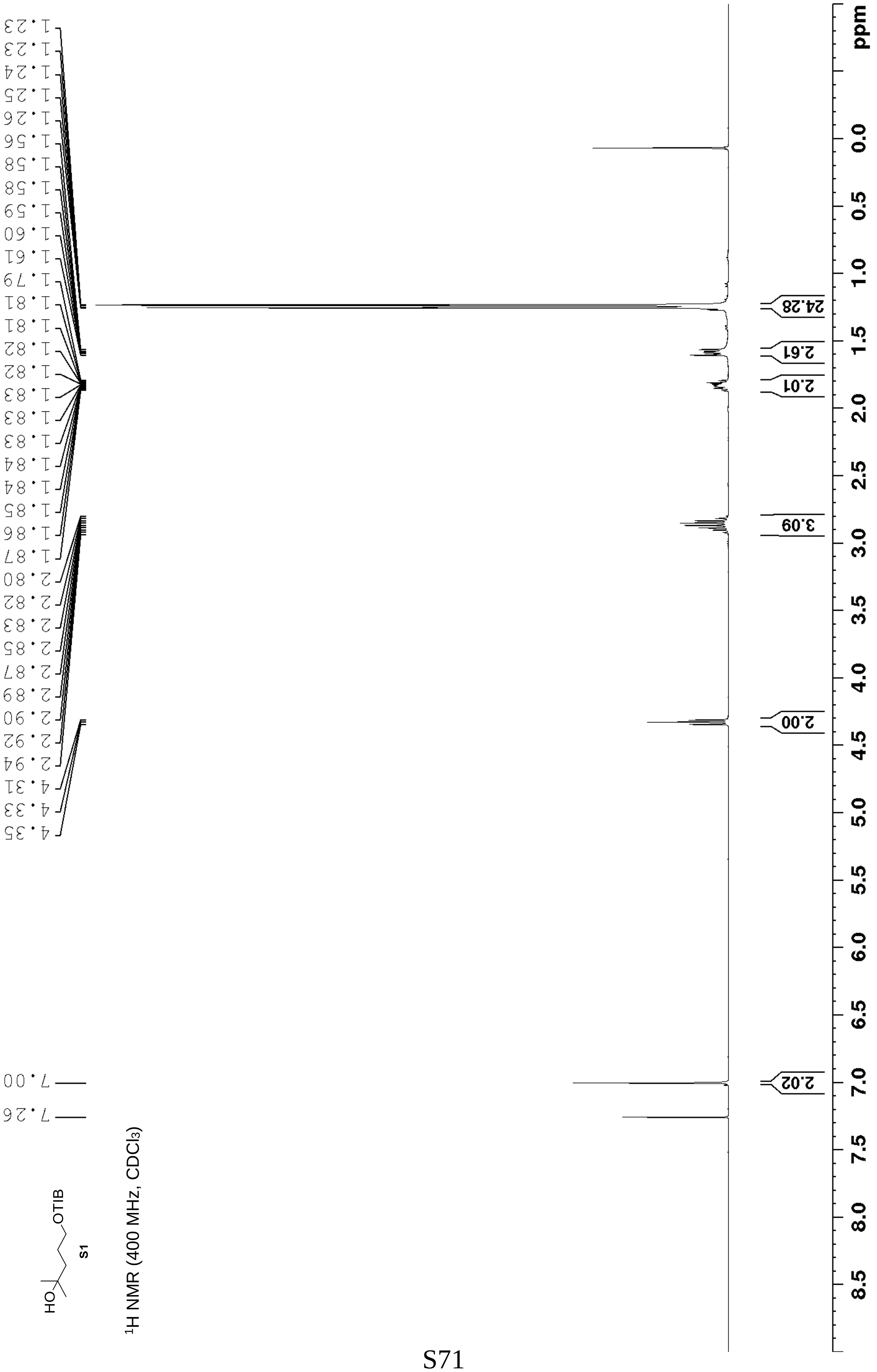
1.23
1.23
1.24
1.25
1.26
1.56
1.58
1.58
1.59
1.60
1.61
1.79
1.81
1.81
1.82
1.82
1.83
1.83
1.83
1.84
1.84
1.85
1.86
1.87
2.80
2.82
2.83
2.85
2.87
2.89
2.90
2.92
2.94
4.31
4.33
4.35
7.00
7.26
24.28
2.61
2.01
3.09
2.00
2.02
ppm
0.0
0.5
1.0
1.5
2.0
2.5
3.0
3.5
4.0
4.5
5.0
5.5
6.0
6.5
7.0
7.5
8.0
8.5
HO
OTIB
S1
^{1}H NMR (400 MHz, CDCl$_3$)

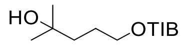

S1

$^{13}C\{^1H\}$ NMR (101 MHz, $CDCl_3$)

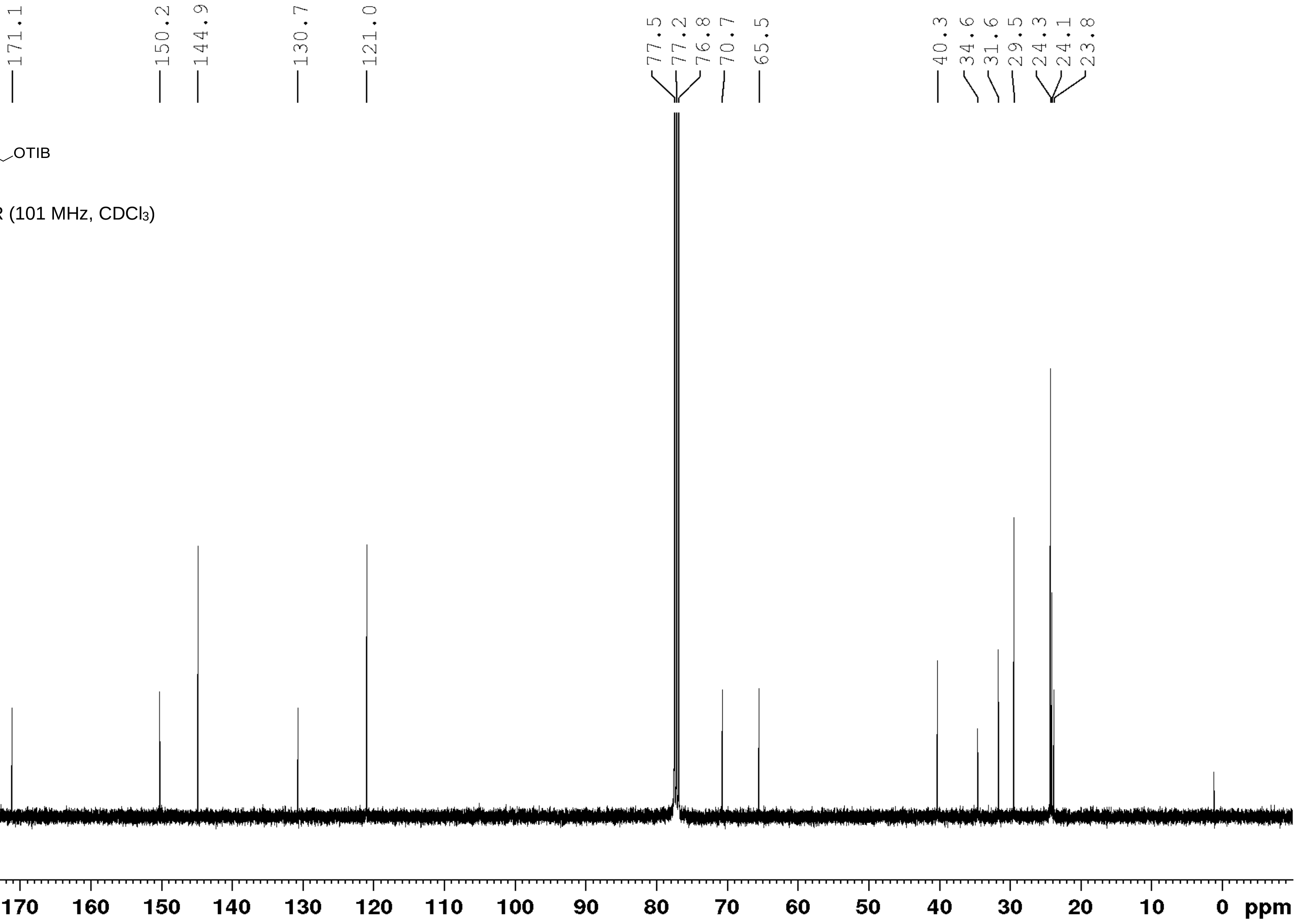

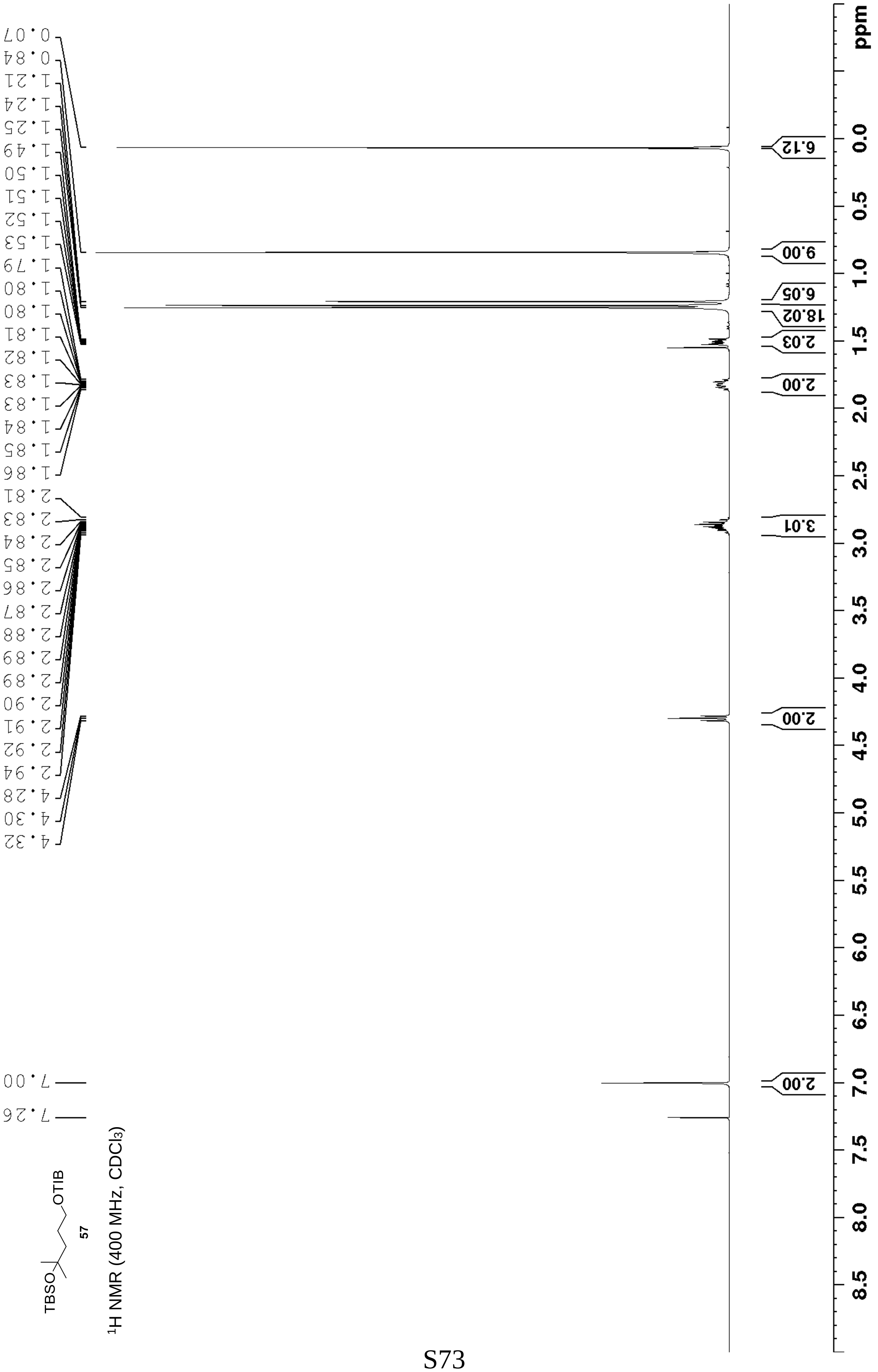

^1H NMR (400 MHz, $CDCl_3$)

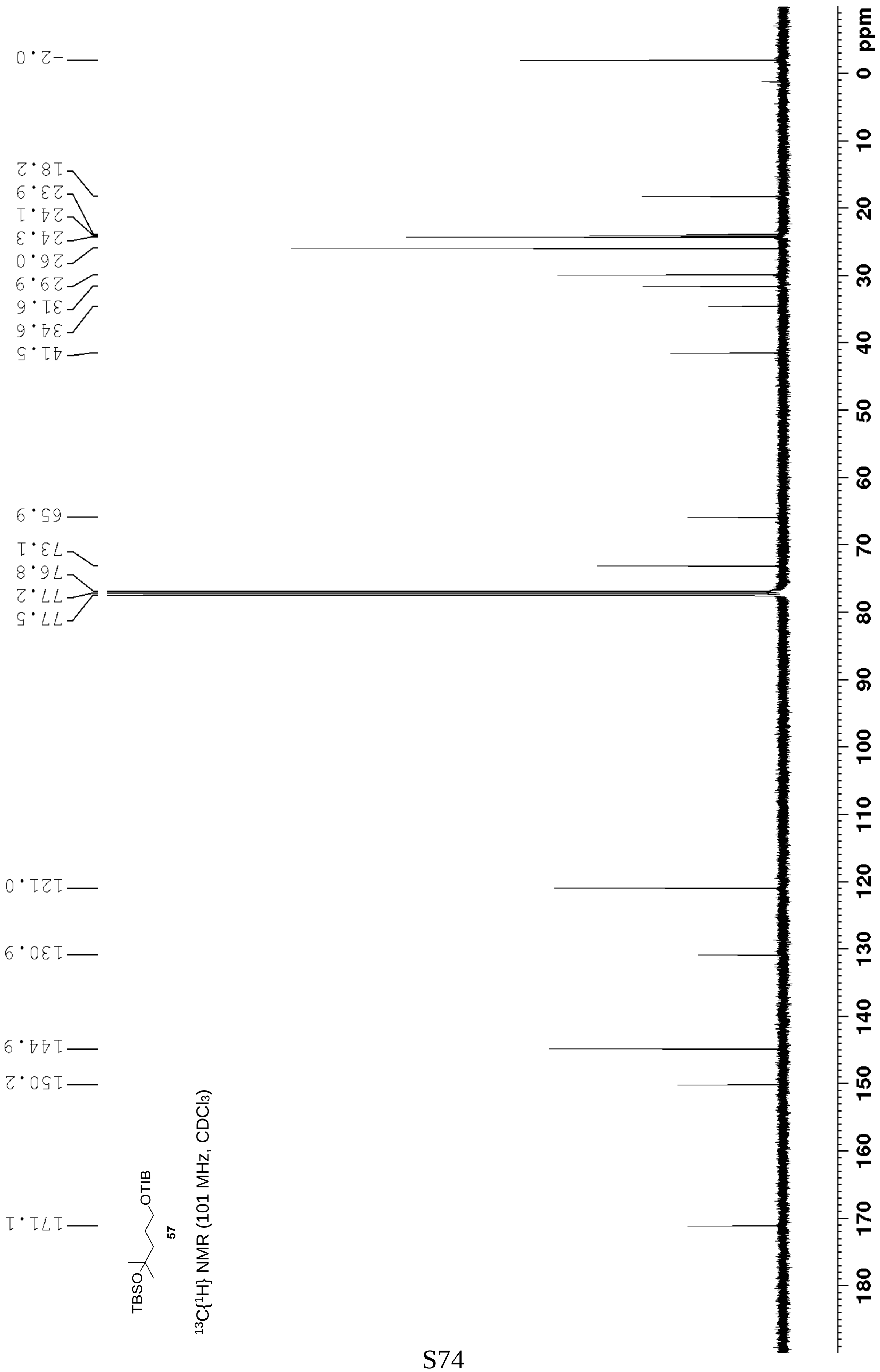

-2.0
18.2
23.9
24.1
24.3
26.0
29.9
31.6
34.6
41.5
65.9
73.1
76.8
77.2
77.5
121.0
130.9
144.9
150.2
171.1
TBSO
OTIB
57
$^{13}C\{^1H\}$ NMR (101 MHz, $CDCl_3$)
ppm
0
10
20
30
40
50
60
70
80
90
100
110
120
130
140
150
160
170
180

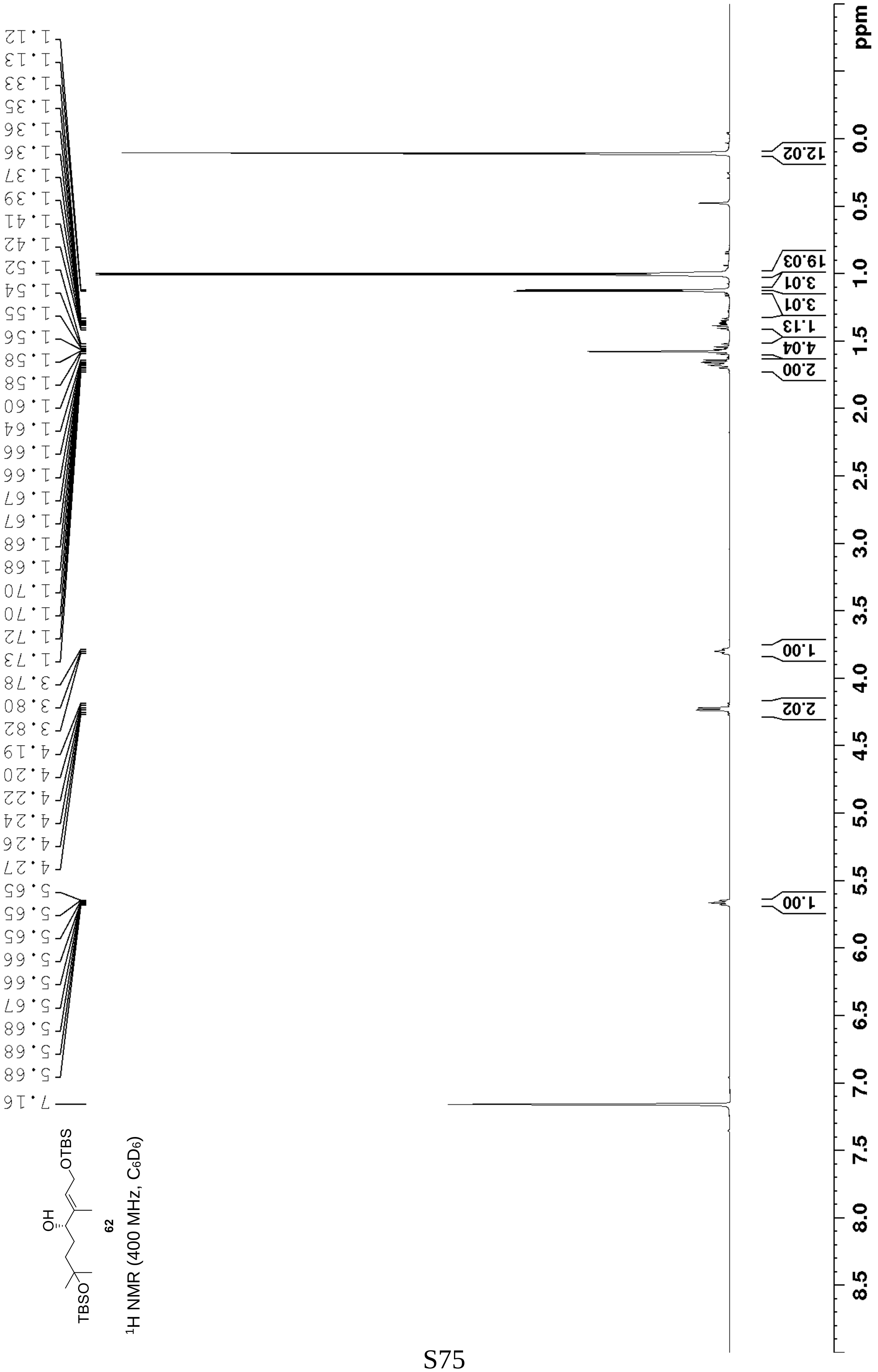

62
1H NMR (400 MHz, C6D6)
TBSO
OH
OTBS
7.16
5.68
5.68
5.68
5.67
5.66
5.66
5.65
5.65
5.65
4.27
4.26
4.24
4.22
4.20
4.19
3.82
3.80
3.78
1.73
1.72
1.70
1.70
1.68
1.68
1.67
1.67
1.66
1.66
1.64
1.60
1.58
1.58
1.56
1.55
1.54
1.52
1.42
1.41
1.39
1.37
1.36
1.36
1.35
1.33
1.13
1.12
1.00
2.02
1.00
2.00
4.04
1.13
3.01
3.01
19.03
12.02
ppm

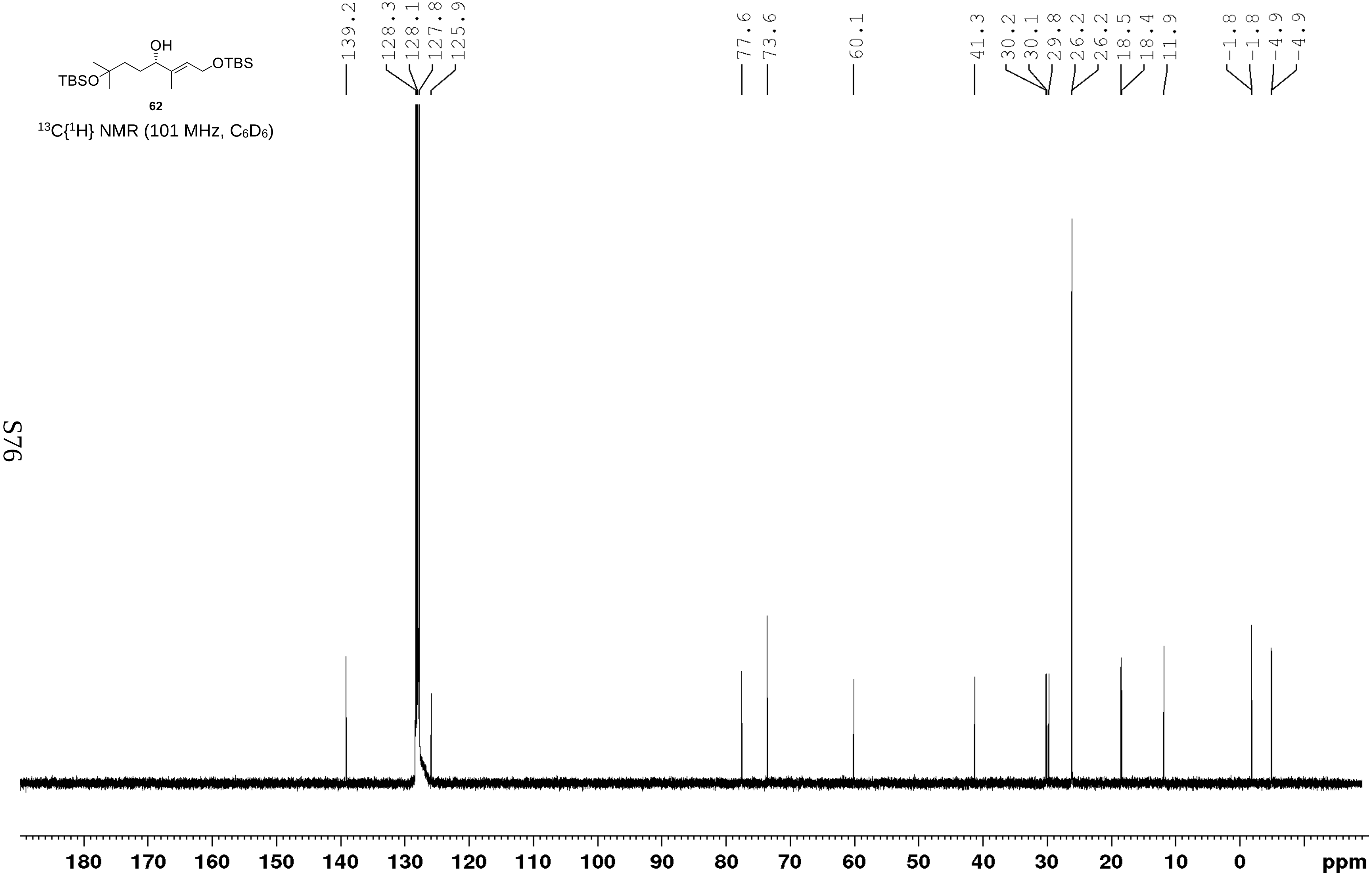

$^{13}C\{^{1}H\}$ NMR (101 MHz, C_6D_6)

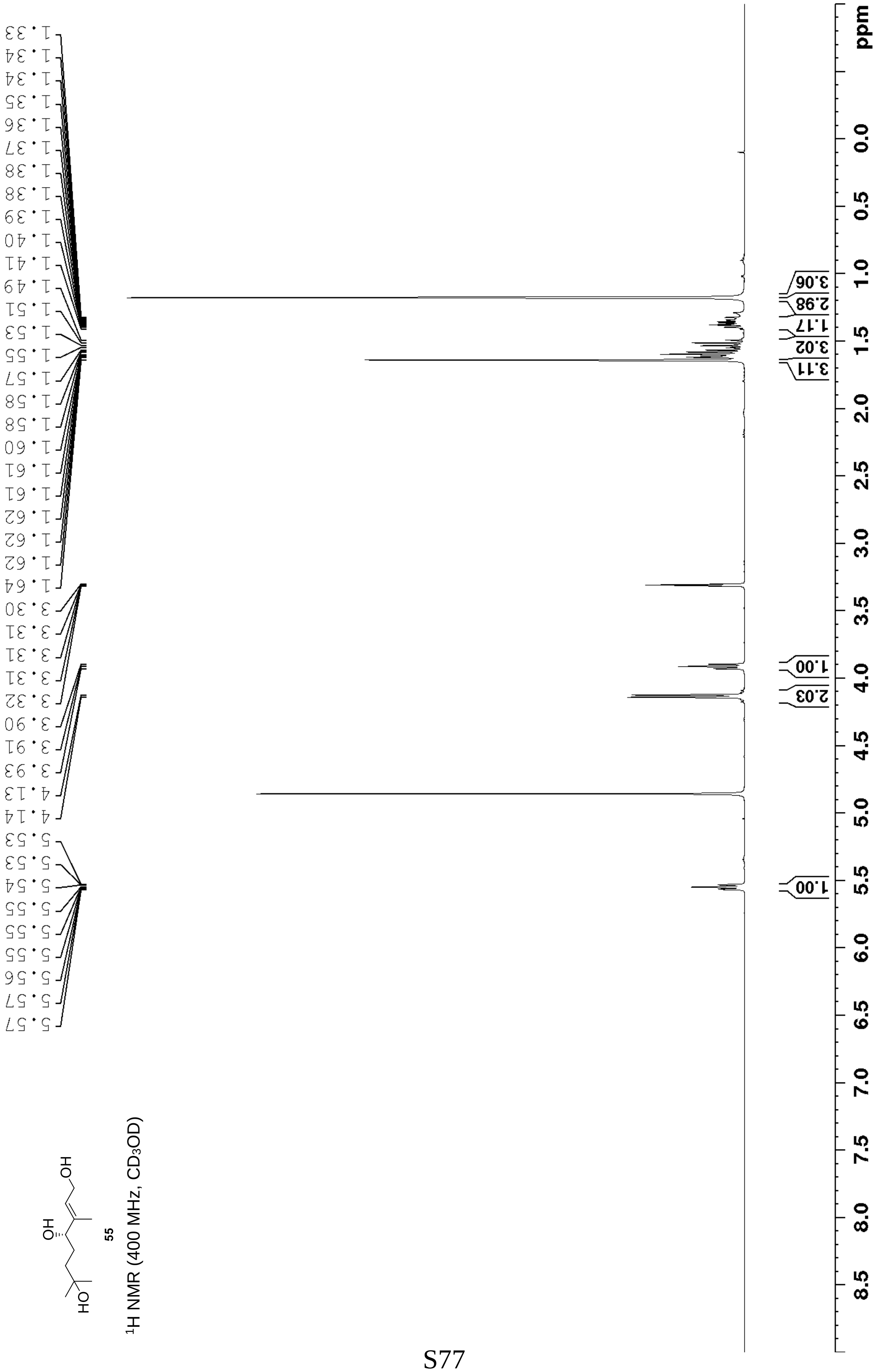

55

^{1}H NMR (400 MHz, CD_3OD)

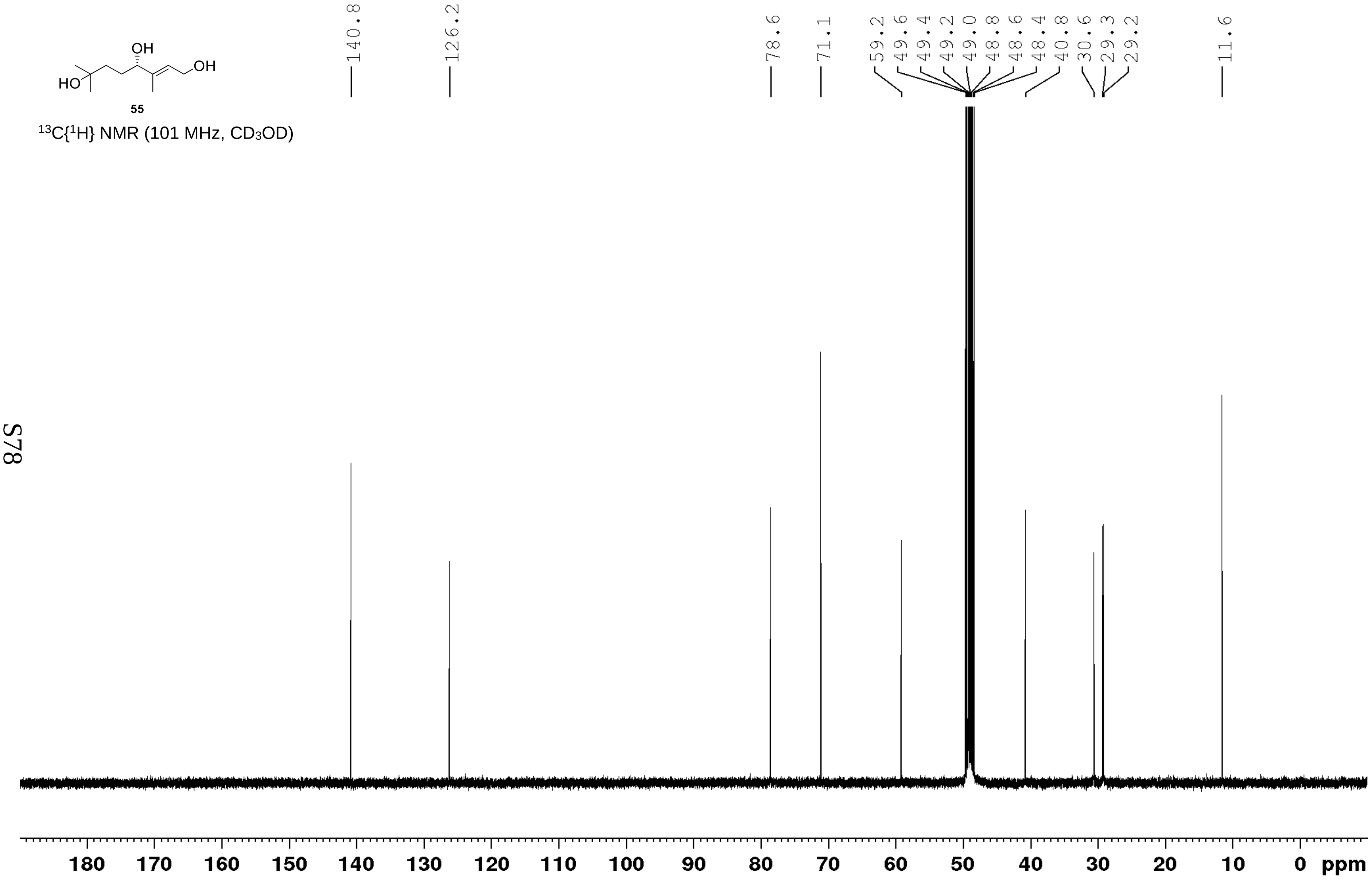

55

$^{13}C\{^{1}H\}$ NMR (101 MHz, CD_3OD)

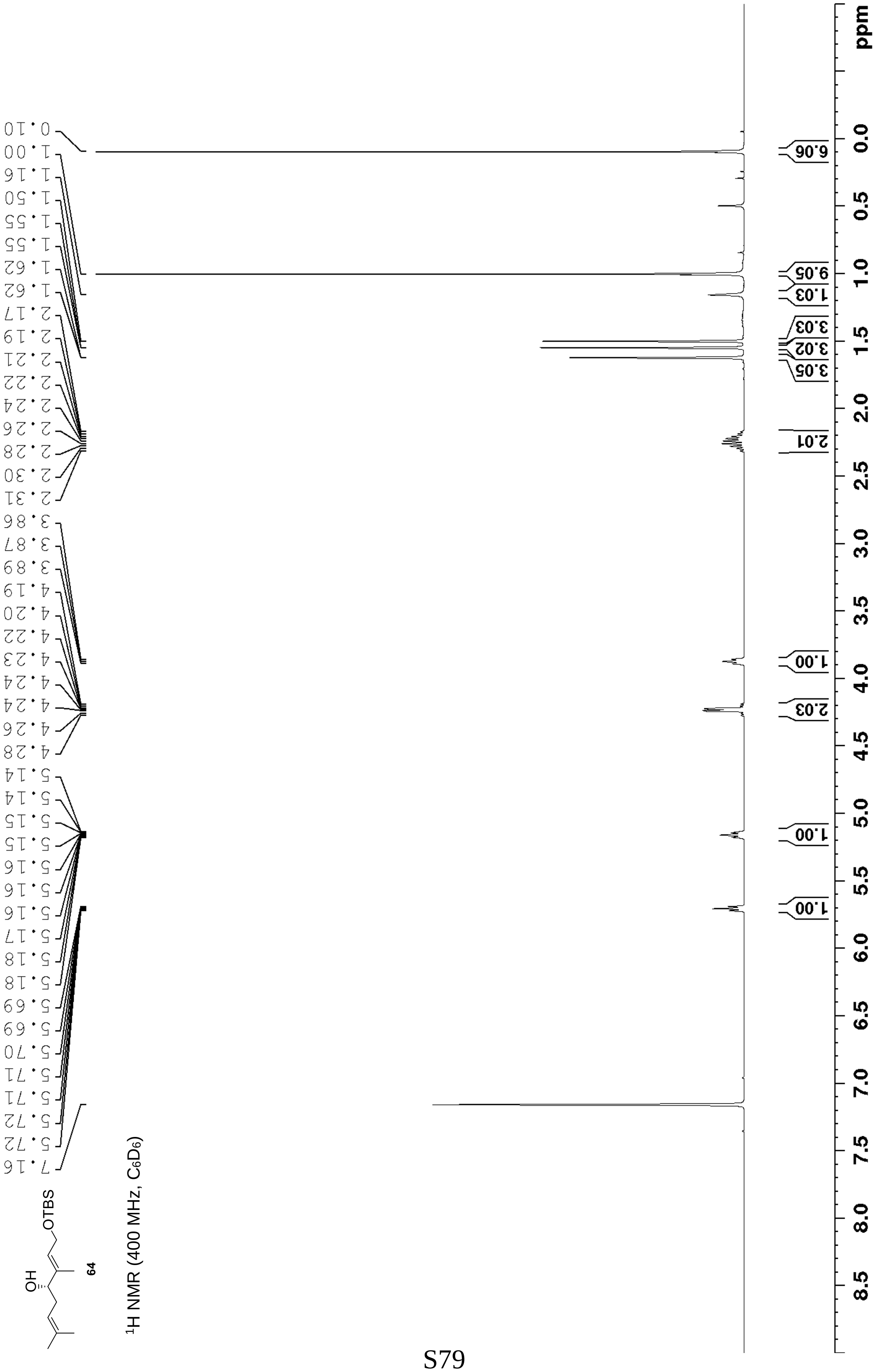
OH
OTBS
64
1H NMR (400 MHz, C6D6)
0.10
1.00
1.16
1.50
1.55
1.55
1.62
1.62
2.17
2.19
2.21
2.22
2.24
2.26
2.28
2.30
2.31
3.86
3.87
3.89
4.19
4.20
4.22
4.23
4.24
4.24
4.26
4.28
5.14
5.14
5.15
5.15
5.16
5.16
5.16
5.17
5.18
5.18
5.69
5.69
5.70
5.71
5.71
5.72
5.72
7.16
6.06
9.05
1.03
3.03
3.02
3.05
2.01
1.00
2.03
1.00
1.00
ppm
0.0
0.5
1.0
1.5
2.0
2.5
3.0
3.5
4.0
4.5
5.0
5.5
6.0
6.5
7.0
7.5
8.0
8.5

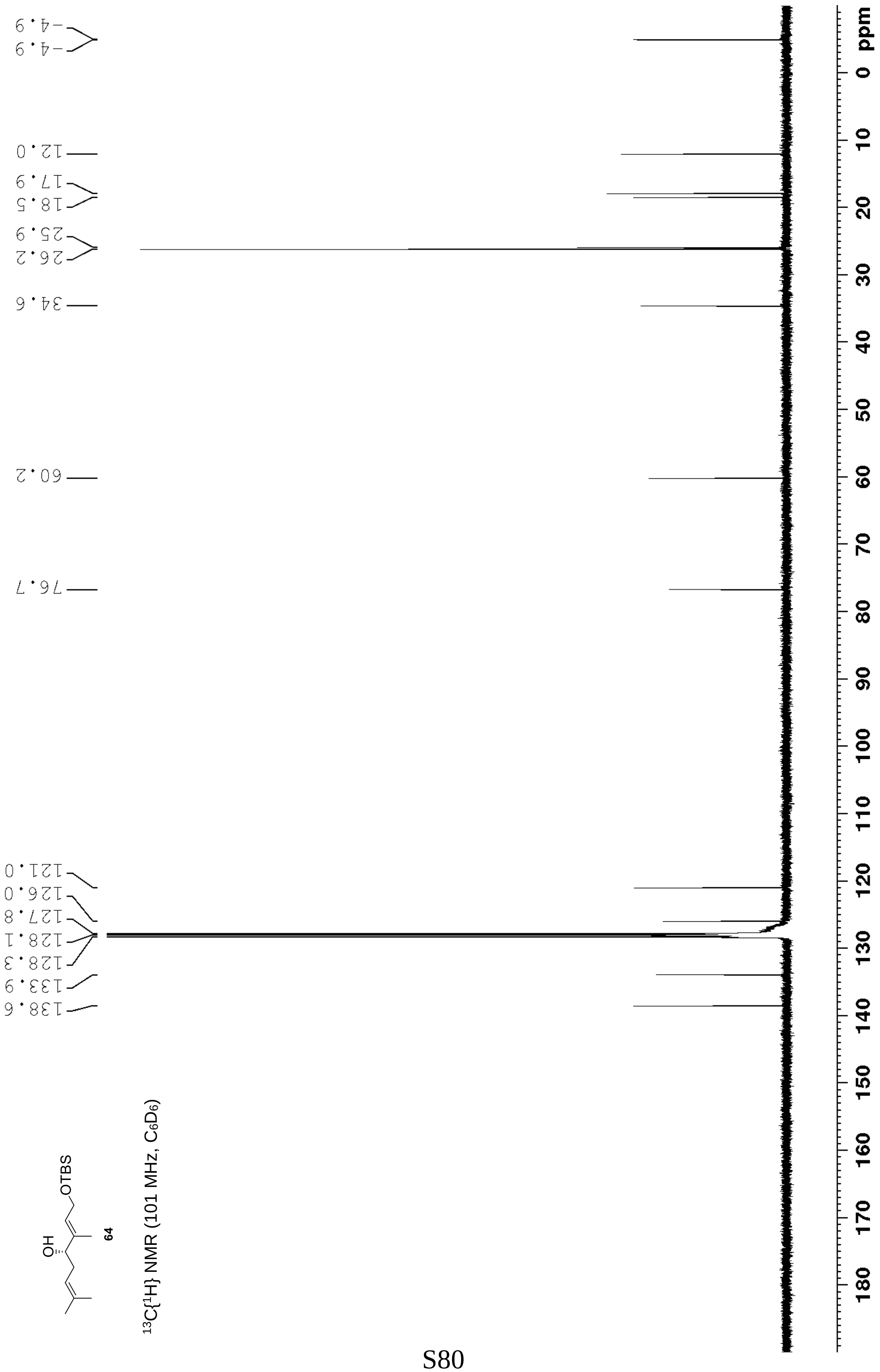

-4.9
-4.9
12.0
17.9
18.5
25.9
26.2
34.6
60.2
76.7
121.0
126.0
127.8
128.1
128.3
133.9
138.6
OH
OTBS
64
$^{13}C\{^1H\}$ NMR (101 MHz, C_6D_6)
ppm
0
10
20
30
40
50
60
70
80
90
100
110
120
130
140
150
160
170
180

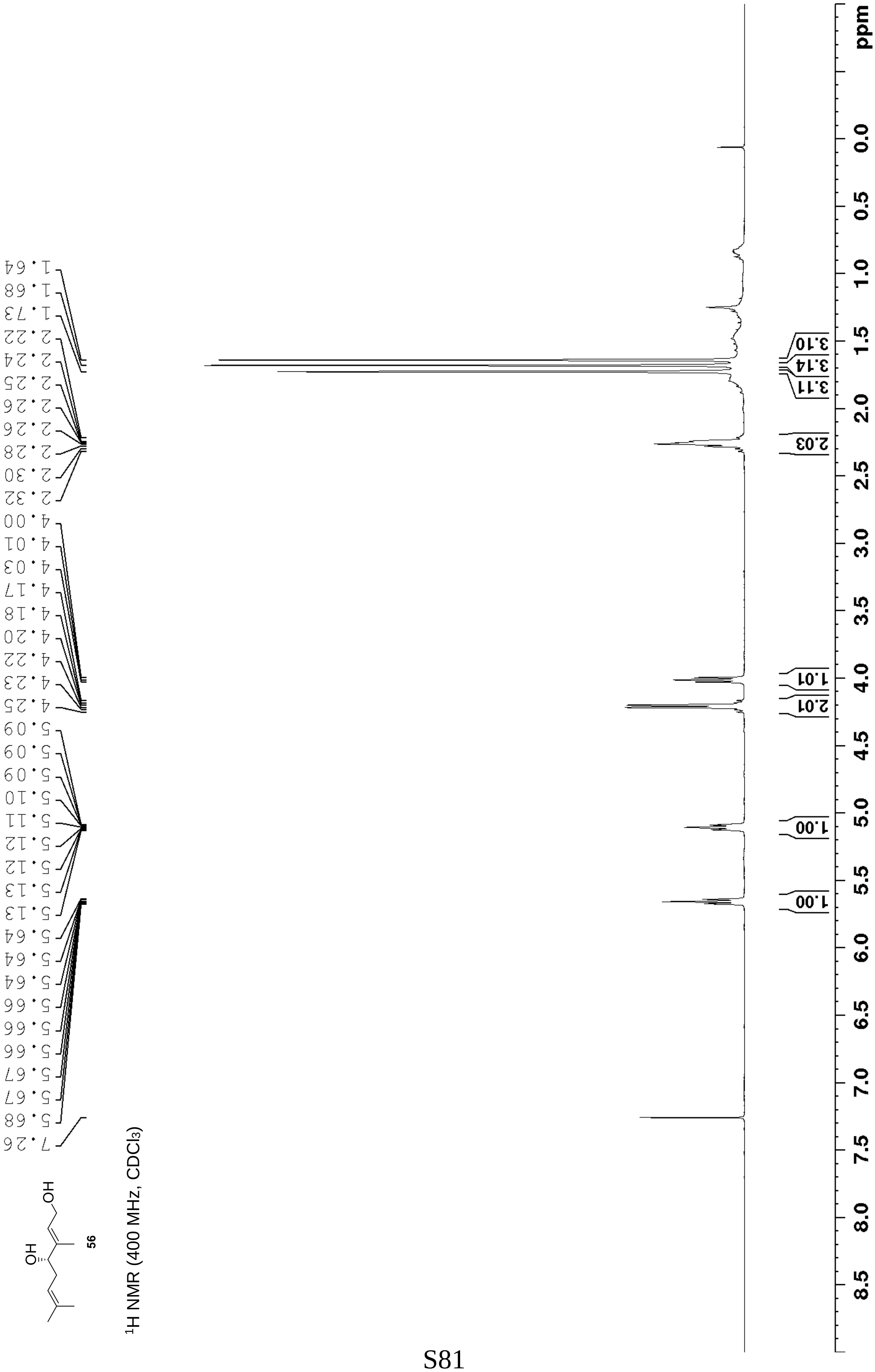

^{1}H NMR (400 MHz, $CDCl_3$)

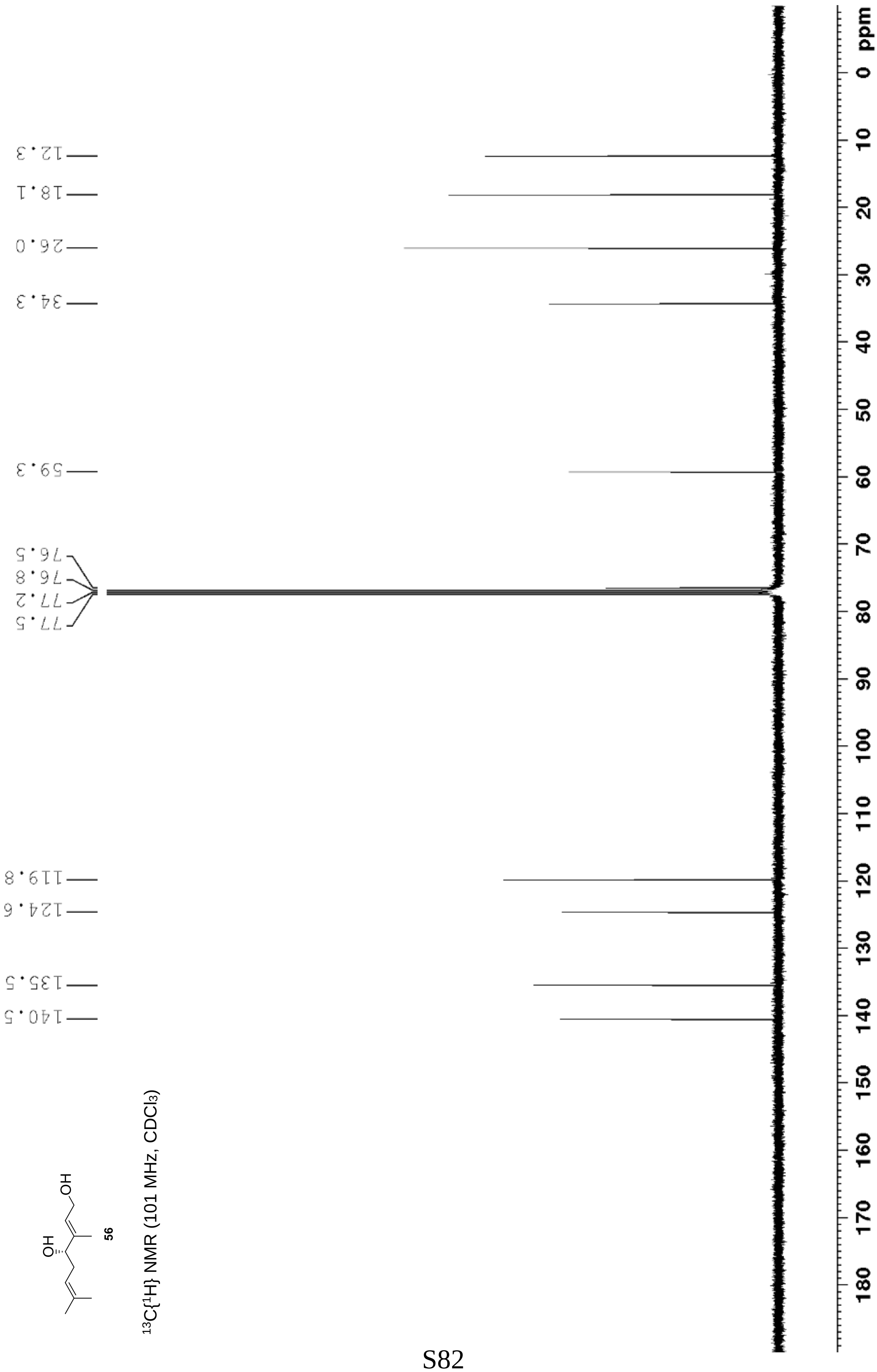

$^{13}C\{^1H\}$ NMR (101 MHz, $CDCl_3$)

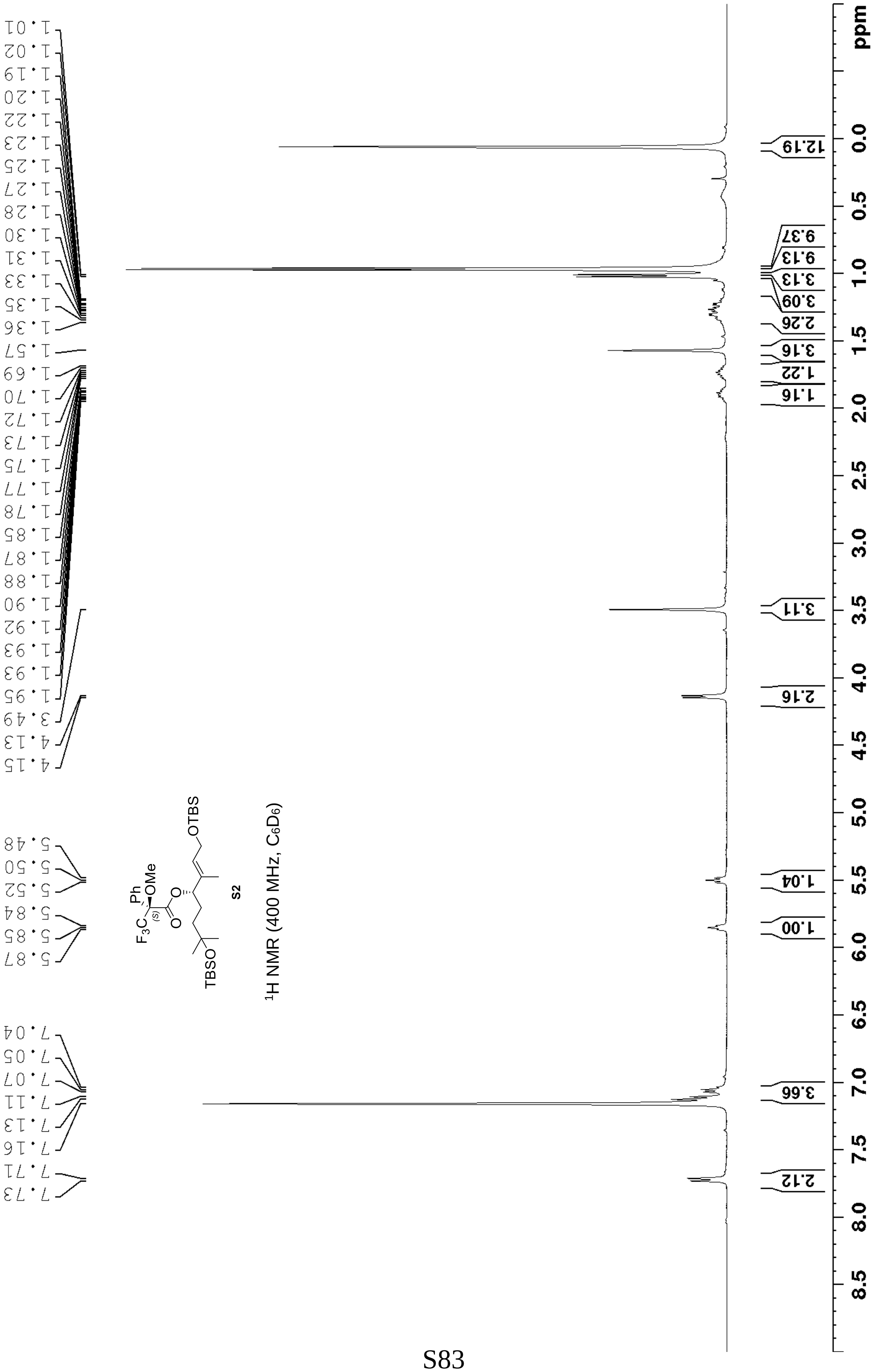
1.01
1.02
1.19
1.20
1.22
1.23
1.25
1.27
1.28
1.30
1.31
1.33
1.35
1.36
1.57
1.69
1.70
1.72
1.73
1.75
1.77
1.78
1.85
1.87
1.88
1.90
1.92
1.93
1.93
1.95
3.49
4.13
4.15
5.48
5.50
5.52
5.84
5.85
5.87
7.04
7.05
7.07
7.11
7.13
7.16
7.71
7.73
12.19
9.37
9.13
3.13
3.09
2.26
3.16
1.22
1.16
3.11
2.16
1.04
1.00
3.66
2.12
ppm
0.0
0.5
1.0
1.5
2.0
2.5
3.0
3.5
4.0
4.5
5.0
5.5
6.0
6.5
7.0
7.5
8.0
8.5
OTBS
Ph
OMe
F_3C
(S)
O
TBSO
S2
^1H NMR (400 MHz, C_6D_6)

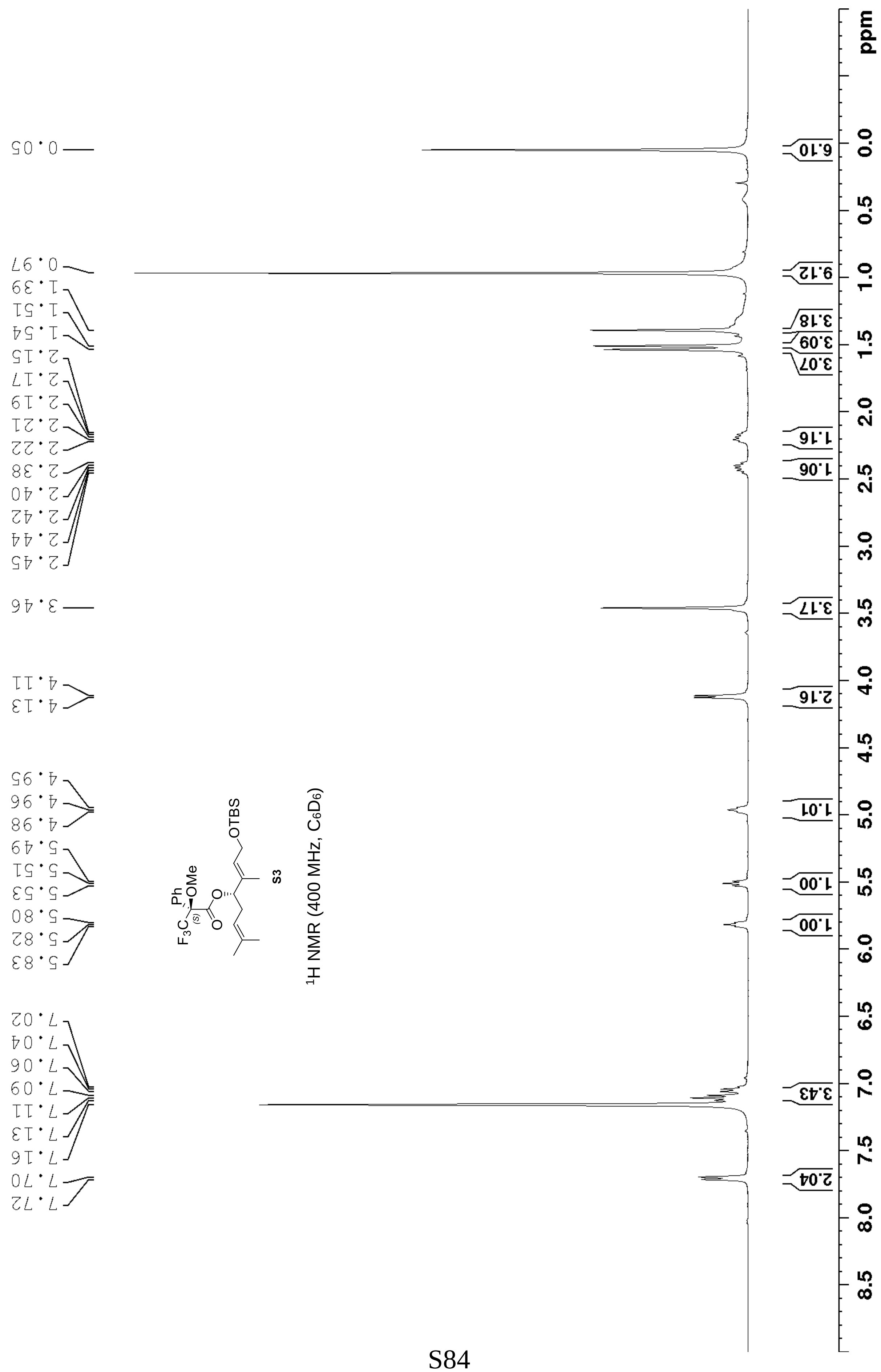
0.05
0.97
1.39
1.51
1.54
2.15
2.17
2.19
2.21
2.22
2.38
2.40
2.42
2.44
2.45
3.46
4.11
4.13
4.95
4.96
4.98
5.49
5.51
5.53
5.80
5.82
5.83
7.02
7.04
7.06
7.09
7.11
7.13
7.16
7.70
7.72
6.10
9.12
3.18
3.09
3.07
1.16
1.06
3.17
2.16
1.01
1.00
1.00
3.43
2.04
ppm
0.0
0.5
1.0
1.5
2.0
2.5
3.0
3.5
4.0
4.5
5.0
5.5
6.0
6.5
7.0
7.5
8.0
8.5
F3C
Ph
OMe
(S)
O
O
OTBS
S3
1H NMR (400 MHz, C6D6)
